# Design, synthesis and cytotoxic evaluation of novel betulonic acid-diazine derivatives as potential antitumor agents

**DOI:** 10.3389/fchem.2022.969770

**Published:** 2022-09-06

**Authors:** Yisong Shu, Feifei Li, Yaotian Han, Penglong Wang, Feng Gao, Mengmeng Yan, Miao Liang, Qiang Ma, Yuzhong Zhang, Xia Ding, Haimin Lei

**Affiliations:** ^1^ School of Chinese Materia Medica, Beijing University of Chinese Medicine, Beijing, China; ^2^ Chinese Academy of Inspection and Quarantine, Beijing, China; ^3^ School of Traditional Chinese Medicine, Beijing University of Chinese Medicine, Beijing, China

**Keywords:** betulonic acid-diazine derivatives, nitrogen-containing heterocycles, synthesis, cytotoxicity, metabolomics

## Abstract

With the purpose to improve antiproliferative activity, 26 new betulonic acid-diazine derivatives were designed and synthesized from betulinic acid. The anticancer activity of these semi-synthetic compounds was evaluated by MTT assay in both tumor cell lines and normal cell line. The results indicated that majority of new compounds exhibited improved antitumor activity compared with the parent compound betulonic acid. Compound **BoA2C**, in particular, had the most significant action with IC_50_ value of 3.39 μM against MCF-7 cells, while it showed lower cytotoxicity on MDCK cell line than cisplatin. Furthermore, we discovered that **BoA2C** strongly increased MCF-7 cell damage mostly by influencing arginine and fatty acid metabolism. In addition, the structure-activity relationships were briefly discussed. The results of this study suggested that the introduction of different diazines at C-28 could selectively inhibit different kinds of cancer cells and might be an effective way to synthesize potent anticancer lead compound from betulonic acid.

## 1 Introduction

Breast cancer (BC), as the most common cancer type among women, remains a major threat to the health and wellness accounting for nearly 30 percent of cancer deaths each year (Hendrick et al., 2021). Current treatments for BC are similar to those for other cancers, including surgery, chemotherapy, and radiation. These treatments are designed to eliminate tumors and improve the lives of patients. Chemotherapy, in particular, is still one of the most effective treatments in BC, interfering with tumor cell reproduction and proliferation using exclusive medications (Maughan et al., 2010). Betulinic acid (BA), which belongs to lupane-type pentacyclic triterpenoids, can arrest the cell cycles of BC at G1 phases to inhibit viability and migration of breast cancer cells (Damle et al., 2013; Hsu et al., 2015). It has been reported that oxidation of the hydroxyl group at position C-3 markedly enhanced the cytotoxic potency (Baratto et al., 2013; Zhang et al., 2014). Thus, betulonic acid (BoA), which had a ketone instead of a hydroxyl with β-configuration at the C-3 of BA, was used as a starting molecule in the synthetic transformations described in this research for the identification of novel anticancer drugs.

Nitrogen-containing heterocycles in the drug design are almost 60% of unique small-molecule drugs based on FDA database (Liu et al., 2019). In parallel, a large number of nitrogen-containing heterocyclic derivatives like pyrrole, pyrrolidine, pyridine, and pyrazine have been discovered and described for their antitumor properties (Taglieri et al., 2020). In this respect, the antineoplastic properties of BAM15, a furazano [3,4-b] pyrazine, was demonstrated in breast cancer through uncoupling oxidative phosphorylation from ATP production (Zunica et al., 2021). Furthermore (3R,4S)-3-(4-bromophenyl)-4-(4-fluorobenzoyl)-2-(2-oxo-2-phenylethyl)-3,4-dihydropyrrolo [1,2-a] pyrazin-2-ium bromide, synthesized based on pyrrolo [1,2-a] pyrazine, was suggested as a novel potential anticancer agent via caspase-3 activation and PARP cleavage (Seo et al., 2020). Previously, we had reported the preparation of a series of novel natural product derivatives with ligustrazine (2,3,5,6-tetramethylpyrazine, TMP) which was a major effective component of Ligusticum chuanxiong Hort and observed that these derivatives possessed potent selective cytotoxicity (Xu et al., 2015; Bi et al., 2016; Xu et al., 2017; Wang H. et al., 2018; Wang P. et al., 2018; Fang et al., 2018). Meanwhile, we synthesized hederagenin derivatives containing pyrazines with different methyl numbers and positions, and most of these derivatives showed much stronger cytotoxicity than the parent compound (Fang et al., 2018).

**FIGURE 1:**
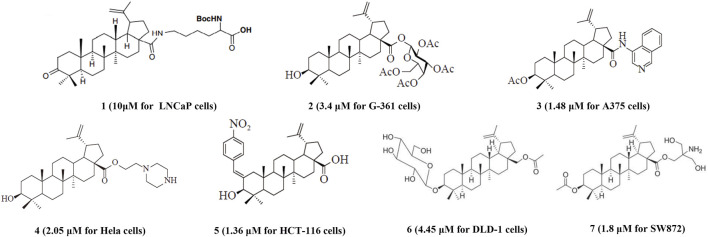
Structures of representative BoA derivatives with antitumor activity. The IC_50_ values of these compounds are shown in parentheses.

**FIGURE 2:**
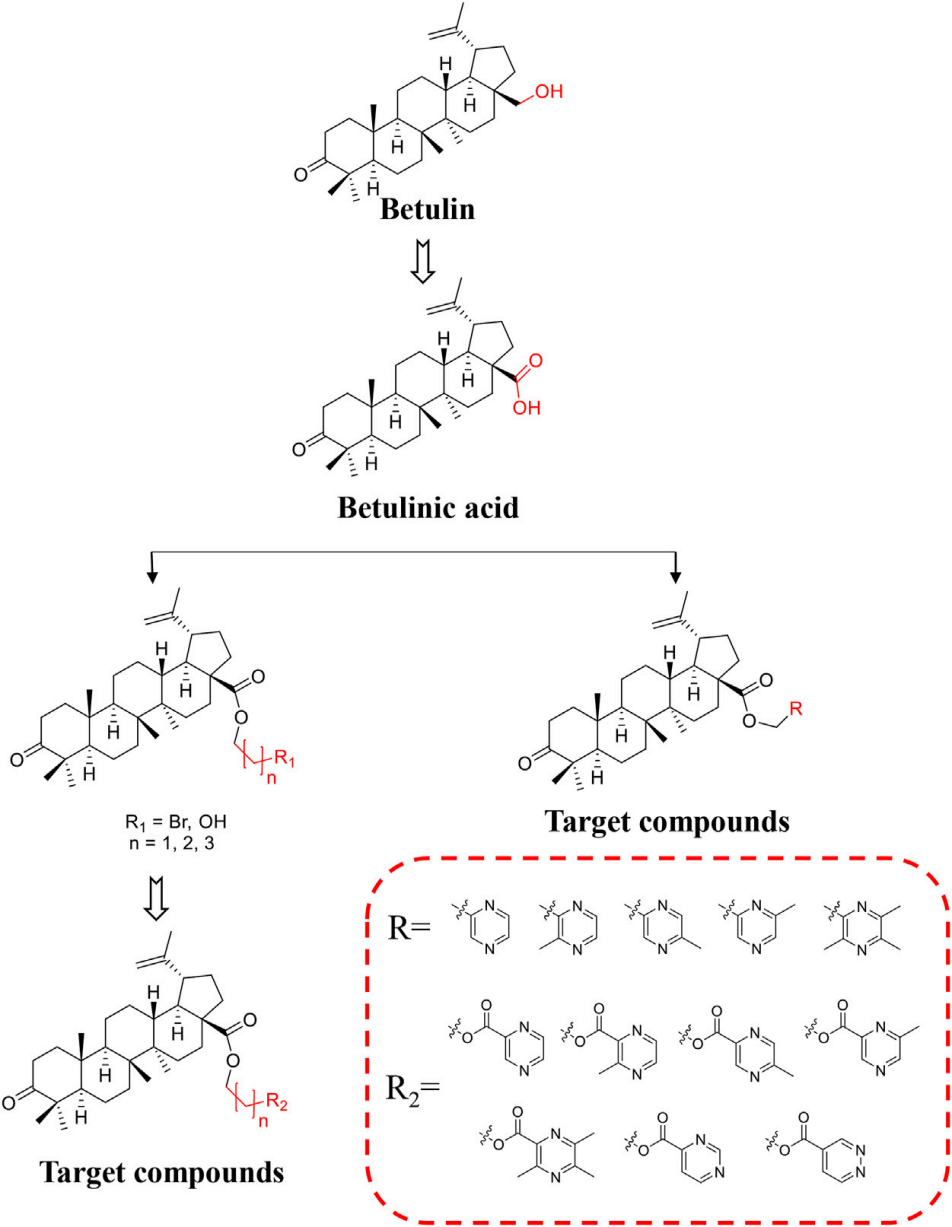
Illustration of the design strategy for target compounds.

BoA has two major functional groups, 3-=O and 28-COOH, which are susceptible to functionalization by transformations such as amination, esterification, sulfonation, hydroxylation, and alkylation. And many of these derivatives (Figure 1) are known to possess various types of biological activities (Saxena et al., 2006; Dangroo et al., 2017; Eignerova et al., 2017; Gupta et al., 2017; Khlebnicova et al., 2017; Mzhelskaya et al., 2017; Khlebnicova et al., 2019; Popov et al., 2019; Sousa et al., 2019; Hoenke et al., 2020; Yang et al., 2020). A previous study demonstrated that BoA derivatives with fused azole and triazine displayed impressive cytotoxic activity against several cancer cell lines (Dinh Ngoc et al., 2014; Grishko et al., 2017). And BoA derivatives of nitrogen-containing heterocycles at the C-28 position exhibited excellent anticancer activities (Yang et al., 2015). There are also studies that boc-lysinated-betulonic acid (100 μM) had been shown 95.7% inhibition of LNCaP prostate cancer cells whereas little effect on normally proliferating fibroblast cells (Saxena et al., 2006). Besides, a series of C-28 derived 1,2,3-triazolyl derivatives of BoA possessed promising cytotoxicity activity (IC_50_ value in the range of 4–6 μM) (Dangroo et al., 2017). The preceding investigations piqued our curiosity in whether BoA with nitrogen-containing heterocycles at C-28 might be beneficial for anticancer activities. In this study, we successfully synthesized 26 new BoA derivatives by introducing pyrazines including TMP, pyrimidines and pyridazines at the C-28 (Figure 2), in order to improve its anticancer activity. The cytotoxicity of all these derivatives (**BoAA-BoAE, BoAnA-BoAnGn** (*n* = 2, 3, 4)) was evaluated against four human cancer cell lines (including HepG2, Bel-7402, HeLa, MCF-7) and normal cell line Figure 2 (MDCK). And the ability of the most potent active compound to trigger cell injury was preliminary analyzed *in vitro*.

## 2 Materials and methods

### 2.1 General aspects

Chemical shifts (*δ*) are given in ppm and coupling constants (*J*) in Hz. Reagents were bought from commercial suppliers without any further purification. NMR spectra were recorded on a Bruker-500 or -400 spectrometer (Bruker, Dresden, Germany) with tetramethylsilane (TMS; TCI, Tokyo, Japan) as an internal standard; high-resolution mass spectra (HRMS) were acquired using a Thermo Scientific TMLTQ Orbitrap XL hybrid FTMS instrument (Thermo Technologies, New York, NY, United States); Melting points were measured at a rate of 5°C/min using an X-5 micro melting point apparatus (Beijing, China) and were not corrected.

### 2.1.1 Preparation of BoA

Betulin (9.0 g, 61.02 mmol) was fully dispersed in acetone (800 ml). Neutral aluminum oxide (54 g) and K_2_Cr_2_O_7_ (18 g, 60 mmol) were added to a solution of H_2_SO_4_ (30 ml) in H_2_O (120 ml), which was added in batches into the Betulin suspensions after cooling to room temperature. The mixture was stirred for 3 h at 25°C. After completion of the reaction (as monitored by TLC), the mixture was filtered, evaporated, diluted with water (200 ml), extracted with ethyl acetate (3 × 100 ml) in sequence. Then the organic layer was dried over anhydrous sodium sulfate, filtrated and evaporated. The crude product was dissolved in tert-Butyl methyl ether (TBME) (100 ml), which was dropped into the 5% NaOH aqueous solution (200 ml), a lot of white precipitate was produced. After filtering, washing with TBME (3 × 10 ml), the BoA sodium salt was obtained. The solid was put in 3% hydrochloric acid (100 ml), extracted with ethyl acetate (3 × 50 ml), dried over anhydrous sodium sulfate, filtrated and evaporated. The precipitate was recrystallized from methanol. White flake crystal, 82.22% yield. m. p. 250–252°C. ^1^H NMR (500 MHz, CDCl_3_) *δ* (ppm) 1.38 (m, 1H, H-1a), 1.89 (m, 1H, H-1b), 2.41 (m, 1H, H-2a), 2.47 (m, 1H, H-2b), 1.32 (m, 1H, H-5), 1.44 (m, 1H, H-6a), 1.47 (m, 1H, H-6b), 1.42 (m, 2H, H_2_-7), 1.38 (o, 1H, H-9), 1.30 (m, 1H, H-11a), 1.46 (o, 1H, H-11b), 1.05 (m, 1H, H-12a), 1.72 (m, 1H, H-12b), 2.21 (td, *J* = 12.5, 2.5 Hz, 1H, H-13), 1.22 (br dt, *J* = 13.5, 2.5 Hz, 1H, H-15a), 1.54 (m, 1H, H-15b), 1.42 (o, 1H, H-16a), 2.28 (dt, *J* = 13.0, 3.0 Hz, 1H, H-16b), 1.63 (t, *J* = 11.5 Hz, 1H, H-18), 3.01 (td, *J* = 10.5, 5.0 Hz, 1H, H-19), 1.43 (o, 1H, H-21a), 2.00 (m, 1H, H-21b), 1.48 (o, 1H, H-22a), 1.98 (m, 1H, H-22b), 1.07 (s, 3H, CH_3_-23), 1.01 (s, 3H, CH_3_-24), 0.92 (s, 3H, CH_3_-25), 0.97 (s, 3H, CH_3_-26), 0.99 (s, 3H, CH_3_-27), 4.61 (brs, 1H, H-29*E*), 4.74 (d, *J* = 0.8 Hz, 1H, H-29*Z*), 1.69 (s, 3H, CH_3_-30). ^13^C NMR (125 MHz, CDCl_3_) *δ* (ppm) 39.7 (C-1), 34.3 (C-2), 218.3 (C-3), 47.5 (C-4), 55.1 (C-5), 19.8 (C-6), 33.7 (C-7), 40.8 (C-8), 50.0 (C-9), 37.1 (C-10), 21.5 (C-11), 25.6 (C-12), 38.7 (C-13), 42.6 (C-14), 29.8 (C-15), 32.2 (C-16), 56.5 (C-17), 49.3 (C-18), 47.0 (C-19), 150.4 (C-20), 30.7 (C-21), 37.2 (C-22), 26.8 (C-23), 21.1 (C-24), 16.1 (C-25), 16.0 (C-26), 14.8 (C-27), 182.3 (C-28), 109.9 (C-29), 19.5 (C-30). The above data were identical with the literature data (Ayers et al., 2016). HRMS (ESI) *m/z*: [M + H]^+^ 455.3523, calcd. for C_30_H_47_O_3_ 455.3525.

#### 2.1.2 Preparation of BoAnBr/BoAnOH (*n* = 2, 3, 4)

BoA (1.2 g, 2.64 mmoL) was dissolved in 20 ml dry DMF, then 1, 2-dibromoethane/1, 3-dibromopropane/1, 4-dibromobutane (13.20 mmol) and K_2_CO_3_ (5.28 mmoL) was added successively, and the mixture was stirred for 4 h at 25°C. Then the reaction mixture was diluted with water and then extracted with ethyl acetate. The combined organic layer was dried over anhydrous sodium sulfate and evaporated the solvent under vacuum. The crude product was purified by flash chromatography (silica gel, petroleum ether: acetone = 50:1). Then **BoA2Br**, **BoA3Br**, **BoA4Br** was obtained respectively.

When 1, 2-dibromoethane/1, 3-dibromopropane/1, 4-dibromobutane (13.20 mmol) was replaced with 2-bromoethanol/3-bromo-1-propanol (3.43 mmol) and other conditions were not changed, **BoA2OH**, **BoA3OH** was obtained separately.

When BoA (1.2 g, 2.64 mmol), 1, 2-dibromoethane/1, 3-dibromopropane/1, 4-dibromobutane (13.20 mmol) and K_2_CO_3_ (5.28 mmol) were replaced with BoA (2.0 g, 4.40 mmol), 4-chloro-1-butanol (5.72 mmol) and K_2_CO_3_ (13.2 mmol) and other conditions were not changed, **BoA4OH** was obtained.

#### 2.1.3 General procedure for the synthesis of BoAA-BoAB

BoA (200 mg, 0.44 mmol) was dissolved in 10 ml dry DMF, then chloromethyl pyrazine (about 0.44 mmol) and K_2_CO_3_ (1.32 mmol) was added in sequence, the mixture was stirred for 2 h at room temperature. Then the reaction mixture was poured into water (50 ml) and the crude product was extracted with ethyl acetate (20 ml). The organic layer was washed with brine (20 ml), dried over sodium sulfate, filtrated and evaporated. Purification was performed by flash chromatography (silica gel, dichloromethane: methanol = 40:1).

##### 2.1.3.1*Pyrazin-2-ylmethyl-3-oxolup-20(29)-en-28-oate* (BoAA)

**BoAA** was obtained as white powder; Yield: 49.89%; m. p. 79–81°C. ^1^H NMR (500 MHz, CDCl_3_) *δ* 8.68 (s, 1H, H-3′), 8.56 (t, *J* = 2.0 Hz, 1H, H-5′), 8.54 (d, *J* = 2.0 Hz, 1H, H-6′), 5.26 (m, 2H, CH_2_-2′), 4.60 (brs, 1H, H-29*E*), 4.72 (d, *J* = 2.0 Hz, 1H, H-29*Z*), 3.00 (td, *J* = 11.0, 5.0 Hz, 1H, H-19), 2.39 (m, 1H, H-2a), 2.47 (m, 1H, H-2b), 2.32 (dt, *J* = 13.0, 3.0 Hz, 1H, H-16b), 2.21 (td, *J* = 12.5, 3.5 Hz, 1H, H-13), 1.68, 1.06, 1.01, 0.96, 0.90, 0.83 (s, each 3H, 6 × −CH_3_). ^13^C NMR (125 MHz, CDCl_3_) *δ* 218.3, 175.6, 152.1, 150.4, 144.3, 144.1, 144.0, 109.9, 64.5, 56.8, 55.1, 50.0, 49.5, 47.5, 46.9, 42.6, 40.7, 39.7, 38.4, 37.0 ✕ 2, 34.3, 33.7, 32.1, 30.6, 29.8, 26.7, 25.6, 21.5, 21.2, 19.7, 19.5, 16.1, 15.8, 14.7. HRMS (ESI) *m/z*: [M + H]^+^ 547.3905, calculated for C_35_H_51_N_2_O_3_ 547.3900.

##### 2.1.3.2 *(3-Methylpyrazin-2-yl)-methyl-3-oxolup-20(29)-en-28-oate* (BoAB)

**BoAB** was obtained as white powder; Yield: 61.62%; m. p. 80–82°C. ^1^H NMR (400 MHz, CDCl_3_) *δ* 8.42 (s, 1H, H-5′), 8.38 (s, 1H, H-6′), 2.63 (s, 3H, CH_3_-3′), 5.25 (m, 2H, CH_2_-2′), 4.59 (s, 1H, H-29*E*), 4.71 (s, 1H, H-29*Z*), 3.00 (m, 1H, H-19), 2.38 (m, 1H, H-2a), 2.46 (m, 1H, H-2b), 2.27 (m, 1H, H-16b), 2.22 (m, 1H, H-13), 1.67, 1.06, 1.01, 0.95, 0.90, 0.84 (s, each 3H, 6 × −CH_3_). ^13^C NMR (100 MHz, CDCl_3_) *δ* 218.2, 175.6, 153.0, 150.5, 149.8, 143.4, 141.7, 109.8, 64.4, 56.8, 55.1, 50.0, 49.6, 47.5, 46.9, 42.6, 40.7, 39.8, 38.4, 37.1, 37.0, 34.3, 33.8, 32.1, 30.6, 29.7, 26.8, 25.6, 21.5, 21.3, 21.2, 19.8, 19.5, 16.1, 15.9, 14.7. HRMS (ESI) *m/z*: [M + H]^+^ 561.4055, calculated for C_36_H_53_N_2_O_3_ 561.4056.

#### 2.1.4 General procedure for the synthesis of BoAC-BoAD

To a solution of dichlorosulfoxide (106 mg, 0.88 mmol) in dichloromethane (20 ml), methyl-2-pyrazinylmethanol (110 mg, 0.88 mmol) was added. The reaction mixture was allowed to stir for 2 h at room temperature. After completion of the reaction (as monitored by TLC), the solution was evaporated. Then BoA (200 mg, 0.44 mmoL), dry DMF (10 ml), K_2_CO_3_ (1.32 mmol) was added in sequence, and the mixture was stirred for 4 h at room temperature. The reaction mixture was diluted with water (50 ml) and then extracted with ethyl acetate (20 ml). the organic layer was washed with brine (20 ml), dried over sodium sulfate, filtrated and evaporated. Purification was performed by flash chromatography (silica gel, dichloromethane: methanol = 40:1).

##### 2.1.4.1 *(5-Methylpyrazin-2-yl)-methyl-3-oxolup-20(29)-en-28-oate* (BoAC)

**BoAC** was obtained as white powder; Yield: 52.70%; m. p. 125–127°C. ^1^H NMR (500 MHz, CDCl_3_) *δ* 8.54 (s, 1H, H-3′), 8.43 (s, 1H, H-6′), 2.58 (s, 3H, CH_3_-5′), 5.22 (m, 2H, CH_2_-2′), 4.59 (s, 1H, H-29*E*), 4.72 (s, 1H, H-29*Z*), 3.00 (td, *J* = 11.0, 5.0 Hz, 1H, H-19), 2.38 (m, 1H, H-2a), 2.47 (m, 1H, H-2b), 2.30 (m, 1H, H-16b), 2.18 (m, 1H, H-13), 1.67, 1.06, 1.01, 0.96, 0.90, 0.83 (s, each 3H, 6 × −CH_3_). ^13^C NMR (125 MHz, CDCl_3_) *δ* 218.3, 175.7, 153.3, 150.4, 148.7, 144.0, 142.8, 109.9, 64.4, 56.8, 55.1, 50.0, 49.5, 47.5, 46.9, 42.6, 40.7, 39.8, 38.4, 37.0 ✕ 2, 34.3, 33.7, 32.1, 30.6, 29.8, 26.7, 25.6, 21.52, 21.5 ✕ 2, 21.2, 19.7, 19.5, 16.1, 15.8, 14.7. HRMS (ESI) *m/z*: [M + H]^+^ 561.4059, calculated for C_36_H_53_N_2_O_3_ 561.4056.

##### 2.1.4.2 *(6-Methylpyrazin-2-yl)-methyl-3-oxolup-20(29)-en-28-oate* (BoAD)

**BoAD** was obtained as white powder; Yield: 40.54%; m. p. 119–121°C. ^1^H NMR (500 MHz, CDCl_3_) *δ* 8.46 (s, 1H, H-3′), 8.40 (s, 1H, H-5′), 2.56 (s, 3H, CH_3_-6′), 5.22 (m, 2H, CH_2_-2′), 4.59 (brs, 1H, H-29*E*), 4.72 (d, *J* = 1.5 Hz, 1H, H-29*Z*), 3.00 (td, *J* = 11.5, 5.0 Hz, 1H, H-19), 2.38 (m, 1H, H-2a), 2.47 (m, 1H, H-2b), 2.32 (dt, *J* = 13.0, 3.0 Hz, 1H, H-16b), 2.21 (td, *J* = 11.5, 2.5 Hz, 1H, H-13), 1.67, 1.05, 1.00, 0.96, 0.89, 0.82 (s, each 3H, 6 × −CH_3_). ^13^C NMR (125 MHz, CDCl_3_) *δ* 218.3, 175.6, 153.4, 150.8, 150.4, 143.8, 140.5, 109.9, 64.5, 56.8, 55.1, 50.0, 49.5, 47.5, 46.9, 42.6, 40.7, 39.7, 38.4, 37.0 × 2, 34.3, 33.7, 32.1, 30.6, 29.8, 26.7, 25.6, 21.6, 21.5, 21.2, 19.7, 19.5, 16.1, 15.8, 14.7. HRMS (ESI) *m/z*: [M + H]^+^ 561.4056, calculated for C_36_H_53_N_2_O_3_ 561.4056.

#### 2.1.5 General procedure for the synthesis of BoAE, BoAnB-BoAnE (*n* = 2,3,4)

**BoA**/**BoAnBr (*n* = 2, 3, 4)** (about 0.27 mmol, prepared as detailed in Section 4.1.2) was dissolved in 10 ml dry DMF, then chloroligustrazine/methyl-2-pyrazinic acid (about 0.27 mmol), K_2_CO_3_ (about 0.8 mmol) was added respectively, and the mixture was stirred for 4 h at 85°C. The reaction mixture was poured into water (50 ml) and then extracted with ethyl acetate (20 ml). The combined organic extracts were washed with brine (20 ml), dried over sodium sulfate, filtrated and evaporated. Then the crude product was purified by flash chromatography (silica gel, dichloromethane: methanol = 40:1).

##### 2.1.5.1 *(3,5,6-Trimethylpyrazin-2-yl)-methyl-3-oxolup-20(29)-en-28-oate* (BoAE)

**BoAE** was obtained as white powder; Yield: 82.62%; m. p. 177–179°C. ^1^H NMR (500 MHz, CDCl_3_) *δ* 2.54 (s, 3H, CH_3_-3′), 2.51 (s, 3H, CH_3_-5′), 2.49 (s, 3H, CH_3_-6′), 5.18 (m, 2H, CH_2_-2′), 4.58 (brs, 1H, H-29*E*), 4.71 (d, *J* = 2.0 Hz, 1H, H-29*Z*), 2.99 (td, *J* = 11.5, 5.0 Hz, 1H, H-19), 2.38 (m, 1H, H-2a), 2.46 (m, 1H, H-2b), 2.27 (m, 1H, H-16b), 2.22 (m, 1H, H-13), 1.66, 1.05, 1.01, 0.94, 0.90, 0.82 (s, each 3H, 6 × -CH_3_). ^13^C NMR (125 MHz, CDCl_3_) *δ* 218.3, 175.6, 151.0, 150.6, 149.1, 148.8, 145.5, 109.8, 64.5, 56.8, 55.1, 50.0, 49.5, 47.5, 46.9, 42.6, 40.7, 39.7, 38.3, 37.0, 34.3, 33.7, 32.1, 30.6, 29.7, 26.7, 25.6, 21.8, 21.5 × 2, 21.2, 20.6, 19.7, 19.5, 16.1, 15.8, 14.7. HRMS (ESI) *m/z*: [M + H]^+^ 589.4370, calculated for C_38_H_57_N_2_O_3_ 589.4369.

##### 2.1.5.2 *(3-Methylpyrazin-2-acyloxy)-ethyl-3-oxolup-20(29)-en-28-oate* (BoA2B)

**BoA2B** was obtained as white powder; Yield: 74.42%; m. p. 60–62°C. ^1^H NMR (500 MHz, CDCl_3_) *δ* 8.62 (d, *J* = 2.0 Hz, 1H, H-5″), 8.52 (brs, 1H, H-6″), 2.85 (s, 3H, CH_3_-3″), 4.67, 4.62 (m, each 1H, CH_2_-1′), 4.47 (m, 2H, CH_2_-2′), 4.57 (s, 1H, H-29*E*), 4.68 (s, 1H, H-29*Z*), 2.95 (m, 1H, H-19), 2.38 (m, 1H, H-2a), 2.47 (m, 1H, H-2b), 2.24 (m, 1H, H-16b), 2.18 (td, *J* = 12.5, 3.5 Hz, 1H, H-13), 1.65, 1.05, 1.01, 0.93, 0.87, 0.84 (s, each 3H, 6 × -CH_3_). ^13^C NMR (125 MHz, CDCl_3_) *δ* 218.3, 175.9, 165.1, 155.5, 150.4, 146.2, 142.9, 141.6, 109.8, 63.8, 61.5, 56.7, 55.0, 49.9, 49.4, 47.5, 47.0, 42.5, 40.7, 39.7, 38.4, 37.0 × 2, 34.3, 33.6, 32.1, 30.6, 29.7, 26.7, 25.6, 23.4, 21.4, 21.2, 19.7, 19.5, 16.1, 15.8, 14.7. HRMS (ESI) *m/z*: [M + H]^+^ 619.4117, calculated for C_38_H_55_N_2_O_5_ 619.4111.

##### 2.1.5.3 *(5-Methylpyrazin-2-acyloxy)-ethyl-3-oxolup-20(29)-en-28-oate* (BoA2C)

**BoA2C** was obtained as white powder; Yield: 63.53%; m. p. 139–141°C. ^1^H NMR (500 MHz, CDCl_3_) *δ* 9.18 (s, 1H, H-3″), 8.59 (s, 1H, H-6″), 2.67 (s, 3H, CH_3_-5″), 4.72, 4.63 (m, each 1H, CH_2_-1′), 4.50, 4.43 (m, each 1H, CH_2_-2′), 4.57 (s, 1H, H-29*E*), 4.68 (d, *J* = 2.0 Hz, 1H, H-29*Z*), 2.96 (td, *J* = 11.0, 5.0 Hz, 1H, H-19), 2.38 (m, 1H, H-2a), 2.46 (m, 1H, H-2b), 2.24 (m, 1H, H-16b), 2.17 (td, *J* = 12.0, 3.0 Hz, 1H, H-13), 1.65, 1.05, 1.00, 0.93, 0.86, 0.84 (s, each 3H, 6 × -CH_3_). ^13^C NMR (125 MHz, CDCl_3_) *δ* 218.3, 175.9, 164.0, 158.2, 150.4, 145.5, 144.6, 140.4, 109.9, 63.7, 61.5, 56.7, 55.0, 49.9, 49.4, 47.4, 47.0, 42.5, 40.6, 39.7, 38.5, 37.0 × 2, 34.3, 33.6, 32.1, 30.6, 29.7, 26.7, 25.6, 22.2, 21.4, 21.2, 19.7, 19.5, 16.0, 15.8, 14.7. HRMS (ESI) *m/z*: [M + H]^+^ 619.4115, calculated for C_38_H_55_N_2_O_5_ 619.4111.

##### 2.1.5.4 *(6-Methylpyrazin-2-acyloxy)-ethyl-3-oxolup-20(29)-en-28-oate* (BoA2D)

**BoA2D** was obtained as white powder; Yield: 68.97%; m. p. 139–141°C. ^1^H NMR (500 MHz, CDCl_3_) *δ* 9.10 (s, 1H, H-3″), 8.64 (s, 1H, H-5″), 2.68 (s, 3H, CH_3_-6″), 4.72, 4.63 (m, each 1H, CH_2_-1′), 4.47 (m, 2H, CH_2_-2′), 4.57 (brs, 1H, H-29*E*), 4.68 (brs, 1H, H-29*Z*), 2.96 (td, *J* = 11.0, 4.5 Hz, 1H, H-19), 2.38 (m, 1H, H-2a), 2.47 (m, 1H, H-2b), 2.25 (m, 1H, H-16b), 2.17 (td, *J* = 11.5, 3.5 Hz, 1H, H-13), 1.65, 1.05, 1.00, 0.93, 0.86, 0.85 (s, each 3H, 6 × −CH_3_). ^13^C NMR (125 MHz, CDCl_3_) *δ* 218.3, 175.9, 164.0, 154.5, 150.4, 148.0, 143.3, 142.2, 109.9, 63.8, 61.5, 56.7, 55.0, 49.9, 49.4, 47.4, 47.0, 42.5, 40.7, 39.7, 38.5, 37.1, 37.0, 34.3, 33.6, 32.1, 30.6, 29.7, 26.7, 25.6, 21.8, 21.4, 21.2, 19.7, 19.4, 16.0, 15.8, 14.7. HRMS (ESI) *m/z*: [M + H]^+^ 619.4112, calculated for C_38_H_55_N_2_O_5_ 619.4111.

##### 2.1.5.5 *(3,5,6-Trimethylpyrazin-2-acyloxy)-ethyl-3-oxolup-20(29)-en-28-oate* (BoA2E)

**BoA2E** was obtained as white powder; Yield: 70.03%; m. p. 74–76°C. ^1^H NMR (500 MHz, CDCl_3_) *δ* 2.75 (s, 3H, CH_3_-3″), 2.56 (s, 3H, CH_3_-5″), 2.55 (s, 3H, CH_3_-6″), 4.65, 4.60 (m, each 1H, CH_2_-1′), 4.48, 4.42 (m, each 1H, CH_2_-2′), 4.57 (brs, 1H, H-29*E*), 4.67 (d, *J* = 1.5 Hz, 1H, H-29*Z*), 2.96 (td, *J* = 11.0, 5.0 Hz, 1H, H-19), 2.38 (m, 1H, H-2a), 2.46 (m, 1H, H-2b), 2.25 (m, 1H, H-16b), 2.17 (td, *J* = 12.5, 3.5 Hz, 1H, H-13), 1.65, 1.05, 1.00, 0.93, 0.86, 0.82 (s, each 3H, 6 × -CH_3_). ^13^C NMR (125 MHz, CDCl_3_) *δ* 218.3, 175.9, 165.8, 154.8, 151.5, 150.5, 149.5, 139.3, 109.8, 63.5, 61.6, 56.7, 55.0, 49.9, 49.4, 47.4, 47.0, 42.5, 40.6, 39.7, 38.4, 37.0 × 2, 34.3, 33.6, 32.1, 30.6, 29.7, 26.7, 25.6, 22.8, 22.4, 21.7, 21.4, 21.2, 19.7, 19.5, 16.0, 15.7, 14.7. HRMS (ESI) *m/z*: [M + H]^+^ 647.4428, calculated for C_40_H_59_N_2_O_5_ 647.4424.

##### 2.1.5.6 *(3-Methylpyrazin-2-acyloxy)-propyl-3-oxolup-20(29)-en-28-oate* (BoA3B)

**BoA3B** was obtained as white oil; Yield: 60.64%; m. p. 54–55°C. ^1^H NMR (500 MHz, CDCl_3_) *δ* 8.62 (d, *J* = 2.5 Hz, 1H, H-5″), 8.53 (d, *J* = 2.0 Hz, 1H, H-6″), 2.85 (s, 3H, CH_3_-3″), 4.24 (m, 2H, CH_2_-1′), 2.20 (m, 2H, CH_2_-2′), 4.52 (t, *J* = 6.5 Hz, 2H, CH_2_-3′), 4.60 (brs, 1H, H-29*E*), 4.73 (d, *J* = 1.5 Hz, 1H, H-29*Z*), 2.99 (td, *J* = 11.5, 5.0 Hz, 1H, H-19), 2.38 (m, 1H, H-2a), 2.48 (m, 1H, H-2b), 2.26 (m, 1H, H-16b), 2.17 (m, 1H, H-13), 1.68, 1.06, 1.01, 0.96, 0.91, 0.90 (s, each 3H, 6 × -CH_3_). ^13^C NMR (125 MHz, CDCl_3_) *δ* 218.3, 176.1, 165.4, 155.4, 150.5, 146.1, 143.1, 141.6, 109.9, 63.0, 60.5, 56.7, 55.1, 50.0, 49.5, 47.5, 47.1, 42.6, 40.8, 39.8, 38.5, 37.1, 37.0, 34.3, 33.7, 32.2, 30.7, 29.8, 28.3, 26.8, 25.7, 23.4, 21.6, 21.2, 19.8, 19.5, 16.1, 15.9, 14.8. HRMS (ESI) *m/z*: [M + H]^+^ 633.4268, calculated for C_39_H_57_N_2_O_5_ 633.4267.

##### 2.1.5.7 *(5-Methylpyrazin-2-acyloxy)-propyl-3-oxolup-20(29)-en-28-oate* (BoA3C)

**BoA3C** was obtained as white powder; Yield: 67.92%; m. p. 57–58°C. ^1^H NMR (400 MHz, CDCl_3_) *δ* 9.18 (s, 1H, H-3″), 8.59 (s, 1H, H-6″), 2.67 (s, 3H, CH_3_-5″), 4.24 (m, 2H, CH_2_-1′), 2.20 (o, 2H, CH_2_-2′), 4.53 (t, *J* = 7.5 Hz, 2H, CH_2_-3′), 4.59 (s, 1H, H-29*E*), 4.72 (s, 1H, H-29*Z*), 2.99 (m, 1H, H-19), 2.39 (m, 1H, H-2a), 2.47 (m, 1H, H-2b), 2.24 (m, 1H, H-16b), 2.17 (m, 1H, H-13), 1.67, 1.05, 1.00, 0.96, 0.88 × 2 (s, each 3H, 6 × -CH_3_). ^13^C NMR (100 MHz, CDCl_3_) *δ* 218.2, 176.0, 164.2, 158.0, 150.5, 145.5, 144.5, 140.6, 109.9, 62.9, 60.4, 56.7, 55.1, 50.0, 49.5, 47.4, 47.1, 42.6, 40.7, 39.7, 38.5, 37.1, 37.0, 34.3, 33.7, 32.2, 30.7, 29.8, 28.2, 26.8, 25.6, 22.1, 21.5, 21.1, 19.8, 19.5, 16.1, 15.9, 14.7. HRMS (ESI) *m/z*: [M + H]^+^ 633.4269, calculated for C_39_H_57_N_2_O_5_ 633.4267.

##### 2.1.5.8 *(6-Methylpyrazin-2-acyloxy)-propyl-3-oxolup-20(29)-en-28-oate* (BoA3D)

**BoA3D** was obtained as white powder; Yield: 76.41%; m. p. 59–60°C. ^1^H NMR (500 MHz, CDCl_3_) *δ* 9.10 (s, 1H, H-3″), 8.64 (s, 1H, H-5″), 2.68 (s, 3H, CH_3_-6″), 4.24 (m, 2H, CH_2_-1′), 2.20 (o, 2H, CH_2_-2′), 4.54 (t, *J* = 6.5 Hz, 2H, CH_2_-3′), 4.60 (s, 1H, H-29*E*), 4.73 (s, 1H, H-29*Z*), 2.99 (td, *J* = 11.0, 4.5 Hz, 1H, H-19), 2.39 (m, 1H, H-2a), 2.48 (m, 1H, H-2b), 2.24 (m, 1H, H-16b), 2.17 (m, 1H, H-13), 1.67, 1.06, 1.00, 0.96, 0.89 × 2 (s, each 3H, 6 × −CH_3_). ^13^C NMR (125 MHz, CDCl_3_) *δ* 218.3, 176.1, 164.2, 154.4, 150.5, 147.9, 143.3, 142.4, 109.9, 63.0, 60.4, 56.7, 55.0, 50.0, 49.4, 47.5, 47.1, 42.6, 40.7, 39.7, 38.5, 37.1, 37.0, 34.3, 33.7, 32.2, 30.7, 29.7, 28.2, 26.7, 25.6, 21.9, 21.5, 21.2, 19.7, 19.5, 16.1, 15.9, 14.7. HRMS (ESI) *m/z*: [M + H]^+^ 633.4269, calculated for C_39_H_57_N_2_O_5_ 633.4267.

##### 2.1.5.9 *(3,5,6-Trimethylpyrazin-2-acyloxy)-propyl-3-oxolup-20(29)-en-28-oate* (BoA3E)

**BoA3E** was obtained as white powder; Yield: 75.49%; m. p. 57–59°C. ^1^H NMR (500 MHz, CDCl_3_) *δ* 2.74 (s, 3H, CH_3_-3″), 2.56 (s, 6H, CH_3_-5″, 6″), 4.23 (m, 2H, CH_2_-1′), 2.19 (o, 2H, CH_2_-2′), 4.48 (t, *J* = 6.5 Hz, 2H, CH_2_-3′), 4.59 (brs, 1H, H-29*E*), 4.72 (d, *J* = 2.0 Hz, 1H, H-29*Z*), 2.99 (td, *J* = 11.0, 5.0 Hz, 1H, H-19), 2.38 (m, 1H, H-2a), 2.47 (m, 1H, H-2b), 2.24 (m, 1H, H-16b), 2.15 (m, 1H, H-13), 1.67, 1.05, 1.00, 0.95, 0.88 × 2 (s, each 3H, 6 × -CH_3_). ^13^C NMR (125 MHz, CDCl_3_) *δ* 218.3, 176.1, 166.0, 154.7, 151.3, 150.5, 149.5, 139.5, 109.8, 62.6, 60.6, 56.7, 55.0, 50.0, 49.4, 47.5, 47.0, 42.6, 40.7, 39.7, 38.4, 37.1, 37.0, 34.3, 33.7, 32.2, 30.7, 29.7, 28.2, 26.7, 25.6, 22.8, 22.3, 21.8, 21.5, 21.2, 19.7, 19.5, 16.1, 15.9, 14.7. HRMS (ESI) *m/z*: [M + H]^+^ 661.4582, calculated for C_41_H_61_N_2_O_5_ 661.4580.

##### 2.1.5.10 *(3-Methylpyrazin-2-acyloxy)-butyl-3-oxolup-20(29)-en-28-oate* (BoA4B)

**BoA4B** was obtained as colorless oil; Yield: 79.29%; m. p. 54–55°C. ^1^H NMR (500 MHz, CDCl_3_) *δ* 8.61 (d, *J* = 2.5 Hz, 1H, H-5″), 8.52 (d, *J* = 2.5 Hz, 1H, H-6″), 2.85 (s, 3H, CH_3_-3″), 4.14 (m, 2H, CH_2_-1′), 1.59 (m, 2H, CH_2_-2′), 1.80 (o, 2H, CH_2_-3′), 4.46 (t, *J* = 6.5 Hz, 2H, CH_2_-4′), 4.59 (brs, 1H, H-29*E*), 4.72 (d, *J* = 2.0 Hz, 1H, H-29*Z*), 3.00 (td, *J* = 11.0, 5.0 Hz, 1H, H-19), 2.38 (m, 1H, H-2a), 2.48 (m, 1H, H-2b), 2.25 (m, 1H, H-16b), 2.21 (m, 1H, H-13), 1.67, 1.06, 1.01, 0.96, 0.92, 0.89 (s, each 3H, 6 × −CH_3_). ^13^C NMR (125 MHz, CDCl_3_) *δ* 218.4, 176.2, 165.4, 155.4, 150.6, 146.1, 143.2, 141.6, 109.8, 65.7, 63.4, 56.6, 55.1, 50.0, 49.4, 47.5, 47.1, 42.6, 40.8, 39.7, 38.4, 37.1, 37.0, 34.3, 33.7, 32.2, 30.7, 29.8, 26.7, 25.6 × 3, 23.4, 21.5, 21.2, 19.7, 19.5, 16.1, 15.9, 14.7. HRMS (ESI) *m/z*: [M + H]^+^ 647.4428, calculated for C_40_H_59_N_2_O_5_ 647.4424.

##### 2.1.5.11 *(5-Methylpyrazin-2-acyloxy)-butyl-3-oxolup-20(29)-en-28-oate* (BoA4C)

**BoA4C** was obtained as colorless oil; Yield: 76.57%; ^1^H NMR (500 MHz, CDCl_3_) *δ* 9.17 (d, *J* = 1.0 Hz, 1H, H-3″), 8.58 (s, 1H, H-6″), 2.66 (s, 3H, CH_3_-5″), 4.14 (m, 2H, CH_2_-1′), 1.70 (m, 2H, CH_2_-2′), 1.80 (m, 2H, CH_2_-3′), 4.47 (t, *J* = 6.5 Hz, 2H, CH_2_-4′), 4.58 (brs, 1H, H-29*E*), 4.71 (d, *J* = 2.0 Hz, 1H, H-29*Z*), 2.99 (td, 1H, *J* = 11.0, 4.5 Hz, H-19), 2.38 (m, 1H, H-2a), 2.47 (m, 1H, H-2b), 2.25 (m, 1H, H-16b), 2.21 (m, 1H, H-13), 1.67, 1.05, 1.00, 0.95, 0.92, 0.88 (s, each 3H, 6 × -CH_3_). ^13^C NMR (125 MHz, CDCl_3_) *δ* 218.3, 176.1, 164.3, 158.0, 150.6, 145.5, 144.5, 140.7, 109.8, 65.7, 63.4, 56.6, 55.0, 50.0, 49.4, 47.5, 47.1, 42.6, 40.7, 39.7, 38.4, 37.1, 37.0, 34.3, 33.7, 32.2, 30.7, 29.7, 26.7, 25.7, 25.6, 25.5, 22.1, 21.5, 21.2, 19.7, 19.5, 16.1, 15.9, 14.7. HRMS (ESI) *m/z*: [M + H]^+^ 647.4430, calculated for C_40_H_59_N_2_O_5_ 647.4424.

##### 2.1.5.12 *(6-Methylpyrazin-2-acyloxy)-butyl-3-oxolup-20(29)-en-28-oate* (BoA4D)

**BoA4D** was obtained as colorless oil; Yield: 79.00%; ^1^H NMR (500 MHz, CDCl_3_) *δ* 9.09 (s, 1H, H-3″), 8.63 (s, 1H, H-5″), 2.68 (s, 3H, CH_3_-6″), 4.14 (m, 2H, CH_2_-1′), 1.70 (m, 2H, CH_2_-2′), 1.80 (m, 2H, CH_2_-3′), 4.48 (t, *J* = 6.5 Hz, 2H, CH_2_-4′), 4.59 (brs, 1H, H-29*E*), 4.72 (d, *J* = 2.0 Hz, 1H, H-29*Z*), 2.99 (td, 1H, *J* = 11.0, 4.5 Hz, H-19), 2.38 (m, 1H, H-2a), 2.47 (m, 1H, H-2b), 2.25 (m, 1H, H-16b), 2.21 (m, 1H, H-13), 1.67, 1.05, 1.00, 0.96, 0.92, 0.88 (s, each 3H, 6 × −CH_3_). ^13^C NMR (125 MHz, CDCl_3_) *δ* 218.4, 176.1, 164.3, 154.3, 150.6, 147.9, 143.2, 142.5, 109.8, 65.8, 63.4, 56.6, 55.0, 50.0, 49.4, 47.4, 47.1, 42.6, 40.7, 39.7, 38.4, 37.1, 37.0, 34.3, 33.7, 32.2, 30.7, 29.7, 26.7, 25.6 × 2, 25.5, 21.9, 21.5, 21.1, 19.7, 19.5, 16.1, 15.9, 14.7. HRMS (ESI) *m/z*: [M + H]^+^ 647.4437, calculated for C_40_H_59_N_2_O_5_ 647.4424.

##### 2.1.5.13 *(3,5,6-Trimethylpyrazin-2-acyloxy)-butyl-3-oxolup-20(29)-en-28-oate* (BoA4E)

**BoA4E** was obtained as white powder; Yield: 73.39%; m. p. 124–126°C. ^1^H NMR (500 MHz, CDCl_3_) *δ* 2.73 (s, 3H, CH_3_-3″), 2.55 (s, 6H, CH_3_-5″, 6″), 4.13 (m, 2H, CH_2_-1′), 1.71 (m, 2H, CH_2_-2′), 1.78 (m, 2H, CH_2_-3′), 4.42 (t, *J* = 6.5 Hz, 2H, CH_2_-4′), 4.58 (brs, 1H, H-29*E*), 4.71 (d, *J* = 1.5 Hz, 1H, H-29*Z*), 2.98 (td, 1H, *J* = 11.0, 4.5 Hz, H-19), 2.37 (m, 1H, H-2a), 2.47 (m, 1H, H-2b), 2.24 (m, 1H, H-16b), 2.20 (m, 1H, H-13), 1.66, 1.04, 0.99, 0.95, 0.90, 0.88 (s, each 3H, 6 × -CH_3_). ^13^C NMR (125 MHz, CDCl_3_) *δ* 218.3, 176.1, 166.0, 154.5, 151.1, 150.5, 149.5, 139.7, 109.8, 65.4, 63.5, 56.6, 55.0, 50.0, 49.4, 47.4, 47.0, 42.5, 40.7, 39.7, 38.4, 37.1, 37.0, 34.3, 33.7, 32.2, 30.7, 29.7, 26.7, 25.6 × 3, 22.7, 22.3, 21.8, 21.5, 21.1, 19.7, 19.5, 16.1, 15.9, 14.7. HRMS (ESI) *m/z*: [M + H]^+^ 675.4734, calculated for C_42_H_63_N_2_O_5_ 675.4737.

#### 2.1.6 General procedure for the synthesis of BoAnA, BoAnF-BoAnG (*n* = 2,3,4)

**BoAnOH (*n* = 2, 3, 4)** (about 0.3 mmol, prepared as detailed in Section 4.1.2) was dissolved in 10 ml dichloromethane, then 2-pyrazinic acid/4-pyrimidinic acid/4-pyridazinic acid (about 0.3 mmol), EDCI (about 0.3 mmol), DMAP (0.04 mmol) was added in sequence, and the mixture was stirred for 4 h at room temperature. The reaction mixture was diluted with water (50 ml), then the solution was extracted with dichloromethane (20 ml), washed with brine (20 ml), dried over anhydrous sodium sulfate, filtrated and evaporated. Then the crude product was purified by flash chromatography (silica gel, dichloromethane: methanol = 40:1).

##### 2.1.6.1 *(Pyrazin-2-acyloxy)-ethyl-3-oxolup-20(29)-en-28-oate* (BoA2A)

**BoA2A** was obtained as white powder; Yield: 86.86%; m. p. 85–87°C. ^1^H NMR (500 MHz, CDCl_3_) *δ* 9.31 (d, *J* = 1.0 Hz, 1H, H-3″), 8.78 (d, *J* = 2.0 Hz, 1H, H-5″), 8.74 (t, *J* = 2.0 Hz, 1H, H-6″), 4.72, 4.66 (m, each 1H, CH_2_-1′), 4.48 (m, 2H, CH_2_-2′), 4.57 (s, 1H, H-29*E*), 4.68 (s, 1H, H-29*Z*), 2.96 (td, *J* = 11.0, 5.0 Hz, 1H, H-19), 2.38 (m, 1H, H-2a), 2.45 (m, 1H, H-2b), 2.25 (m, 1H, H-16b), 2.17 (td, *J* = 11.5, 3.5 Hz, 1H, H-13), 1.65, 1.05, 1.00, 0.93, 0.87, 0.86 (s, each 3H, 6 × −CH_3_). ^13^C NMR (125 MHz, CDCl_3_) *δ* 218.3, 175.9, 163.7, 150.4, 148.0, 146.4, 144.7, 143.2, 109.9, 64.0, 61.5, 56.7, 55.0, 49.9, 49.4, 47.5, 47.0, 42.5, 40.7, 39.7, 38.5, 37.1, 37.0, 34.3, 33.6, 32.1, 30.6, 29.7, 26.7, 25.6, 21.4, 21.2, 19.7, 19.5, 16.1, 15.8, 14.7. HRMS (ESI) *m/z*: [M + H]^+^ 605.3948, calculated for C_37_H_53_N_2_O_5_ 605.3954.

##### 2.1.6.2 *(Pyrimidin-2-acyloxy)-ethyl-3-oxolup-20(29)-en-28-oate* (BoA2F)

**BoA2F** was obtained as white powder; Yield: 79.00%; m. p. 72–74°C. ^1^H NMR (500 MHz, CDCl_3_) *δ* 9.41 (s, 1H, H-2″), 9.00 (d, *J* = 4.5 Hz, 1H, H-6″), 8.00 (d, *J* = 4.0 Hz, 1H, H-5″), 4.70, 4.63 (m, each 1H, CH_2_-1′), 4.47 (m, 2H, CH_2_-2′), 4.57 (s, 1H, H-29*E*), 4.67 (s, 1H, H-29*Z*), 2.94 (td, *J* = 11.0, 3.5 Hz, 1H, H-19), 2.37 (m, 1H, H-2a), 2.46 (m, 1H, H-2b), 2.23 (m, 1H, H-16b), 2.17 (td, *J* = 12.5, 2.5 Hz, 1H, H-13), 1.64, 1.04, 1.00, 0.93, 0.87, 0.86 (s, each 3H, 6 × −CH_3_). ^13^C NMR (125 MHz, CDCl_3_) *δ* 218.2, 175.9, 163.8, 159.5, 159.3, 154.5, 150.4, 121.1, 109.9, 64.3, 61.3, 56.7, 55.1, 49.9, 49.4, 47.4, 47.0, 42.5, 40.7, 39.7, 38.5, 37.0 × 2, 34.2, 33.6, 32.1, 30.6, 29.7, 26.7, 25.6, 21.4, 21.1, 19.7, 19.4, 16.0, 15.8, 14.7. HRMS (ESI) *m/z*: [M + H]^+^ 605.3952, calculated for C_37_H_53_N_2_O_5_ 605.3954.

##### 2.1.6.3 *(Pyridazin-2-acyloxy)-ethyl-3-oxolup-20(29)-en-28-oate* (BoA2G)

**BoA2G** was obtained as white powder; Yield: 72.00%; m. p. 130–132°C. ^1^H NMR (500 MHz, CDCl_3_) *δ* 9.67 (brs, 1H, H-3″), 9.45 (d, *J* = 5.0 Hz, 1H, H-6″), 7.99 (dd, *J* = 5.0, 2.0 Hz, 1H, H-5″), 4.67, 4.61 (m, each 1H, CH_2_-1′), 4.46 (m, 2H, CH_2_-2′), 4.59 (s, 1H, H-29*E*), 4.69 (s, 1H, H-29*Z*), 2.96 (td, *J* = 11.5, 4.5 Hz, 1H, H-19), 2.37 (m, 1H, H-2a), 2.47 (m, 1H, H-2b), 2.23 (dt, *J* = 12.5, 3.0 Hz, 1H, H-16b), 2.17 (td, *J* = 11.5, 3.5 Hz, 1H, H-13), 1.65, 1.05, 1.00, 0.94, 0.87, 0.86 (s, each 3H, 6 × −CH_3_). ^13^C NMR (125 MHz, CDCl_3_) *δ* 218.0, 175.9, 163.6, 152.2, 150.2, 149.7, 127.6, 125.6, 110.0, 64.4, 61.3, 56.8, 55.2, 50.0, 49.5, 47.5, 47.1, 42.6, 40.8, 39.8, 38.6, 37.1 × 2, 34.3, 33.8, 32.2, 30.7, 29.8, 26.8, 25.7, 21.5, 21.2, 19.7, 19.5, 16.0 × 2, 14.8. HRMS (ESI) *m/z*: [M + H]^+^ 605.3956, calculated for C_37_H_53_N_2_O_5_ 605.3954.

##### 2.1.6.4 *(Pyrazin-2-acyloxy)-propyl-3-oxolup-20(29)-en-28-oate* (BoA3A)

**BoA3A** was obtained as light yellow powder; Yield: 77.30%; m. p. 74–76°C. ^1^H NMR (500 MHz, CDCl_3_) *δ* 9.31 (s, 1H, H-3″), 8.78 (d, *J* = 2.5 Hz, 1H, H-5″), 8.74 (brs, 1H, H-6″), 4.25 (m, 2H, CH_2_-1′), 2.20 (o, 2H, CH_2_-2′), 4.54 (t, *J* = 6.5 Hz, 2H, CH_2_-3′), 4.59 (s, 1H, H-29*E*), 4.72 (s, 1H, H-29*Z*), 2.98 (m, 1H, H-19), 2.38 (m, 1H, H-2a), 2.45 (m, 1H, H-2b), 2.25 (m, 1H, H-16b), 2.19 (m, 1H, H-13), 1.67, 1.05, 1.00, 0.95, 0.88✕2 (s, each 3H, 6 × −CH_3_). ^13^C NMR (125 MHz, CDCl_3_) *δ* 218.3, 176.0, 163.9, 150.5, 147.9, 146.4, 144.6, 143.4, 109.9, 63.2, 60.4, 56.7, 55.0, 50.0, 49.4, 47.4, 47.0, 42.6, 40.7, 39.7, 38.4, 37.1, 37.0, 34.3, 33.7, 32.2, 30.7, 29.7, 28.2, 26.7, 25.6, 21.5, 21.2, 19.7, 19.5, 16.1, 15.9, 14.7. HRMS (ESI) *m/z*: [M + H]^+^ 619.4115, calculated for C_38_H_55_N_2_O_5_ 619.4111.

##### 2.1.6.5 *(Pyrimidin-2-acyloxy)-propyl-3-oxolup-20(29)-en-28-oate* (BoA3F)

**BoA3F** was obtained as white powder; Yield: 48.06%; m. p. 50–52°C. ^1^H NMR (500 MHz, CDCl_3_) *δ* 9.42 (d, *J* = 1.5 Hz, 1H, H-2″), 9.00 (d, *J* = 5.0 Hz, 1H, H-6″), 8.02 (dd, *J* = 5.0, 1.0 Hz, 1H, H-5″), 4.25 (m, 2H, CH_2_-1′), 2.20 (o, 2H, CH_2_-2′), 4.54 (t, *J* = 6.5 Hz, 2H, CH_2_-3′), 4.59 (brs, 1H, H-29*E*), 4.72 (d, *J* = 1.5 Hz, 1H, H-29*Z*), 2.98 (td, *J* = 11.5, 5.0 Hz, 1H, H-19), 2.38 (m, 1H, H-2a), 2.48 (m, 1H, H-2b), 2.24 (m, 1H, H-16b), 2.17 (m, 1H, H-13), 1.67, 1.05, 1.00, 0.95, 0.88 × 2 (s, each 3H, 6 × −CH_3_). ^13^C NMR (125 MHz, CDCl_3_) *δ* 218.0, 176.0, 164.1, 159.5, 159.3, 154.8, 150.5, 121.1, 109.9, 63.5, 60.4, 56.7, 55.2, 50.1, 49.5, 47.5, 47.1, 42.6, 40.8, 39.8, 38.5, 37.1 × 2, 34.3, 33.8, 32.2, 30.8, 29.8, 28.3, 26.8, 25.7, 21.6, 21.2, 19.8, 19.5, 16.1, 15.9, 14.8. HRMS (ESI) *m/z*: [M + H]^+^ 619.4107, calculated for C_38_H_55_N_2_O_5_ 619.4111.

##### 2.1.6.6 *(Pyridazin-2-acyloxy)-propyl-3-oxolup-20(29)-en-28-oate* (BoA3G)

**BoA3G** was obtained as light-yellow powder; Yield: 44.19%; m. p. 68–71°C. ^1^H NMR (500 MHz, CDCl_3_) *δ* 9.67 (d, *J* = 1.5 Hz, 1H, H-3″), 9.45 (dd, *J* = 5.0, 1.0 Hz, 1H, H-6″), 8.00 (dd, *J* = 5.0, 2.0 Hz, 1H, H-5″), 4.25 (m, 2H, CH_2_-1′), 2.19 (m, 2H, CH_2_-2′), 4.51 (t, *J* = 6.5 Hz, 2H, CH_2_-3′), 4.59 (brs, 1H, H-29*E*), 4.72 (d, *J* = 1.5 Hz, 1H, H-29*Z*), 2.98 (td, *J* = 11.0, 5.0 Hz, 1H, H-19), 2.38 (m, 1H, H-2a), 2.48 (m, 1H, H-2b), 2.23 (m, 1H, H-16b), 2.16 (m, 1H, H-13), 1.67, 1.05, 1.00, 0.96, 0.88 × 2 (s, each 3H, 6 × −CH_3_). ^13^C NMR (125 MHz, CDCl_3_) *δ* 218.0, 176.0, 163.8, 152.2, 150.4, 149.7, 127.8, 125.6, 109.9, 63.3, 60.2, 56.8, 55.2, 50.1, 49.6, 47.5, 47.1, 42.6, 40.8, 39.8, 38.6, 37.1 × 2, 34.3, 33.8, 32.2, 30.7, 29.8, 28.3, 26.8, 25.7, 21.6, 21.2, 19.8, 19.5, 16.1, 16.0, 14.8. HRMS (ESI) *m/z*: [M + H]^+^ 619.4113, calculated for C_38_H_55_N_2_O_5_ 619.4111.

##### 2.1.6.7 *(Pyrazin-2-acyloxy)-butyl-3-oxolup-20(29)-en-28-oate* (BoA4A)

**BoA4A** was obtained as colorless oil; Yield: 86.57%; m. p. 69–71°C. ^1^H NMR (500 MHz, CDCl_3_) *δ* 9.31 (s, 1H, H-3″), 8.77 (d, *J* = 2.5 Hz, 1H, H-5″), 8.74 (brs, 1H, H-6″), 4.14 (m, 2H, CH_2_-1′), 1.71 (m, 2H, CH_2_-2′), 1.81 (o, 2H, CH_2_-3′), 4.49 (t, *J* = 6.5 Hz, 2H, CH_2_-4′), 4.59 (s, 1H, H-29*E*), 4.72 (s, 1H, H-29*Z*), 2.99 (m, 1H, H-19), 2.38 (m, 1H, H-2a), 2.46 (m, 1H, H-2b), 2.25 (m, 1H, H-16b), 2.21 (m, 1H, H-13), 1.67, 1.05, 1.00, 0.96, 0.92, 0.89 (s, each 3H, 6 × −CH_3_). ^13^C NMR (125 MHz, CDCl_3_) *δ* 218.4, 176.1, 164.0, 150.6, 147.8, 146.4, 144.6, 143.5, 109.8, 65.9, 63.4, 56.6, 55.0, 50.0, 49.4, 47.5, 47.1, 42.6, 40.7, 39.7, 38.4, 37.1, 37.0, 34.3, 33.7, 32.2, 30.7, 29.7, 26.7, 25.6 × 2, 25.5, 21.5, 21.1, 19.7, 19.5, 16.1, 15.9, 14.7. HRMS (ESI) *m/z*: [M + H]^+^ 633.4271, calculated for C_39_H_57_N_2_O_5_ 633.4267.

##### 2.1.6.8 *(Pyrimidin-2-acyloxy)-butyl-3-oxolup-20(29)-en-28-oate* (BoA4F)

**BoA4F** was obtained as colorless oil; Yield: 77.69%; ^1^H NMR (500 MHz, CDCl_3_) *δ* 9.42 (s, 1H, H-2″), 9.00 (d, *J* = 4.0 Hz, 1H, H-6″), 8.02 (d, *J* = 3.5 Hz, 1H, H-5″), 4.14 (m, 2H, CH_2_-1′), 1.70 (m, 2H, CH_2_-2′), 1.80 (m, 2H, CH_2_-3′), 4.48 (t, *J* = 6.5 Hz, 2H, CH_2_-4′), 4.58 (brs, 1H, H-29*E*), 4.71 (d, *J* = 1.5 Hz, 1H, H-29*Z*), 2.99 (td, 1H, *J* = 11.0, 5.0 Hz, H-19), 2.38 (m, 1H, H-2a), 2.47 (m, 1H, H-2b), 2.25 (m, 1H, H-16b), 2.21 (m, 1H, H-13), 1.67, 1.05, 1.00, 0.95, 0.91, 0.87 (s, each 3H, 6 × −CH_3_). ^13^C NMR (125 MHz, CDCl_3_) *δ* 218.0, 176.1, 164.1, 159.5, 159.2, 154.9, 150.5, 121.1, 109.8, 66.3, 63.3, 56.7, 55.2, 50.1, 49.5, 47.5, 47.1, 42.6, 40.8, 39.8, 38.5, 37.1 × 2, 34.3, 33.8, 32.2, 30.8, 29.8, 26.8, 25.7, 25.6 × 2, 21.6, 21.2, 19.8, 19.5, 16.1, 16.0, 14.8. HRMS (ESI) *m/z*: [M + H]^+^ 633.4273, calculated for C_39_H_57_N_2_O_5_ 633.4267.

##### 2.1.6.9 *(Pyridazin-2-acyloxy)-butyl-3-oxolup-20(29)-en-28-oate* (BoA4G)

**BoA4G** was obtained as light yellow oil; Yield: 73.80%; ^1^H NMR (500 MHz, CDCl_3_) *δ* 9.67 (d, *J* = 1.0 Hz, 1H, H-3″), 9.43 (dd, *J* = 5.0, 1.0 Hz, 1H, H-6″), 7.99 (dd, *J* = 5.0, 2.0 Hz, 1H, H-5″), 4.15 (m, 2H, CH_2_-1′), 1.71 (m, 2H, CH_2_-2′), 1.80 (m, 2H, CH_2_-3′), 4.45 (t, *J* = 6.5 Hz, 2H, CH_2_-4′), 4.59 (s, 1H, H-29*E*), 4.72 (s, 1H, H-29*Z*), 2.99 (td, 1H, *J* = 11.5, 5.0 Hz, H-19), 2.38 (m, 1H, H-2a), 2.48 (m, 1H, H-2b), 2.25 (m, 1H, H-16b), 2.21 (m, 1H, H-13), 1.67, 1.05, 1.00, 0.96, 0.92, 0.89 (s, each 3H, 6 × −CH_3_). ^13^C NMR (125 MHz, CDCl_3_) *δ* 218.0, 176.1, 163.8, 152.2, 150.5, 149.7, 128.0, 125.5, 109.9, 66.1, 63.2, 56.7, 55.2, 50.1, 49.5, 47.5, 47.2, 42.7, 40.8, 39.8, 38.6, 37.2, 37.1, 34.3, 33.8, 32.2, 30.8, 29.8, 26.8, 25.7, 25.6 × 2, 21.6, 21.2, 19.8, 19.5, 16.1, 16.0, 14.8. HRMS (ESI) *m/z*: [M + H]^+^ 633.4271, calculated for C_39_H_57_N_2_O_5_ 633.4267.

### 2.2 Biology evaluation

#### 2.2.1 Cell culture

The human hepatocellular carcinoma cell line (HepG2), human hepatocellular carcinoma cell line (Bel-7402), human cervical cancer cell line (Hela), human breast adenocarcinoma cell line (MCF-7) and Madin-Darby canine kidney cell line (MDCK) were obtained from the Chinese Academy of Medical Sciences and Peking Union Medical College. Cultures were maintained as monolayer in RPMI-1640/DMEM supplemented with 10% (v/v) fetal bovine serum (FBS) and 1% (v/v) penicillin/streptomycin (Thermo Technologies, New York, NY, United States) under a humidified atmosphere containing 5% CO_2_ at 37°C. The BoA derivatives and positive control drugs (BoA and DDP) under study were dissolved in DMSO (Sigma, St. Louis, MO, United States) and added at required concentrations to the cell culture. Cells incubated without the preparations served as the control.

### 2.2.2 Antitumor activity

The antitumor activity was assessed by MTT assay, as previously reported (Fang et al., 2018). Each tested compound and positive control drugs were dissolved in DMSO and diluted with the medium to the test concentrations, and the final concentration of DMSO in the culture medium was controlled at less than 0.5% (v/v). Briefly, cells were cultured at 37°C and dispersed in replicates in 96-well plates with HepG2, Bel-7402, HeLa, MCF-7 and MDCK for 24 h. Fresh medium along with compounds (**BoAA-BoAE, BoA2A-BoA2G, BoA3A-BoA3G, BoA4A-BoA4G**) at different concentrations was then added to individual wells and incubated for 72 h, with BoA and DDP as the positive control. Experiments on FXR expression were separated into three groups: Control (PBS), **BoA2C** (8 μM), and **BoA2C** (8 μM) + DY268 (10 μM) (Giordano et al., 2016), with three repeating wells in each. In separate wells, **BoA2C** and DY268 were introduced and cultured for 72 h. After 72 h, the cell was incubated with MTT solution (0.5 mg/ml) for an additional 4 h at 37°C. The produced formazan crystals were solubilized with DMSO, and the optical density of solution was measured at 570 nm using a plate reader (BIORAD 550 spectrophotometer, Bio-rad Life Science Development Ltd., Beijing, China). Wells without drugs were used to be blanks. The IC_50_ values were defined as the concentration of compounds that produced a 50% proliferation inhibition of surviving cells and calculated using the Graph Pad Prism 5. The inhibitory rate was calculated in the following Equation 1, where OD is the optical density:

$$\%\;\mathrm{inhibition}=\left[1-\left(\mathrm{OD}{\;}_{\mathrm{Sample}\;\mathrm{group}}-\mathrm{OD}{\;}_{\mathrm{Blank}\;\mathrm{group}}\right)/\left(\mathrm{OD}{\;}_{\mathrm{Control}\;\mathrm{group}}-\mathrm{OD}{\;}_{\mathrm{Blank}\;\mathrm{group}}\right)\right]\times 100\%$$ (1)

### 2.2.3 DAPI staining

Morphological observation of nuclear changes was performed by DAPI staining in this assay. MCF-7 cells in the logarithmic growth phase were seeded in 12-well plates at a density of 5 × 10^3^ cells/well and were allowed to grow for 24 h. Then the cells were treated with different concentration ranges of BoA**2C** (0, 2, 4 8 μM) for 72 h. After the treatment period, cells were washed with PBS and fixed with 4% paraformaldehyde for 10 min. Then the liquid was discarded, and the cells were stained with DAPI (1 mg/ml, Molecular Probes/Invitrogen Life Technologies, Carlsbad, CA, United States) for 1 min in dark. After staining, cells were visualized under a fluorescence microscope (Olympus IX71, Tokyo, Japan).

### 2.2.4 Wound healing assay

MCF-7 cells were seeded in six-well plates at a density of 2 105 cells/ml and incubated at 37°C to achieve 100 percent confluence. After starving the cells for 24 h, a wound (cell-free) was drawn across the center of the well using the tip of a 1 ml plastic pipette. Wells were cleaned twice with PBS before being treated for 48 h with **BoA2C** (8 μM). At 0 h, 24 h, and 48 h, cells were viewed and photographed in randomly selected fields using an inverted light microscope (Nikon).

### 2.2.5 Cellular metabolomics study

To eliminate excess medium, cells were washed twice with 1 ml of PBS solution. The wells were then filled with 2 ml of ACN: MeOH: H_2_O 50:30:20 (v/v) and placed in the freezer for 20 min. The extracts obtained in an Eppendorf tube after the cells were scraped and vortexed. The mixture was then centrifuged for 10 min at 10,000 rpm and 4°C, and the supernatant was collected and dried with an evaporator. 300 μl MeOH was used for reconstitution. 15 μL of the reconstituted extract was mixed with 15 μl H_2_O and 70 L ACN for the metabolomics analysis of the intracellular material. After centrifugation (10 min, 4°C, 11,800 g), the supernatant was transferred to an LC-MS vial for analysis.

An Acquity UPLC HSS T3 column (1.8 μm 100 × 2.1 mm) using an UltiMate 3000 high performance LC system coupled to Q Exactive MS was used for metabolic profiling (Shu et al., 2020). At a flow rate of 0.3 ml/min, mobile phase A (water containing 0.1% acid) and mobile phase B (methanol) were used. A was reduced from 80 % to 70% from 0 to 2 min, A was reduced to 55% from 2 to 5 min, A was reduced to 40% from 5 to 6.5 min, A was reduced to 35% from 6.5 to 12 min, A was last reduced to 0 from 12 to 18 min. The gradient of the mobile phase was as follows: The column oven temperature was set to 40°C, and the autosampler temperature was set to 4°C. The injection volume was 1 μl. MS used electron spray ionization to operate in both positive and negative ionization modes (ESI). To assess the stability and repeatability of the LC-MS-based metabolomics method, quality control (QC) samples were used. Each QC sample was made by combining five samples of the same volume (100 μL) in the order of analysis. To ensure the system method’s stable operation, all combined QC samples for running all QC samples made by every five samples before analysis. After five samples were tested during the analysis run, the combined QC samples of the five samples were taken.

### 2.3 Statistical analysis

The data were expressed as mean ± SD. Statistical testing using GraphPad Prism 8.0.2. The observed data were compared and analyzed by one-way ANOVA. The levels of statistical significance were set at ∗*p* < 0.05, ∗∗*p* < 0.01, ∗∗∗*p* < 0.001.

## 3 Results and discussion

### 3.1 Chemistry

**SCHEME 1:**
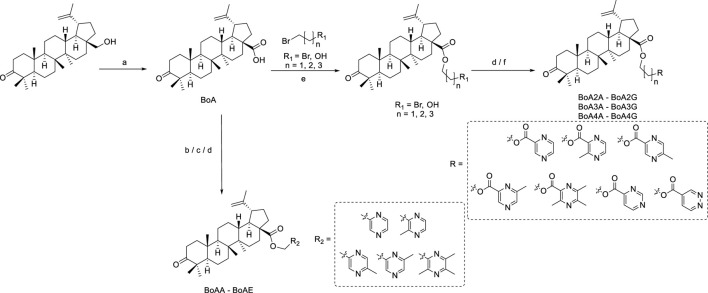
Synthesis of the BoA and its derivatives **BoAA-BoAE**. Reagents and Conditions: **(A)** K_2_Cr_2_O_7_, Al_2_O_3_, H_2_SO_4_, Acetone, H_2_O, rt., 3 h; **(B)** K_2_CO_3_, DMF, rt., 2 h; **(C)** (i) SOCl_2_, CH_2_Cl_2_, rt., 2 h; (ii) K_2_CO_3_, DMF, rt., 2 h; **(D)** K_2_CO_3_, DMF, 85°C, 4 h; **(E)** K_2_CO_3_, DMF, rt., 4 h; **(F)** CH_2_Cl_2_, EDCI, DMAP, rt., 4 h.

The designed derivatives were prepared following the procedures in Scheme 1. The compound BoA was synthesized from botulin according to the route depicted in Scheme 1, as in detail shown previously (Krasutsky, 2006). In Scheme 1, **BoAA** and **BoAB** were synthesized through a combination of BoA and chloromethyl pyrazine in N, N-dimethylformamide (DMF) containing K_2_CO_3_ at room temperature, and the yield was 49%–62%. And **BoAE** was obtained by the same synthetic route at 85°C instead of at room temperature with 82% yield. In addition, methyl-2-pyrazinylmethanol in dry dichloromethane (DCM) containing dichlorosulfoxide (SOCl_2_) was allowed to stir at room temperature, which was further reacted with BoA in dry DMF in the presence of K_2_CO_3_ to yield compounds **BoAC** and **BoAD**.

As seen in Scheme 1, the intermediates **BoAnBr/BoAnOH** (*n* = 2, 3, 4) were obtained through a combination of BoA and 1, 2-dibromoethane/1, 3-dibromopropane/1, 4-dibromobutane/2-bromoethanol/3-bromo-1-propanol/4-chloro-1-butanol containing K_2_CO_3_ at room temperature in DMF. It is worth mentioning that dihalogenated carbon chain should be 3–4 times as much as BoA when it was reacted with BoA in order to avoid the formation of dipolymer. The compounds **BoAnB-BoAnE** (*n* = 2, 3, 4) were produced with 60%–80% yield through a combination of the intermediates **BoAnBr** (n = 2, 3, 4) and methyl-2-pyrazinic acid by the same synthetic route of **BoAE**. Interestingly, the compounds **BoAnA, BoAnF-BoAnG** (*n* = 2, 3, 4) were synthesized through the combination of the intermediates **BoAnOH** (*n* = 2, 3, 4) and 2-pyrazinic acid/4-pyrimidinic acid/4-pyridazinic acid using 1-ethyl-3-(3-dimethylaminopropyl) carbodiimide hydrochloride (EDCI) and 4-dimethylaminopyridine (DMAP) in dry DCM. All reactions were carried out as detailed in the experimental section, and the structures of all target derivatives (Table 1) were confirmed by spectral (^1^H NMR, ^13^C NMR and HRMS) analysis (spectra data can be found in Supplementary Materials).

**TABLE 1:** Structures of the BoA derivatives.

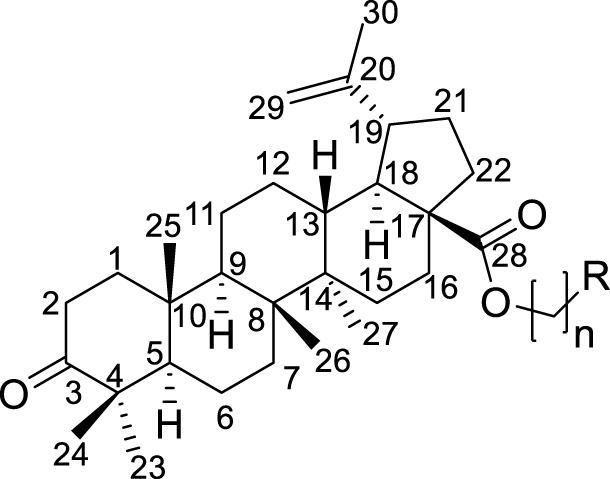
Compound	n	R	Compound	n	R
BoAA	1	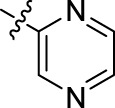	BoA3B	3	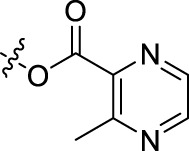
BoAB	1	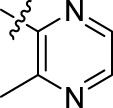	BoA3C	3	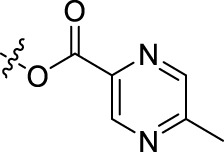
BoAC	1	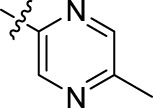	BoA3D	3	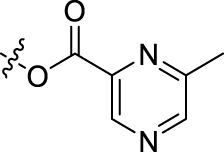
BoAD	1	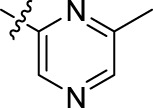	BoA3E	3	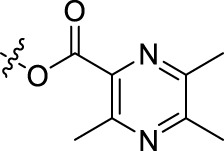
BoAE	1	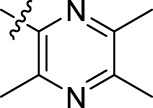	BoA3F	3	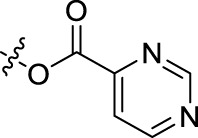
BoA2A	2	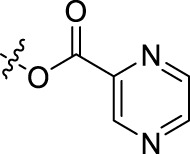	BoA3G	3	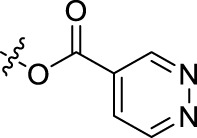
BoA2B	2	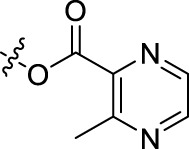	BoA4A	4	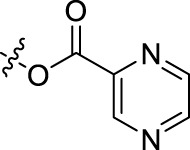
BoA2C	2	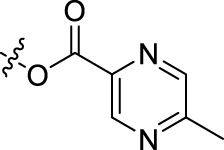	BoA4B	4	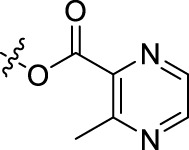
BoA2D	2	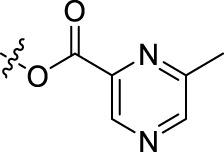	BoA4C	4	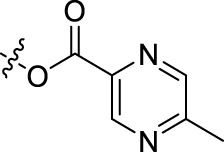
BoA2E	2	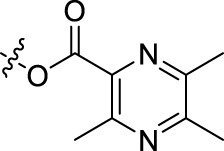	BoA4D	4	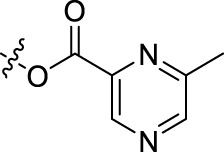
BoA2F	2	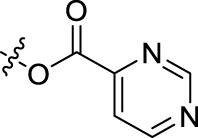	BoA4E	4	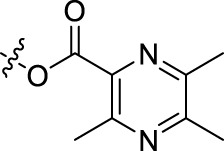
BoA2G	2	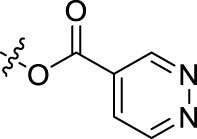	BoA4F	4	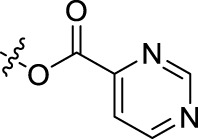
BoA3A	3	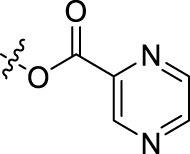	BoA4G	4	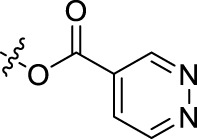

### 3.2 Biological screening

#### 3.2.1 Cytotoxicity assay

**FIGURE 3:**
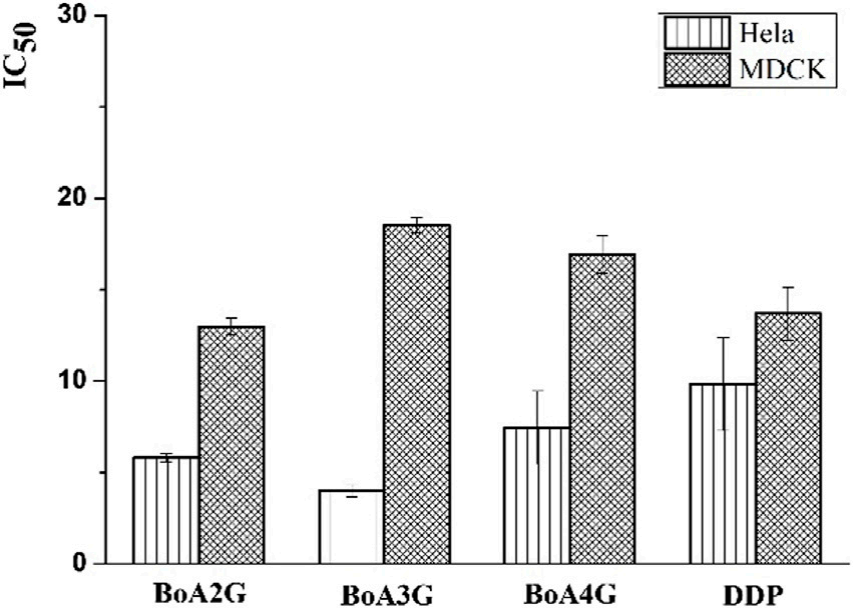
Comparison of cytotoxicity between BoAnG (*n* = 2, 3, 4) and DDP.

**FIGURE 4:**
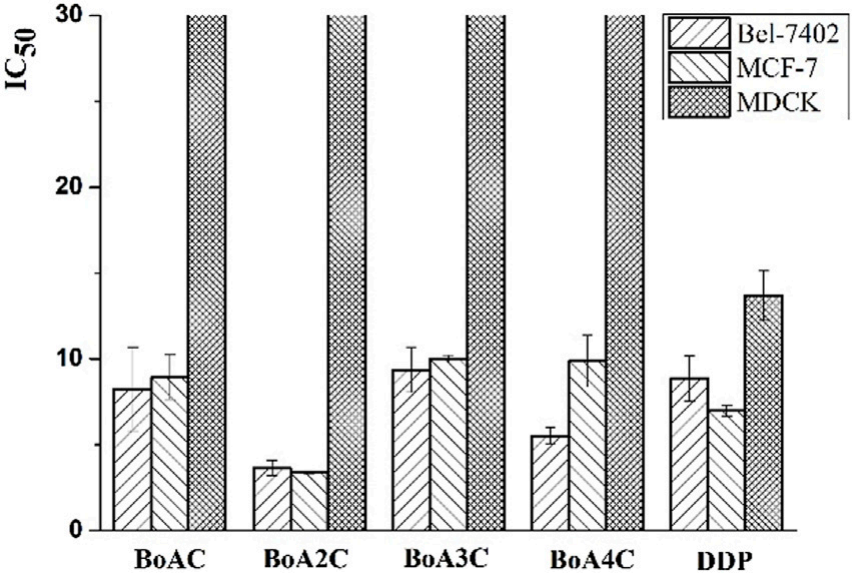
Comparison of cytotoxicity between BoAnC (*n* = 1, 2, 3, 4) and DDP.

The *in vitro* cytotoxicity of BoA derivatives was evaluated on four tumor cell lines (HepG2, Bel-7402, Hela, MCF-7) and MDCK cell line using the standard MTT assay. The IC_50_ values of these compounds were summarized in Table 2. The results revealed that the majority of the compounds considerably increased cytotoxicity in all tumor cell lines examined compared to the BoA, while they showed lower cytotoxicity than DDP on MDCK cell line (13.69 ± 1.45 μM). Among the compounds, **BoA3G** possessed pronounced cytotoxicity against the cell lines Hela at the IC_50_ levels of 4.00 ± 0.32 μM (Figure 3). It's worth noting that the most active compound was **BoA2C**, which revealed more severe cytotoxicity against Bel-7402 and MCF-7 cells at the IC_50_ levels of 3.65 ± 0.43 μM and 3.39 ± 0.07 μM, comparing with the cisplatin (DDP) against those cells at the IC_50_ levels of 8.86 ± 1.32 μM and 6.98 ± 0.32 μM, respectively. Meanwhile, **BoA2C** exhibited lower cytotoxic against MDCK cell (IC_50_ > 30 μM) than DDP (Figure 4).

**TABLE 2:** Inhibition effects of **BoA** derivatives on the growth of tumor cells and MDCK cells *in vitro*.

	Half maximal inhibitory concentration (IC_50_) values (*µ*M)				
Compound	HepG2	Bel-7402	Hela	MCF-7	MDCK
BoAA	13.47 ± 1.80	8.16 ± 2.08	18.27 ± 1.63	15.66 ± 0.55	>30
BoAB	11.08 ± 0.27	7.73 ± 1.92	11.80 ± 0.58	10.02 ± 0.26	>30
BoAC	8.95 ± 4.76	8.24 ± 2.43	11.91 ± 1.05	8.94 ± 1.30	>30
BoAD	5.17 ± 1.33	7.51 ± 1.40	10.69 ± 0.32	8.24 ± 0.87	>30
BoAE	-^a^	-	-	-	-
BoA2A	13.73 ± 0.25	8.76 ± 0.84	24.97 ± 0.97	9.86 ± 0.36	>30
BoA2B	13.44 ± 1.82	10.06 ± 1.40	15.48 ± 0.81	>30	>30
**BoA2C**	7.46 ± 1.43	3.65 ± 0.43	15.59 ± 2.63	3.39 ± 0.07	>30
BoA2D	7.32 ± 2.88	8.22 ± 0.43	14.93 ± 1.84	8.32 ± 1.28	>30
BoA2E	-	-	-	-	-
BoA2F	>30	>30	15.20 ± 1.07	29.65 ± 0.37	12.50 ± 1.22
BoA2G	>30	24.81 ± 2.43	5.81 ± 0.24	>30	12.97 ± 0.46
BoA3A	4.82 ± 0.30	6.09 ± 0.12	20.41 ± 0.56	6.85 ± 1.82	>30
BoA3B	13.16 ± 1.20	14.24 ± 2.10	15.71 ± 2.15	27.88 ± 1.06	>30
BoA3C	>30	9.37 ± 1.31	29.98 ± 2.65	10.02 ± 0.19	>30
BoA3D	8.65 ± 0.60	6.42 ± 0.66	9.50 ± 0.28	5.60 ± 0.25	17.01 ± 3.01
BoA3E	20.18 ± 0.53	11.21 ± 1.55	10.13 ± 0.15	29.48 ± 0.89	>30
BoA3F	>30	22.31 ± 5.85	21.09 ± 6.66	>30	17.93 ± 0.82
BoA3G	>30	25.25 ± 2.44	4.00 ± 0.32	13.44 ± 0.51	18.53 ± 0.42
BoA4A	12.89 ± 4.00	9.39 ± 0.73	13.82 ± 0.12	9.13 ± 0.40	25.32 ± 4.14
BoA4B	21.83 ± 2.46	3.92 ± 1.10	11.47 ± 0.93	>30	>30
BoA4C	22.18 ± 1.76	5.53 ± 0.46	17.87 ± 0.45	9.88 ± 1.49	>30
BoA4D	>30	16.29 ± 0.90	15.74 ± 2.15	>30	>30
BoA4E	>30	>30	>30	>30	>30
BoA4F	30.23 ± 1.51	>30	25.86 ± 0.57	24.47 ± 5.17	27.22 ± 0.25
BoA4G	29.27 ± 0.22	19.43 ± 1.20	7.46 ± 1.99	12.77 ± 0.15	16.93 ± 1.03
BoA	>30	>30	27.04 ± 1.06	>30	>30
DDP^b^	5.23 ± 1.03	8.86 ± 1.32	9.84 ± 2.54	6.98 ± 0.32	13.69 ± 1.45

a “-” was meant that the IC_50_ values of the compounds can’t be determined accurately because of their poor solubility in DMSO.

b DDP (cisplatin) was used as positive control.

Structure-activity relationships (SAR) investigations revealed that BoA derivatives including diazines have varying selectivity for four distinct human cancer cells, with pyrazine derivatives showing the best selectivity. The amount of methyl groups in pyrazine derivatives is critical, since the anticancer activity drops dramatically as the number of methyl groups increases. Despite their improved anticancer activity, pyrimidine and pyridazine compounds exhibited no selectivity. Besides, it was observed that the compounds with pyridazine had stronger activity on Hela cells, and those with 2, 5-dimethylpyrazine seemed to be more active on Bel-7402 and MCF-7 cells. However, there was no obvious rule that the relationship among the differing length of carbon chain between BoA and diazines and cytotoxic activity against a variety of cancer cell lines. In this study, the most promising compound **BoA2C** was selected for further analysis to reveal its preliminary mechanism of growth inhibition on MCF-7 cells and MDCK cells.

### 3.2.2 Analyses of cell staining

**FIGURE 5:**
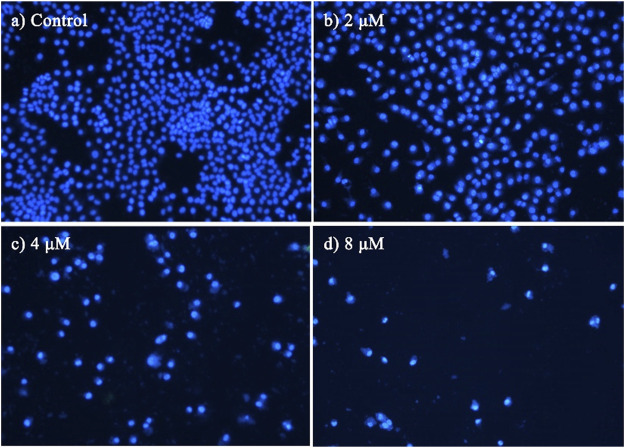
Morphological detection of apoptosis using DAPI staining (200×) on MCF-7 cells treated with **BoA2C**: **(A)** control group; **(B)** 2 μM; **(C)** 4 μM; **(D)** 8 μM.

**FIGURE 6:**
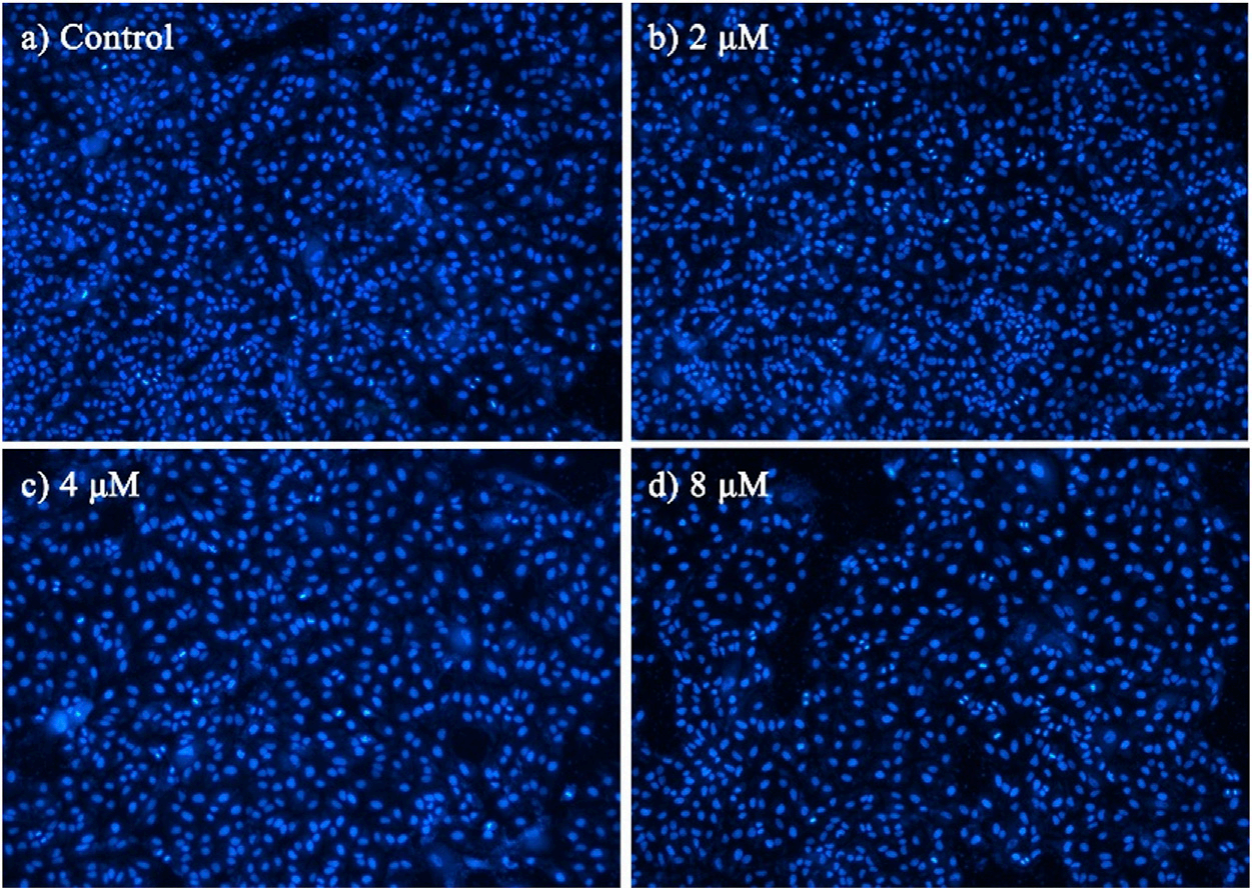
Morphological detection of apoptosis using DAPI staining (100×) on MDCK cells treated with **BoA2C**: **(A)** control group; **(B)** 2 μM; **(C)** 4 μM; **(D)** 8 μM.

To characterize the effects of apoptosis inducted by **BoA2C** on MCF-7 cell and MDCK cell, the nuclear morphological changes in **BoA2C**-treated MCF-7 and MDCK were observed by DAPI staining. As shown in Figure 5, the morphology of MCF-7 cells in the negative control was normal, after treated with **BoA2C** (2, 4, 8 μM) for 72 h, MCF-7 cells showed nuclear morphological changes typical of apoptosis in a dose-dependent manner. Even at low concentration of **BoA2C** (2 μM), In terms of morphological characteristics, the cells had displayed typical death, such as distinctive condensation, margination of chromatin and nuclear fragmentation. With the increase of the concentration of **BoA2C**, the characteristic of proliferation inhibition was more and more obvious. When the concentration of **BoA2C** was raised up to 8 μM, the number of cells decreased drastically, the shape of the cells became irregular, and nuclear fragmentation occurred. In contrast, as seen in Figure 6, MDCK cells did not show a significant nuclear damage with increasing concentration of **BoA2C**. And these results indicated that **BoA2C** significantly induced apoptosis in MCF-7 cells but less damaged to MDCK cells.

#### 3.2.3 Metabolomics study in MCF-7 cells treated with **BoA2C**

**FIGURE 7:**
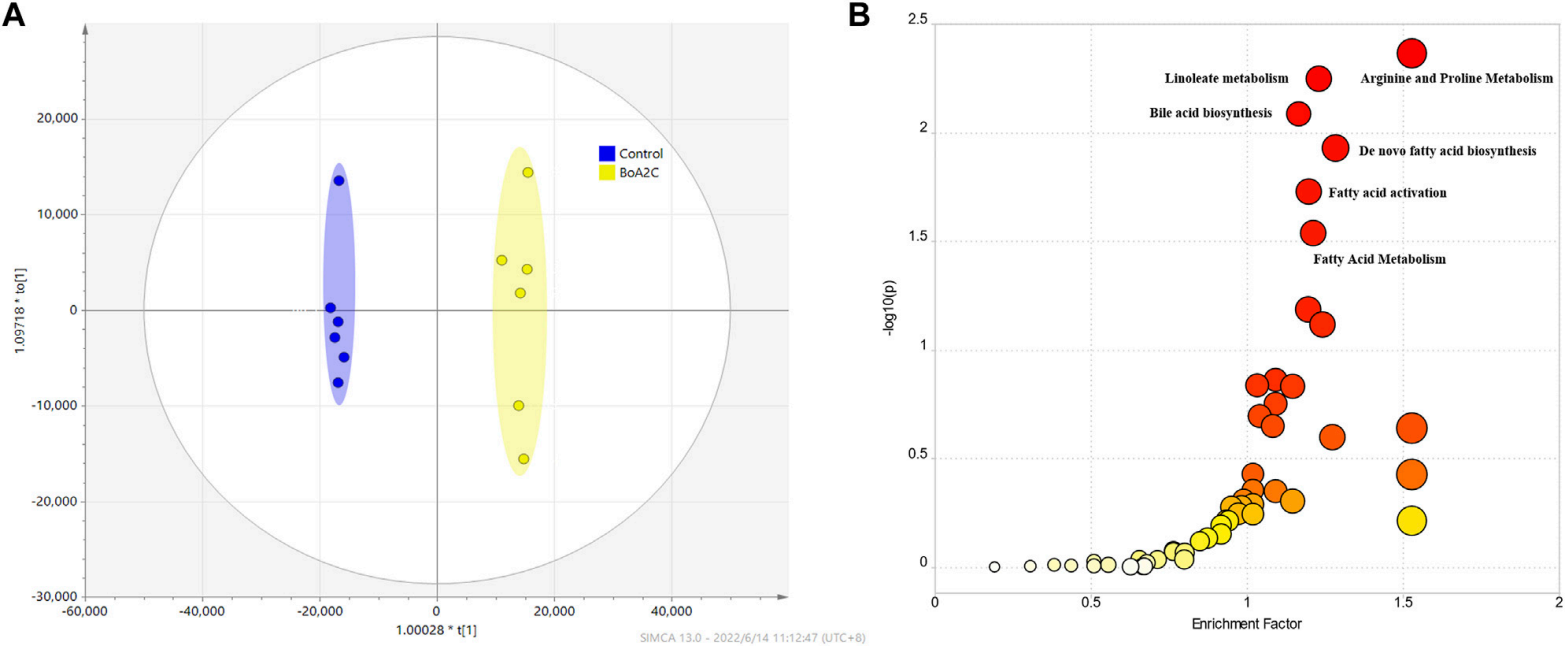
Metabolomics study for **BoA2C** in MCF-7 cells. **(A)** OPLS-DA score plots of **BoA2C** and control group. **(B)** KEGG pathway enrichment analysis of differential metabolites between **BoA2C** and control group.

Mass spectrometry data of metabolites in MCF-7 cells was used for metabolomics investigation after co-cultivation with **BoA2C** (2 μM) for 72 h. The effectiveness of the OPLS-DA model to distinguish between the **BoA2C** and Control groups was assessed using the success criterion of R^2^ ≥ 0.7 and Q^2^ ≥ 0.4 (Martin-Blazquez et al., 2019). The current model has an R2 of 0.994 and a Q2 of 0.982, indicating that it is capable of distinguishing between **BoA2C** and Control samples in Figure 7A. VIP-plots were investigated using the OPLS-DA models to examine important factors that led to the differentiation of the metabolome between the **BoA2C** and Control groups. Variables having a VIP value greater than 1 were deemed significant contenders. Matching retention time and MS fragmentation patterns from the MetaboAnalyst (version 5.0) database verified the presence of distinct metabolites (Pang et al., 2021). These metabolites were further categorized based on their characteristics, including amino acids, amines, aliphatic acids and bile acids. All differential metabolites were loaded into MetaboAnalyst (version 5.0) for metabolic analysis to further explain the fundamental mechanisms of **BoA2C**’s cytotoxic impact on breast carcinoma cells. In accordance with the described guidelines, the *Homo sapiens* pathway library and Fisher’s exact test were used for route enrichment analysis, while relative-betweeness centrality was used for pathway topology analysis (Pang et al., 2021). The impacted metabolic pathways were defined as those with a pathway effect value more than 0.10 and a *p* value less than 0.05. According to the *p* and pathway impact values, 15 metabolic pathways were observed in **BoA2C** and Control groups (Figure 7B). Six of them (Figure 7B, Table 3) were primarily involved: 1) arginine and proline metabolism 2) linoleate metabolism 3) bile acid biosynthesis 4) *de novo* fatty acid biosynthesis 5) fatty acid activation, and (VI) fatty Acid Metabolism.

**TABLE 3:** Potential biomarker from primarily involved six metabolic pathways.

no	name	molecular formula	adduct	observed mass	exact mass	Mass error (ppm)	rt (min)	VIP value	fold change	*p* value	metabolic pathway
M1	Cholesterol	C_27_H_46_O	[M-H_2_O + H] ^+^	369.3508	386.3548	-1.9	16.58	10.04	7.69	2.20 × 10^−4^	Bile acid biosynthesis
M2	Cholic acid	C_24_H_40_O_5_	[M-H_2_O + H] ^+^	391.2834	408.2875	-2.0	16.32	9.22	2.55	4.05 × 10^−3^	Bile acid biosynthesis
M3	3 alpha,7 alpha,26-Trihydroxy-5beta-cholestane	C_27_H_48_O_3_	[M + Na] ^+^	443.3513	420.3603	4.1	13.16	3.89	362.98	1.58 × 10^−13^	Bile acid biosynthesis
M4	4-Acetamidobutanoic acid	C_6_H_11_NO_3_	[M-H2O + H] ^+^	128.0704	145.0738	-0.8	1.52	3.64	2.37	3.61 × 10^−5^	Arginine and Proline Metabolism
M5	L-Proline	C_5_H_9_NO_2_	[M + H] ^+^	116.0704	115.0633	-1.7	1.29	2.99	2.40	8.91 × 10^−9^	Arginine and Proline Metabolism
M6	L-4-Hydroxyglutamate semialdehyde	C_5_H_9_NO_4_	[M + H] ^+^	148.0601	147.0531	-2.0	1.29	2.95	1.99	3.89 × 10^−11^	Arginine and Proline Metabolism
M7	L-Glutamine	C_5_H_10_N_2_O_3_	[M + H] ^+^	147.0762	146.0691	-1.4	1.31	2.92	2.33	2.66 × 10^−10^	Arginine and Proline Metabolism
M8	LysoPC(P-18:0/0:0)	C_26_H_54_NO_6_P	[M + H] ^+^	508.3757	507.3688	-0.8	11.84	2.87	3.29	3.82 × 10^−9^	Linoleate metabolism
M9	13-Hpode	C_18_H_32_O_4_	[M + Na] ^+^	335.2210	312.2301	5.1	6.35	2.74	23.04	1.68 × 10^−11^	Linoleate metabolism
M10	5′-Methylthioadenosine	C_11_H_15_N_5_O_3_S	[M + H] ^+^	298.0962	297.0895	-2.0	1.54	2.73	28.55	1.92 × 10^−12^	Arginine and Proline Metabolism
M11	Palmitic acid	C_16_H_32_O_2_	[M + Na] ^+^	279.2312	256.2402	6.4	11.18	2.53	23.17	1.08 × 10^−11^	*De novo* fatty acid biosynthesis
M12	L-Methionine	C_5_H_11_NO_2_S	[M + H] ^+^	150.0581	149.0510	-1.3	1.32	2.42	3.63	1.41 × 10^−8^	Arginine and Proline Metabolism
M13	3a,7a-Dihydroxy-5b-cholestan-26-al	C_27_H_46_O_3_	[M + Na] ^+^	441.3364	418.3446	5.9	14.93	2.28	241.46	4.33 × 10^−10^	Bile acid biosynthesis
M14	L-Arginine	C_6_H_14_N_4_O_2_	[M + H] ^+^	175.1187	174.1116	-1.1	0.97	2.22	1.90	1.39 × 10^−8^	Arginine and Proline Metabolism
M15	3-Oxotetradecanoyl-CoA	C_35_H_60_N_7_O_18_P_3_S	[M-H_2_O + H] ^+^	974.2870	991.2928	-2.6	1.30	2.08	1.77	4.88 × 10^−8^	Fatty Acid Metabolism
M16	7a,12a-Dihydroxy-cholestene-3-one	C_27_H_44_O_3_	[M + H] ^+^	417.3356	416.329	-1.7	11.42	1.92	17.04	8.24 × 10^−11^	Bile acid biosynthesis
M17	7alpha-Hydroxycholesterol	C_27_H_46_O_2_	[M + Na] ^+^	425.3411	402.3497	5.2	13.28	1.87	92.35	1.78 × 10^−8^	Bile acid biosynthesis
M18	L-Glutamic acid	C_5_H_9_NO_4_	[M-H_2_O + H] ^+^	130.0497	147.0531	-0.8	1.36	1.86	1.94	3.80 × 10^−7^	Arginine and Proline Metabolism
M19	Ascorbic Acid	C_6_H_8_O_6_	[M-H_2_O + H] ^+^	159.0274	176.0321	-8.8	1.19	1.72	2.88	8.29 × 10^−10^	Linoleate metabolism
M20	Linoleic acid	C_18_H_32_O_2_	[M + Na] ^+^	303.2311	280.2402	5.6	12.83	1.57	159.58	7.93 × 10^−12^	Linoleate metabolism
M21	3,7-Dihydroxycoprostanic Acid	C_27_H_46_O_4_	[M + Na] ^+^	457.3314	434.3396	5.7	11.82	1.54	1027.39	6.53 × 10^−12^	Bile acid biosynthesis
M22	2-trans-dodecenoyl-CoA	C_33_H_56_N_7_O_17_P_3_S	[M + H] ^+^	948.2762	947.2666	2.4	1.37	1.51	1.93	4.45 × 10^−5^	Fatty Acid Metabolism
M23	13-HODE	C_18_H_32_O_3_	[M + H] ^+^	315.2522	296.2351	-2.5	8.55	1.43	108.54	7.47 × 10^−10^	Linoleate metabolism
M24	27-Deoxy-5b-cyprinol	C_27_H_48_O_4_	[M + Na] ^+^	459.3459	436.3552	3.3	3.28	1.43	2.03	2.47 × 10^−3^	Bile acid biosynthesis
M25	7a-Hydroxy-5b-cholestan-3-one	C_27_H_46_O_2_	[M + Na] ^+^	425.3403	402.3497	3.3	16.17	1.30	9.40	1.06 × 10^−7^	Bile acid biosynthesis
M26	alpha-Linolenic acid	C_18_H_30_O_2_	[M + H_2_O + H] ^+^	297.2417	278.2245	-2.4	13.59	1.27	2.19	2.42 × 10^−9^	*De novo* fatty acid biosynthesis
M27	Lithocholic acid	C_24_H_40_O_3_	[M-H_2_O + H] ^+^	359.2935	376.2977	-2.5	14.88	1.26	108.49	4.27 × 10^−10^	Bile acid biosynthesis
M28	7a-Hydroxy-cholestene-3-one	C_27_H_44_O_2_	[M-H_2_O + H] ^+^	383.3299	400.3341	-2.3	10.75	1.15	4.47	7.13 × 10^−4^	Bile acid biosynthesis

**FIGURE.8:**
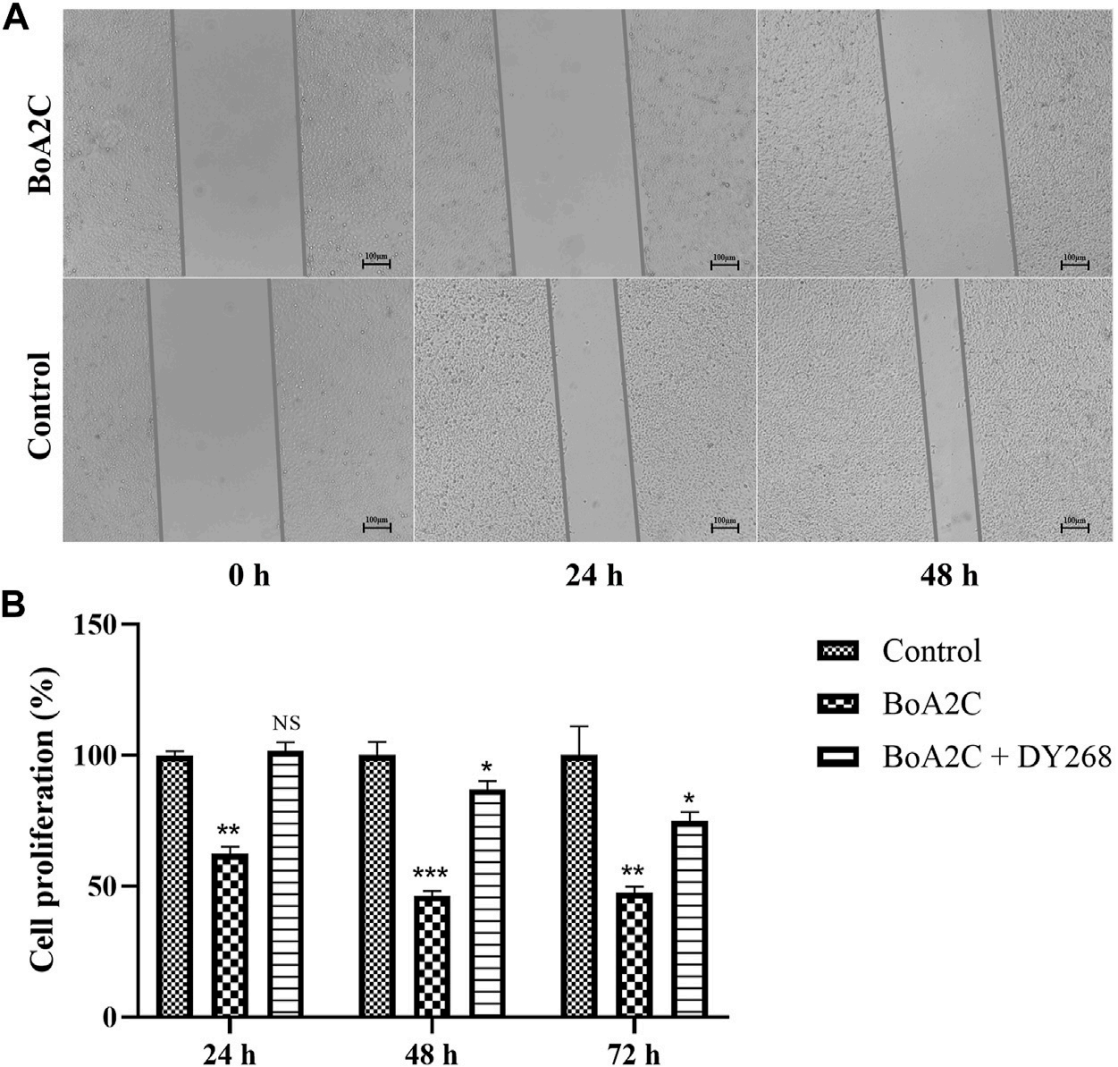
The effect of **BoA2C** on the invasive ability of MCF-7 cells and the role of FXR in the anticancer process. **(A)** Wound healing assays were performed in 0, 24, 48 h; **(B)** Changes of antiproliferative activity under DY268. *p < 0.05, **p < 0.01, and ***p < 0.001 vs. control cells.

Arginine is a type of multi-functional amino acid involved in the synthesis of many metabolites, such as nitric oxide, urea, ornithine, and citrulline, and is involved in protein modification and immunoregulation (Morris, 2007; Rath et al., 2014; Szefel et al., 2019). ASS1, one of the key enzymes involved in arginine metabolism, is highly expressed in normal tissues but heterogeneously expressed in tumors (Dillon et al., 2004). These researchers found that ASS1 was completely deficient in HCC and prostate cancer and partially deficient in breast cancer (Dillon et al., 2004; Morettin et al., 2015). Several recent investigations have shown that arginine deprivation caused tumor cell death *via* degrading extracellular arginine (Alexandrou et al., 2018; Nasreddine et al., 2020; Chu et al., 2021). This suggested that arginine shortage might occur in MCF-7 cells treated with **BoA2C** based on the noticeably changed in arginine metabolism. Bile acid composition is controlled via the modulation of bile acid-metabolizing enzymes and bile acid transporters, the majority of which are expressed by farnesoid X receptor (FXR) target genes (Sun et al., 2021). FXR regulates the expression of target genes involved in both bile acid production and fatty acid biosynthesis, which is required for the maintenance of whole-body bile acid homeostasis (Matsubara et al., 2013; Chiang and Ferrell, 2020). Furthermore, FXR stimulation weakened cell proliferation and migration under steroid-free media conditions in MCF-7 and T47D breast cancer cells, and other investigators observed that FXR agonists caused apoptosis and inhibited local estrogen synthesis (Alasmael et al., 2016; Giordano et al., 2016; Barone et al., 2018). As shown in Figure 8A, the scratch wounds and migratory cells in the wound were imaged at 0, 24, and 48 h after scratched in this study. The results demonstrated that the treatment of **BoA2C** obviously reduced the migration of MCF-7 cells in the scratch compared to control group, thus potentially inhibited tumor invasion with enhanced FXR expression. Then, a potent FXR antagonist (DY268) was employed (Yu et al., 2014; Hankir et al., 2020) in a cell proliferation investigation to better evaluate the regulatory link between FXR expression and **BoA2C** treatment. The results (Figure 8B) unequivocally demonstrated that DY268 reversed **BoA2C** inhibitory impact on the growth of MCF-7 breast cancer cells in both 24, 48 and 72 h. Thus, **BOA2C** could disrupt bile acid and fatty acid metabolism and limit tumor development and invasion probably by activating FXR expression.

## 4 Conclusion

In this study, 26 new betulonic acid-diazines were designed and synthesized from BoA and all of them were characterized by ^1^H NMR, ^13^C NMR, HRMS. Furthermore, all these compounds were tested for cytotoxic activity against HepG2, Bel-7402, Hela, MCF-7 and MDCK cell lines by standard MTT assay. After exploring the structure-activity relationship, we found that the introduction of different groups at C-28 can selectively inhibit different kinds of cancer cells. The introduction of a pyridazine structure at the C-28 of BoA was possessed a stronger cytotoxic activity against Hela cell. It is interesting that the introduction of a 2, 5-dimethylpyrazine structure might be more effective cytotoxicity against Bel-7402 and MCF-7 cells and lower cytotoxicity on MDCK than DDP. From the obtained results, compounds **BoA2C** exerted potent and selective *in vitro* antiproliferative activity. As shown in metabolomics results, it was found that **BoA2C** significantly induced cell injury in MCF-7 cells mostly through the interference of arginine metabolism and fatty acid metabolism. It’s presently undertaking intense adjustments at C-28 and investigations in the mechanism of **BoA2C**’s antiproliferative impact in our further studies, and the results will be revealed in due course.

## Data Availability

The original contributions presented in the study are included in the article/Supplementary Material, further inquiries can be directed to the corresponding authors.

## References

[B1] Alasmael N. Mohan R. Meira L. B. Swales K. E. Plant N. J. (2016). Activation of the Farnesoid X-receptor in breast cancer cell lines results in cytotoxicity but not increased migration potential. Cancer Lett. 370 (2), 250–259. 10.1016/j.canlet.2015.10.031 PubMed Abstract | 10.1016/j.canlet.2015.10.031 | Google Scholar 26545738

[B2] Alexandrou C. Al-Aqbi S. S. Higgins J. A. Boyle W. Karmokar A. Andreadi C. (2018). Sensitivity of colorectal cancer to arginine deprivation therapy is shaped by differential expression of urea cycle enzymes. Sci. Rep. 8 (1), 12096. 10.1038/s41598-018-30591-7 PubMed Abstract | 10.1038/s41598-018-30591-7 | Google Scholar 30108309 PMC6092409

[B3] Ayers S. Benkovics T. Marshall J. Tan Y. Strotman N. A. Kiau S. (2016). Autoxidation products of betulonaldehyde. J. Nat. Prod. (Gorakhpur). 79 (10), 2758–2761. 10.1021/acs.jnatprod.6b00735 PubMed Abstract | 10.1021/acs.jnatprod.6b00735 | Google Scholar 27684353

[B4] Baratto L. C. Porsani M. V. Pimentel I. C. Pereira Netto A. B. Paschke R. Oliveira B. H. (2013). Preparation of betulinic acid derivatives by chemical and biotransformation methods and determination of cytotoxicity against selected cancer cell lines. Eur. J. Med. Chem. 68, 121–131. 10.1016/j.ejmech.2013.07.012 PubMed Abstract | 10.1016/j.ejmech.2013.07.012 | Google Scholar 23973824

[B5] Barone I. Vircillo V. Giordano C. Gelsomino L. Gyorffy B. Tarallo R. (2018). Activation of Farnesoid X Receptor impairs the tumor-promoting function of breast cancer-associated fibroblasts. Cancer Lett. 437, 89–99. 10.1016/j.canlet.2018.08.026 PubMed Abstract | 10.1016/j.canlet.2018.08.026 | Google Scholar 30176263

[B6] Bi S. Chu F. Wang M. Li B. Mao P. Zhang H. (2016). Ligustrazine-oleanolic acid Glycine derivative, G-TOA, selectively inhibited the proliferation and induced apoptosis of activated HSC-T6 cells. Molecules 21 (11), 1599. 10.3390/molecules21111599 PubMed Abstract | 10.3390/molecules21111599 | Google Scholar PMC6273822 27886086

[B7] Chiang J. Y. L. Ferrell J. M. (2020). Bile acid receptors FXR and TGR5 signaling in fatty liver diseases and therapy. Am. J. Physiology-Gastrointestinal Liver Physiology 318 (3), G554–G573. 10.1152/ajpgi.00223.2019 PubMed Abstract | 10.1152/ajpgi.00223.2019 | Google Scholar PMC7099488 31984784

[B8] Chu C. Y. Lee Y. C. Hsieh C. H. Yeh C. T. Chao T. Y. Chen P. H. (2021). Genome-wide CRISPR/Cas9 knockout screening uncovers a novel inflammatory pathway critical for resistance to arginine-deprivation therapy. Theranostics 11 (8), 3624–3641. 10.7150/thno.51795 PubMed Abstract | 10.7150/thno.51795 | Google Scholar 33664852 PMC7914361

[B9] Damle A. A. Pawar Y. P. Narkar A. A. (2013). Anticancer activity of betulinic acid on MCF-7 tumors in nude mice. Indian J. Exp. Biol. 51 (7), 485–491. PubMed Abstract | Google Scholar 23898546

[B10] Dangroo N. A. Singh J. Rath S. K. Gupta N. Qayum A. Singh S. (2017). A convergent synthesis of novel alkyne-azide cycloaddition congeners of betulinic acid as potent cytotoxic agent. Steroids 123, 1–12. 10.1016/j.steroids.2017.04.002 PubMed Abstract | 10.1016/j.steroids.2017.04.002 | Google Scholar 28435038

[B11] Dillon B. J. Prieto V. G. Curley S. A. Ensor C. M. Holtsberg F. W. Bomalaski J. S. (2004). Incidence and distribution of argininosuccinate synthetase deficiency in human cancers: A method for identifying cancers sensitive to arginine deprivation. Cancer 100 (4), 826–833. 10.1002/cncr.20057 PubMed Abstract | 10.1002/cncr.20057 | Google Scholar 14770441

[B12] Dinh Ngoc T. Moons N. Kim Y. De Borggraeve W. Mashentseva A. Andrei G. (2014). Synthesis of triterpenoid triazine derivatives from allobetulone and betulonic acid with biological activities. Bioorg. Med. Chem. 22 (13), 3292–3300. 10.1016/j.bmc.2014.04.061 PubMed Abstract | 10.1016/j.bmc.2014.04.061 | Google Scholar 24844757

[B13] Eignerova B. Tichy M. Krasulova J. Kvasnica M. Rarova L. Christova R. (2017). Synthesis and antiproliferative properties of new hydrophilic esters of triterpenic acids. Eur. J. Med. Chem. 140, 403–420. 10.1016/j.ejmech.2017.09.041 PubMed Abstract | 10.1016/j.ejmech.2017.09.041 | Google Scholar 28987603

[B14] Fang K. Zhang X. H. Han Y. T. Wu G. R. Cai D. S. Xue N. N. (2018). Design, synthesis, and cytotoxic analysis of novel Hederagenin(-)Pyrazine derivatives based on partial least squares discriminant analysis. Int. J. Mol. Sci. 19 (10), 2994. 10.3390/ijms19102994 PubMed Abstract | 10.3390/ijms19102994 | Google Scholar PMC6213900 30274380

[B15] Giordano C. Barone I. Vircillo V. Panza S. Malivindi R. Gelsomino L. (2016). Activated FXR inhibits leptin signaling and counteracts tumor-promoting activities of cancer-associated fibroblasts in breast malignancy. Sci. Rep. 6, 21782. 10.1038/srep21782 PubMed Abstract | 10.1038/srep21782 | Google Scholar 26899873 PMC4761870

[B16] Grishko V. V. Tolmacheva I. A. Nebogatikov V. O. Galaiko N. V. Nazarov A. V. Dmitriev M. V. (2017). Preparation of novel ring-A fused azole derivatives of betulin and evaluation of their cytotoxicity. Eur. J. Med. Chem. 125, 629–639. 10.1016/j.ejmech.2016.09.065 PubMed Abstract | 10.1016/j.ejmech.2016.09.065 | Google Scholar 27721148

[B17] Gupta N. Rath S. K. Singh J. Qayum A. Singh S. Sangwan P. L. (2017). Synthesis of novel benzylidene analogues of betulinic acid as potent cytotoxic agents. Eur. J. Med. Chem. 135, 517–530. 10.1016/j.ejmech.2017.04.062 PubMed Abstract | 10.1016/j.ejmech.2017.04.062 | Google Scholar 28500966

[B18] Hankir M. K. Langseder T. Bankoglu E. E. Ghoreishi Y. Dischinger U. Kurlbaum M. (2020). Simulating the post-gastric bypass intestinal microenvironment uncovers a barrier-stabilizing role for FXR. iScience 23 (12), 101777. 10.1016/j.isci.2020.101777 PubMed Abstract | 10.1016/j.isci.2020.101777 | Google Scholar 33294786 PMC7689555

[B19] Hendrick R. E. Monticciolo D. L. Biggs K. W. Malak S. F. (2021). Age distributions of breast cancer diagnosis and mortality by race and ethnicity in US women. Cancer 127 (23), 4384–4392. 10.1002/cncr.33846 PubMed Abstract | 10.1002/cncr.33846 | Google Scholar 34427920

[B20] Hoenke S. Heise N. V. Kahnt M. Deigner H. P. Csuk R. (2020). Betulinic acid derived amides are highly cytotoxic, apoptotic and selective. Eur. J. Med. Chem. 207, 112815. 10.1016/j.ejmech.2020.112815 PubMed Abstract | 10.1016/j.ejmech.2020.112815 | Google Scholar 32956968

[B21] Hsu R. J. Hsu Y. C. Chen S. P. Fu C. L. Yu J. C. Chang F. W. (2015). The triterpenoids of Hibiscus syriacus induce apoptosis and inhibit cell migration in breast cancer cells. BMC Complement. Altern. Med. 15, 65. 10.1186/s12906-015-0592-9 PubMed Abstract | 10.1186/s12906-015-0592-9 | Google Scholar 25885960 PMC4410586

[B22] Khlebnicova T. S. Piven Y. A. Baranovsky A. V. Lakhvich F. A. Shishkina S. V. Zicane D. (2017). Synthesis of novel lupane triterpenoid-indazolone hybrids with oxime ester linkage. Steroids 117, 77–89. 10.1016/j.steroids.2016.08.002 PubMed Abstract | 10.1016/j.steroids.2016.08.002 | Google Scholar 27500691

[B23] Khlebnicova T. S. Piven Y. A. Baranovsky A. V. Lakhvich F. A. Sorokina I. V. Tolstikova T. G. (2019). Fluorine-containing lupane triterpenoid acid derivatives: Design, synthesis and biological evaluation as potential anti-inflammatory agents. Steroids 147, 62–69. 10.1016/j.steroids.2018.10.001 PubMed Abstract | 10.1016/j.steroids.2018.10.001 | Google Scholar 30296549

[B24] Krasutsky P. A. (2006). Birch bark research and development. Nat. Prod. Rep. 23 (6), 919–942. 10.1039/b606816b PubMed Abstract | 10.1039/b606816b | Google Scholar 17119640

[B25] Liu J. C. Narva S. Zhou K. Zhang W. (2019). A review on the antitumor activity of various nitrogenous-based heterocyclic compounds as NSCLC inhibitors. Mini Rev. Med. Chem. 19 (18), 1517–1530. 10.2174/1389557519666190312152358 PubMed Abstract | 10.2174/1389557519666190312152358 | Google Scholar 30864519

[B26] Martin-Blazquez A. Diaz C. Gonzalez-Flores E. Franco-Rivas D. Jimenez-Luna C. Melguizo C. (2019). Untargeted LC-HRMS-based metabolomics to identify novel biomarkers of metastatic colorectal cancer. Sci. Rep. 9 (1). 10.1038/s41598-019-55952-8 PubMed Abstract | 10.1038/s41598-019-55952-8 | Google Scholar PMC6934557 31882610

[B27] Matsubara T. Li F. Gonzalez F. J. (2013). FXR signaling in the enterohepatic system. Mol. Cell. Endocrinol. 368 (1-2), 17–29. 10.1016/j.mce.2012.05.004 PubMed Abstract | 10.1016/j.mce.2012.05.004 | Google Scholar 22609541 PMC3491147

[B28] Maughan K. L. Lutterbie M. A. Ham P. S. (2010). Treatment of breast cancer. Am. Fam. Physician 81 (11), 1339–1346. PubMed Abstract | Google Scholar 20521754

[B29] Morettin A. Baldwin R. M. Cote J. (2015). Arginine methyltransferases as novel therapeutic targets for breast cancer. Mutagenesis 30 (2), 177–189. 10.1093/mutage/geu039 PubMed Abstract | 10.1093/mutage/geu039 | Google Scholar 25688111

[B30] Morris S. M. Jr. (2007). Arginine metabolism: Boundaries of our knowledge. J. Nutr. 137 (6), 1602S–1609S. 10.1093/jn/137.6.1602S PubMed Abstract | 10.1093/jn/137.6.1602S | Google Scholar 17513435

[B31] Mzhelskaya M. M. Klinnikova M. G. Koldysheva E. V. Lushnikova E. L. (2017). Expression of flk-1 and cyclin D2 mRNA in the myocardium of rats with doxorubicin-induced cardiomyopathy and after treatment with betulonic acid amide. Bull. Exp. Biol. Med. 163 (6), 809–813. 10.1007/s10517-017-3909-5 PubMed Abstract | 10.1007/s10517-017-3909-5 | Google Scholar 29063324

[B32] Nasreddine G. El-Sibai M. Abi-Habib R. J. (2020). Cytotoxicity of [HuArgI (co)-PEG5000]-induced arginine deprivation to ovarian Cancer cells is autophagy dependent. Invest.. New Drugs 38 (1), 10–19. 10.1007/s10637-019-00756-w PubMed Abstract | 10.1007/s10637-019-00756-w | Google Scholar 30887252

[B33] Pang Z. Chong J. Zhou G. de Lima Morais D. A. Chang L. Barrette M. (2021). MetaboAnalyst 5.0: Narrowing the gap between raw spectra and functional insights. Nucleic Acids Res. 49 (W1), W388–W396. 10.1093/nar/gkab382 PubMed Abstract | 10.1093/nar/gkab382 | Google Scholar 34019663 PMC8265181

[B34] Popov S. A. Semenova M. D. Baev D. S. Sorokina I. V. Zhukova N. A. Frolova T. S. (2019). Lupane-type conjugates with aminoacids, 1, 3, 4- oxadiazole and 1, 2, 5-oxadiazole-2-oxide derivatives: Synthesis, anti-inflammatory activity and *in silico* evaluation of target affinity. Steroids 150, 108443. 10.1016/j.steroids.2019.108443 PubMed Abstract | 10.1016/j.steroids.2019.108443 | Google Scholar 31295462

[B35] Rath M. Muller I. Kropf P. Closs E. I. Munder M. (2014). Metabolism via arginase or nitric oxide synthase: Two competing arginine pathways in macrophages. Front. Immunol. 5, 532. 10.3389/fimmu.2014.00532 PubMed Abstract | 10.3389/fimmu.2014.00532 | Google Scholar 25386178 PMC4209874

[B36] Saxena B. B. Zhu L. Hao M. Kisilis E. Katdare M. Oktem O. (2006). Boc-lysinated-betulonic acid: A potent, anti-prostate cancer agent. Bioorg. Med. Chem. 14 (18), 6349–6358. 10.1016/j.bmc.2006.05.048 PubMed Abstract | 10.1016/j.bmc.2006.05.048 | Google Scholar 16777417

[B37] Seo Y. Lee J. H. Park S. H. Namkung W. Kim I. (2020). Expansion of chemical space based on a pyrrolo[1, 2-a]pyrazine core: Synthesis and its anticancer activity in prostate cancer and breast cancer cells. Eur. J. Med. Chem. 188, 111988. 10.1016/j.ejmech.2019.111988 PubMed Abstract | 10.1016/j.ejmech.2019.111988 | Google Scholar 31901746

[B38] Shu Y. He D. Li W. Wang M. Zhao S. Liu L. (2020). Hepatoprotective effect of citrus aurantium L. Against APAP-induced liver injury by regulating liver lipid metabolism and apoptosis. Int. J. Biol. Sci. 16 (5), 752–765. 10.7150/ijbs.40612 PubMed Abstract | 10.7150/ijbs.40612 | Google Scholar 32071546 PMC7019131

[B39] Sousa J. L. C. Freire C. S. R. Silvestre A. J. D. Silva A. M. S. (2019). Recent developments in the functionalization of betulinic acid and its natural analogues: A route to new bioactive compounds. Molecules 24 (2), 355. 10.3390/molecules24020355 10.3390/molecules24020355 | Google Scholar PMC6359067 30669472

[B40] Sun L. Cai J. Gonzalez F. J. (2021). The role of farnesoid X receptor in metabolic diseases, and gastrointestinal and liver cancer. Nat. Rev. Gastroenterol. Hepatol. 18 (5), 335–347. 10.1038/s41575-020-00404-2 PubMed Abstract | 10.1038/s41575-020-00404-2 | Google Scholar 33568795

[B41] Szefel J. Danielak A. Kruszewski W. J. (2019). Metabolic pathways of L-arginine and therapeutic consequences in tumors. Adv. Med. Sci. 64 (1), 104–110. 10.1016/j.advms.2018.08.018 PubMed Abstract | 10.1016/j.advms.2018.08.018 | Google Scholar 30605863

[B42] Taglieri L. Saccoliti F. Nicolai A. Peruzzi G. Madia V. N. Tudino V. (2020). Discovery of a pyrimidine compound endowed with antitumor activity. Invest.. New Drugs 38 (1), 39–49. 10.1007/s10637-019-00762-y PubMed Abstract | 10.1007/s10637-019-00762-y | Google Scholar 30900116

[B43] Wang H. Zhang W. Cheng Y. Zhang X. Xue N. Wu G. (2018a). Design, synthesis and biological evaluation of ligustrazine-flavonoid derivatives as potential anti-tumor agents. Molecules 23 (9), 2187. 10.3390/molecules23092187 10.3390/molecules23092187 | Google Scholar PMC6225232 30200208

[B44] Wang P. Zhao R. Yan W. Zhang X. Zhang H. Xu B. (2018b). Neuroprotection by new ligustrazine-cinnamon acid derivatives on CoCl2-induced apoptosis in differentiated PC12 cells. Bioorg. Chem. 77, 360–369. 10.1016/j.bioorg.2018.01.029 PubMed Abstract | 10.1016/j.bioorg.2018.01.029 | Google Scholar 29421712

[B45] Xu B. Chu F. Zhang Y. Wang X. Li Q. Liu W. (2015). A series of new ligustrazine-triterpenes derivatives as anti-tumor agents: Design, synthesis, and biological evaluation. Int. J. Mol. Sci. 16 (9), 21035–21055. 10.3390/ijms160921035 PubMed Abstract | 10.3390/ijms160921035 | Google Scholar 26404253 PMC4613240

[B46] Xu B. Yan W. Q. Xu X. Wu G. R. Zhang C. Z. Han Y. T. (2017). Combination of amino acid/dipeptide with ligustrazine-betulinic acid as antitumor agents. Eur. J. Med. Chem. 130, 26–38. 10.1016/j.ejmech.2017.02.036 PubMed Abstract | 10.1016/j.ejmech.2017.02.036 | Google Scholar 28237794

[B47] Yang S. J. Liu M. C. Zhao Q. Hu D. Y. Xue W. Yang S. (2015). Synthesis and biological evaluation of betulonic acid derivatives as antitumor agents. Eur. J. Med. Chem. 96, 58–65. 10.1016/j.ejmech.2015.04.006 PubMed Abstract | 10.1016/j.ejmech.2015.04.006 | Google Scholar 25874331

[B48] Yang Y. Xie T. Tian X. Han N. Liu X. Chen H. (2020). Betulinic acid-nitrogen heterocyclic derivatives: Design, synthesis, and antitumor evaluation *in vitro* . Molecules 25 (4), 948. 10.3390/molecules25040948 PubMed Abstract | 10.3390/molecules25040948 | Google Scholar PMC7070564 32093264

[B49] Yu D. D. Lin W. Forman B. M. Chen T. (2014). Identification of trisubstituted-pyrazol carboxamide analogs as novel and potent antagonists of farnesoid X receptor. Bioorg. Med. Chem. 22 (11), 2919–2938. 10.1016/j.bmc.2014.04.014 PubMed Abstract | 10.1016/j.bmc.2014.04.014 | Google Scholar 24775917 PMC4147378

[B50] Zhang H. Zhu P. Liu J. Yang X. Xu S. Yao H. (2014). Synthesis and antitumor activity of novel 3-oxo-23-hydroxybetulinic acid derivatives. Eur. J. Med. Chem. 87, 159–167. 10.1016/j.ejmech.2014.09.058 PubMed Abstract | 10.1016/j.ejmech.2014.09.058 | Google Scholar 25247772

[B51] Zunica E. R. M. Axelrod C. L. Cho E. Spielmann G. Davuluri G. Alexopoulos S. J. (2021). Breast cancer growth and proliferation is suppressed by the mitochondrial targeted furazano[3, 4-b]pyrazine BAM15. Cancer Metab. 9 (1), 36. 10.1186/s40170-021-00274-5 PubMed Abstract | 10.1186/s40170-021-00274-5 | Google Scholar 34627389 PMC8502397

