# Supplementary material for: Design, synthesis and cytotoxic evaluation of novel betulonic acid-diazine derivatives as potential antitumor agents
Source: Front Chem. 2022 Sep 6;10:969770. doi: 10.3389/fchem.2022.969770 (PMC9486541; doi:10.3389/fchem.2022.969770)
Supplement: Supplementary file 1 [file DataSheet1.PDF]

Design, synthesis and cytotoxic evaluation of novel betulonic acid-diazine derivatives as potential antitumor agents

Yisong Shu[1†], Feifei Li[1†], Yaotian Han[1], Penglong Wang[1], Feng Gao[1], Mengmeng Yan[1], Miao Liang, Qiang Ma[3*], Yuzhong Zhang[1*], Xia Ding[2*], Haimin Lei[1*]

[†]These authors have contributed equally to this work and share first authorship

[1]School of Chinese Materia Medica, Beijing University of Chinese Medicine, Beijing, China,
[2]School of Traditional Chinese Medicine, Beijing University of Chinese Medicine, Beijing, China
[3]Chinese Academy of Inspection and Quarantine, Beijing, China

*** Correspondence:**
Qiang Ma
maqiang@caiq.org.cn

Yuzhong Zhang
zyz100102@126.com

Xia Ding
dingx@bucm.edu.cn

Haimin Lei
hm_lei@126.com

Supplemental materials

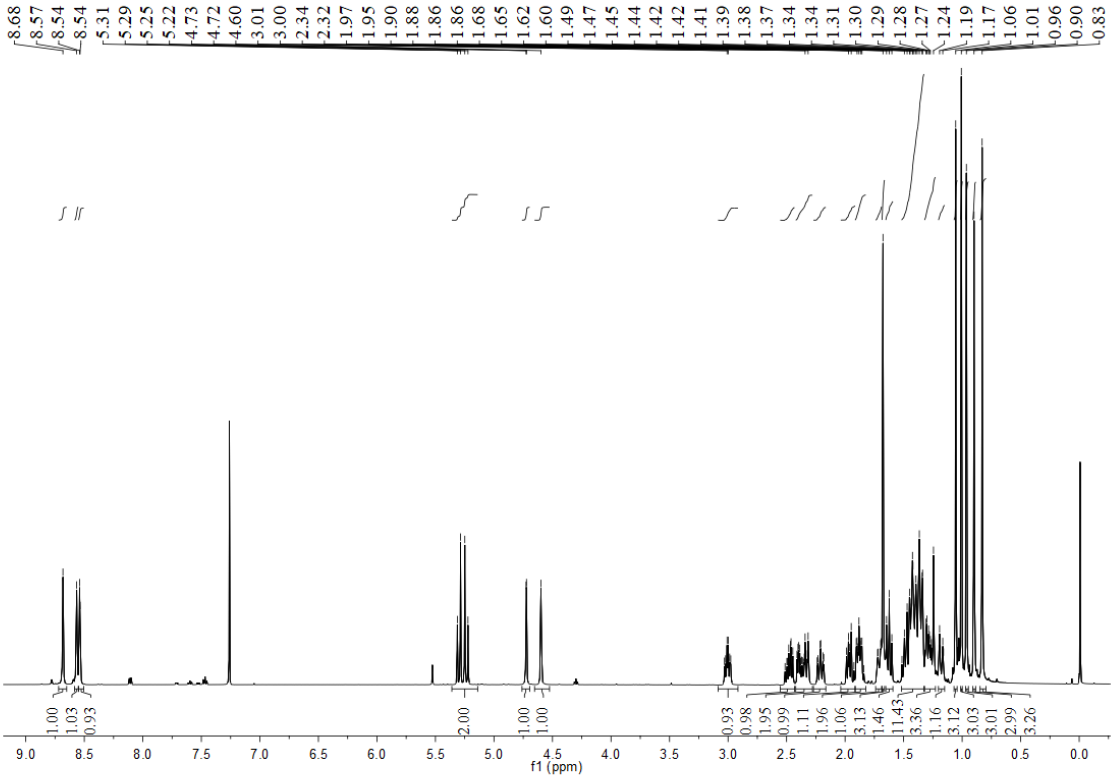

BoAA ^{1}H-NMR ($CDCl_3$)

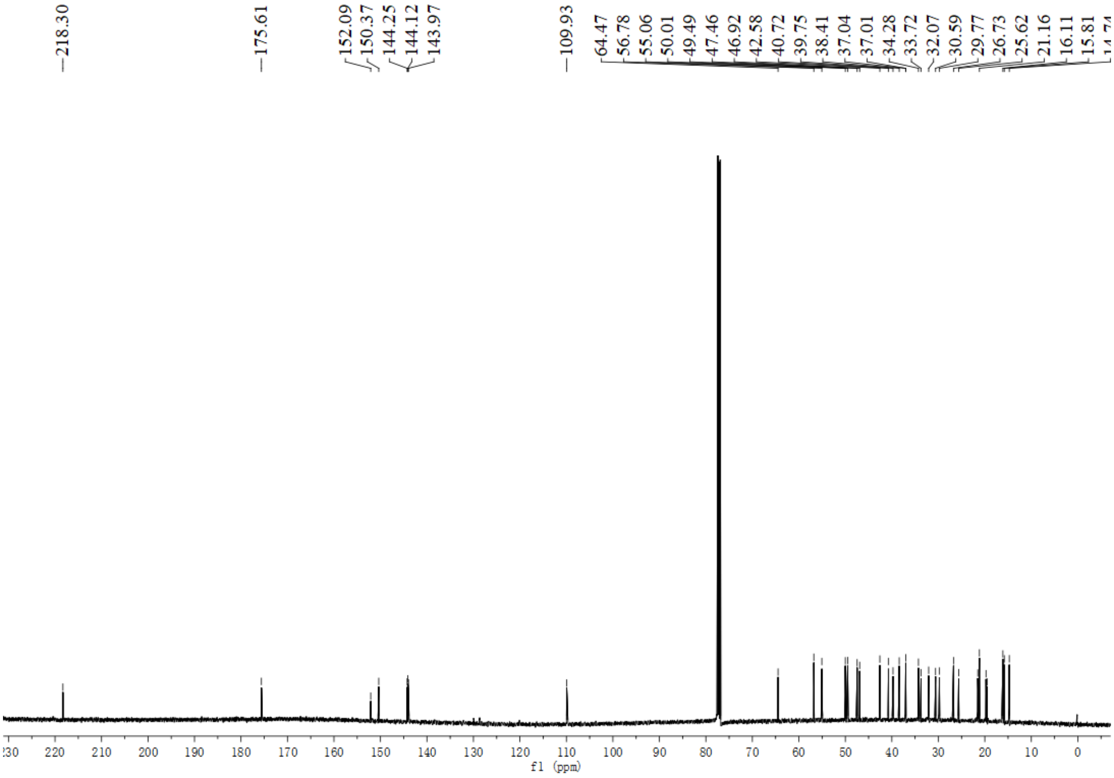

BoAA ^{13}C-NMR ($CDCl_3$)

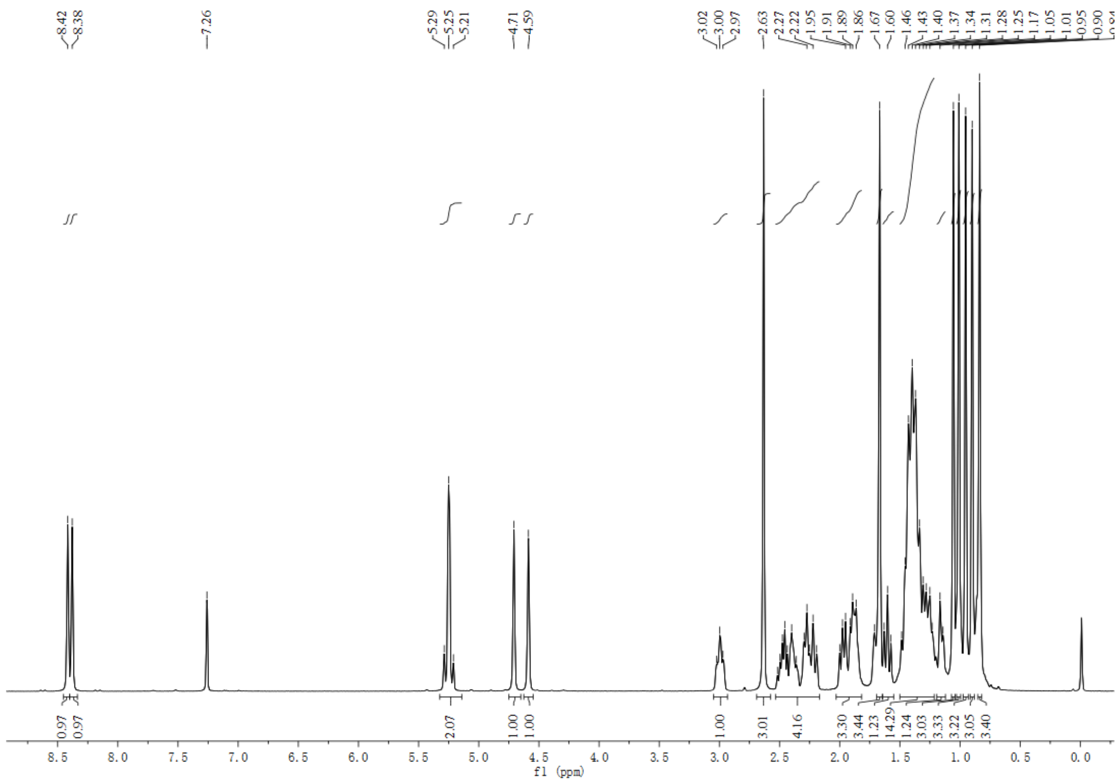

BoAB ^{1}H-NMR ($CDCl_3$)

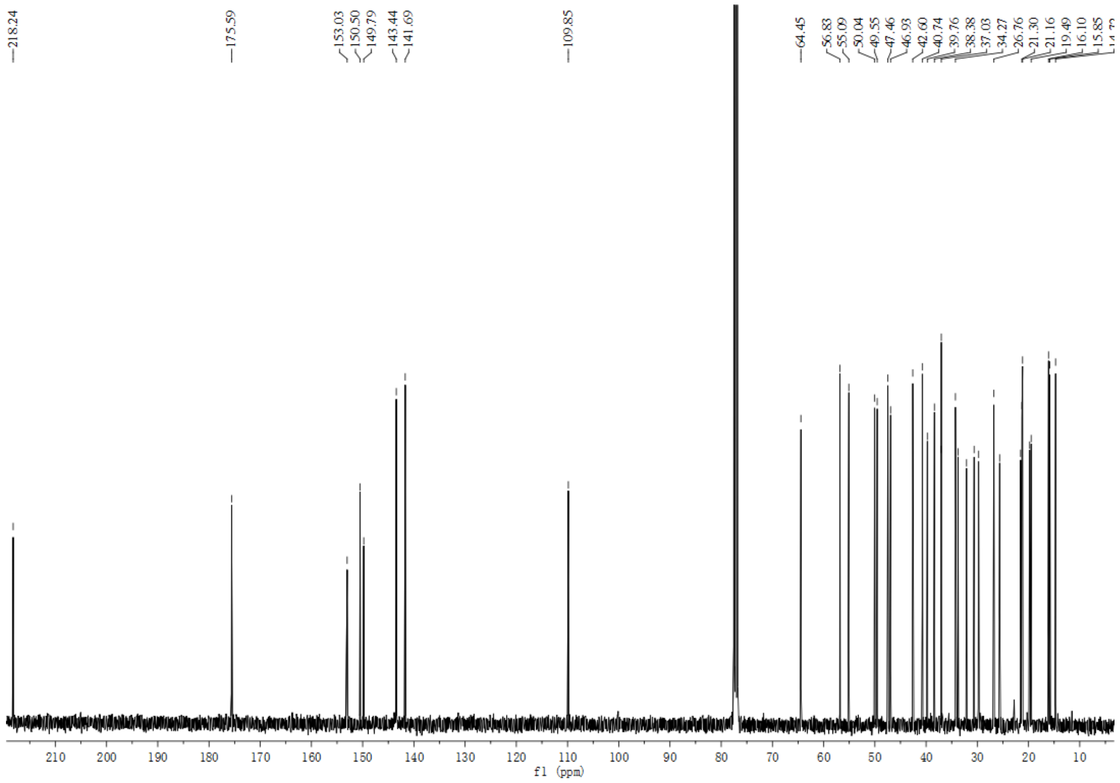

BoAB ^{13}C-NMR($CDCl_3$)

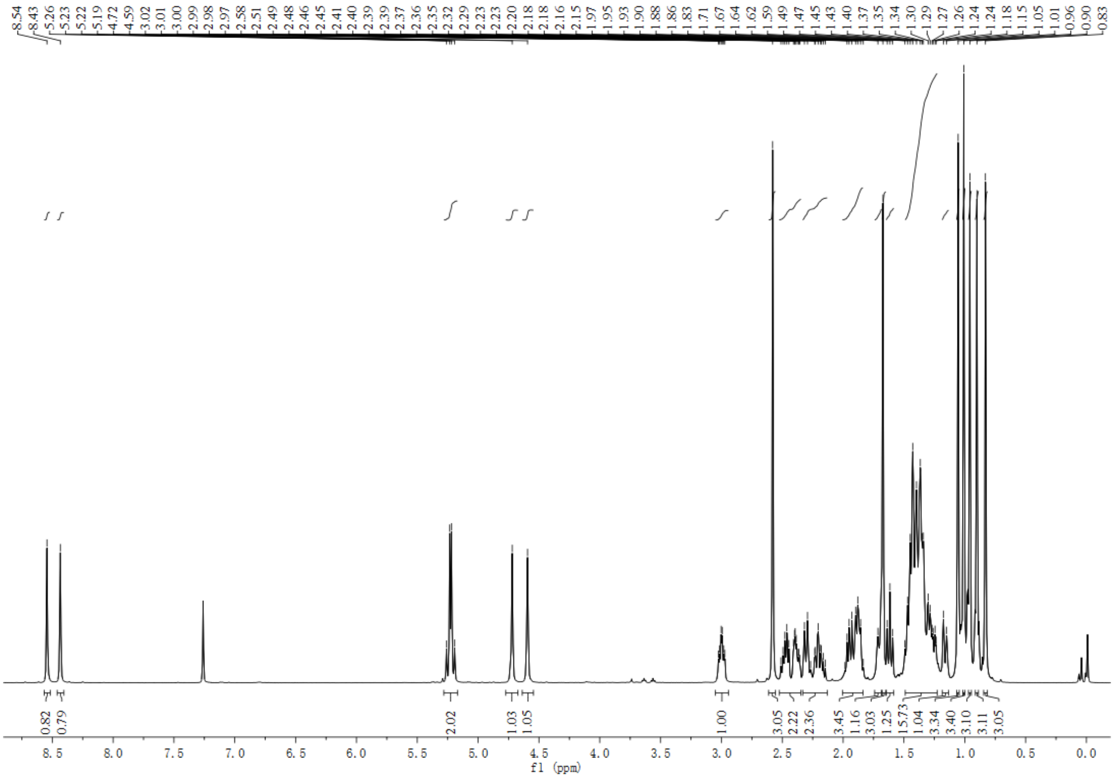

BoAC ^{1}**H-NMR** (CDCl$_3$)

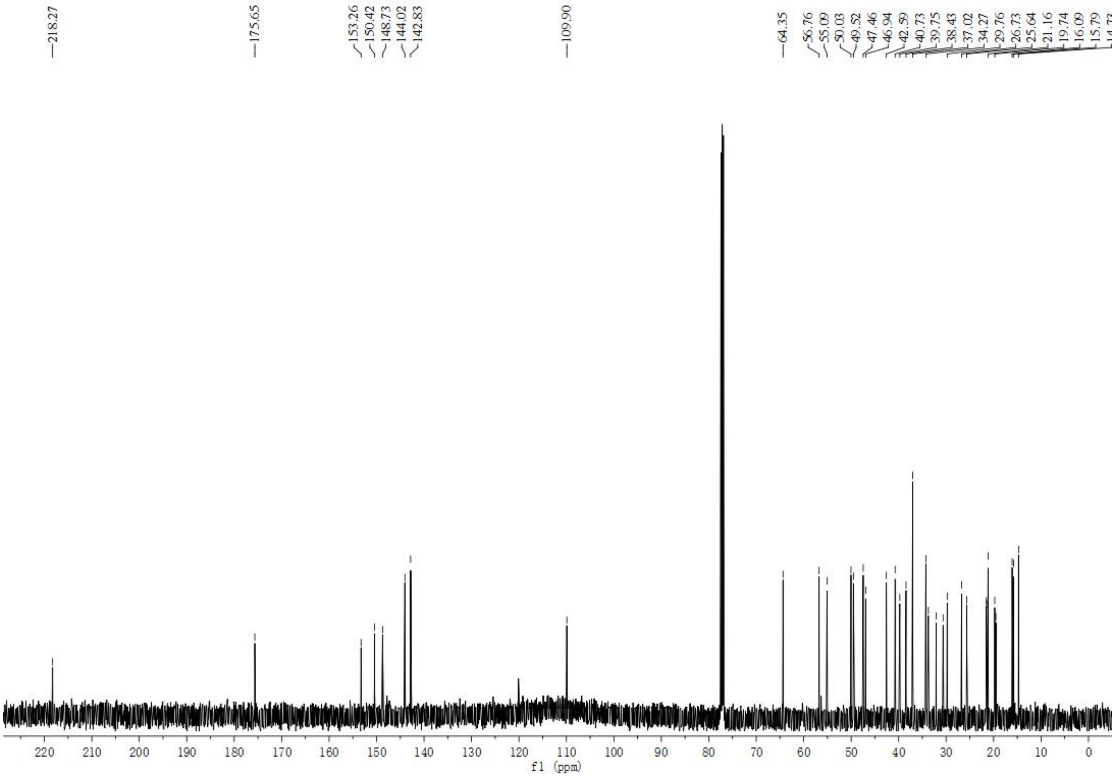

BoAC ^{13}C-NMR (CDCl$_3$)

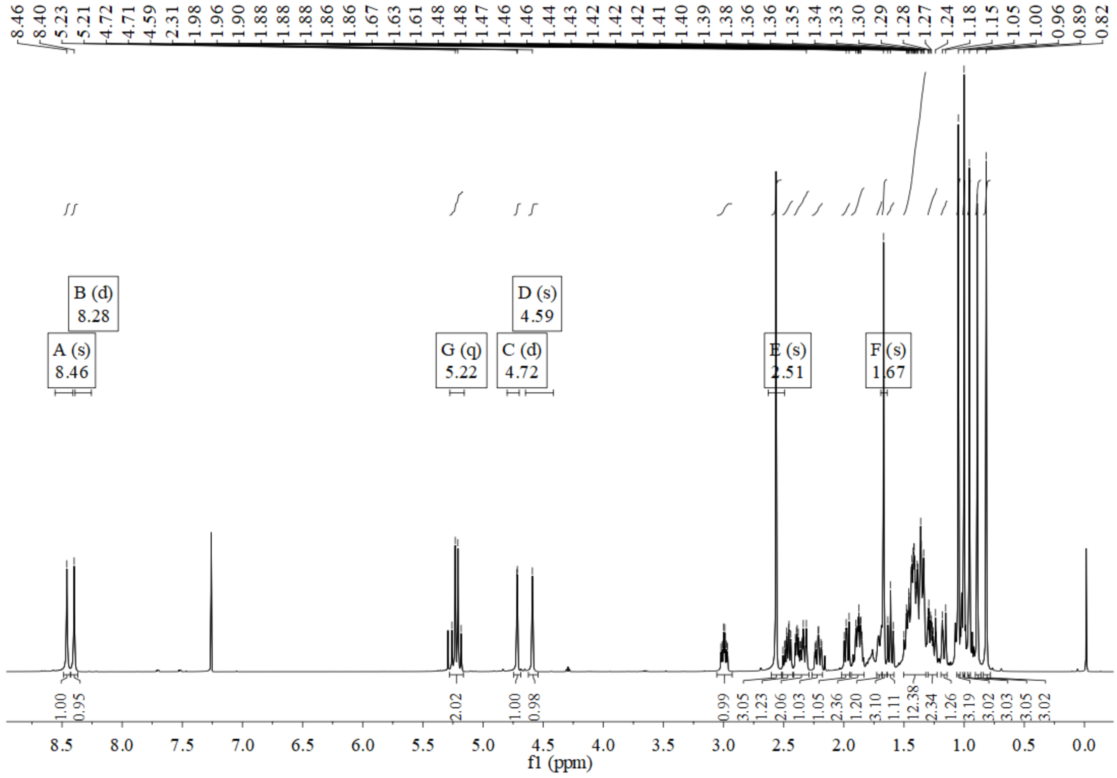

BoAD ^{1}H-NMR ($CDCl_3$)

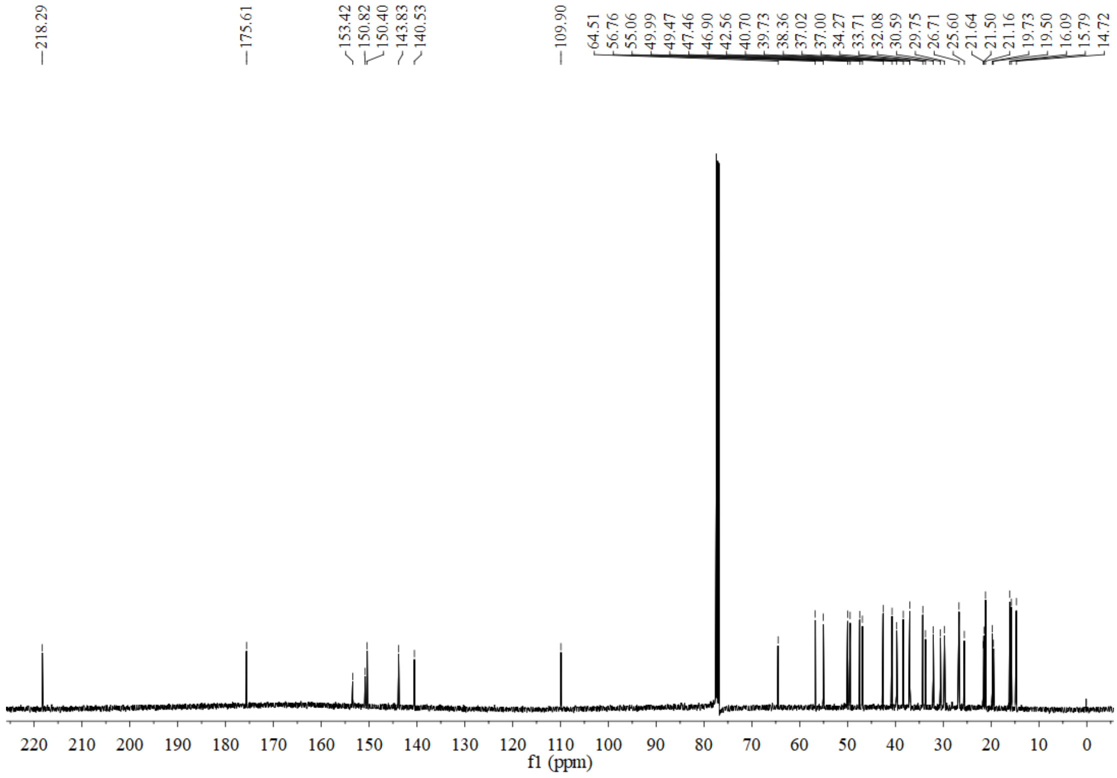

BoAD ^{13}C-NMR($CDCl_3$)

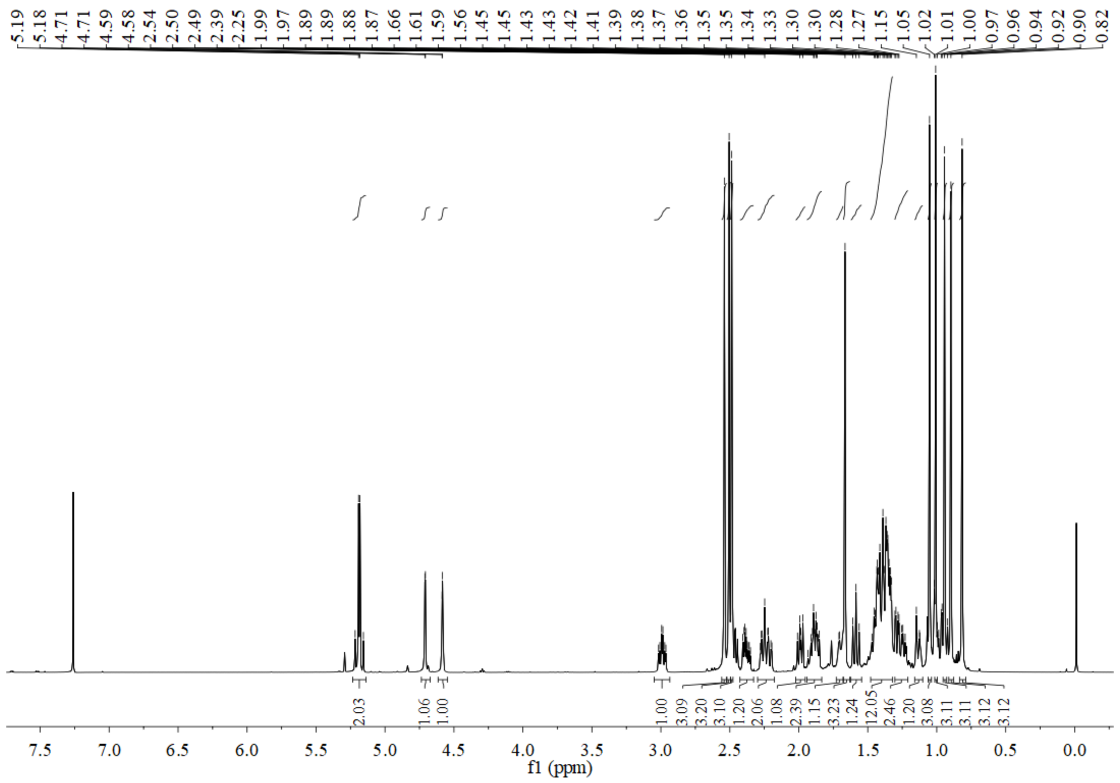

BoAE ^{1}H-NMR ($CDCl_3$)

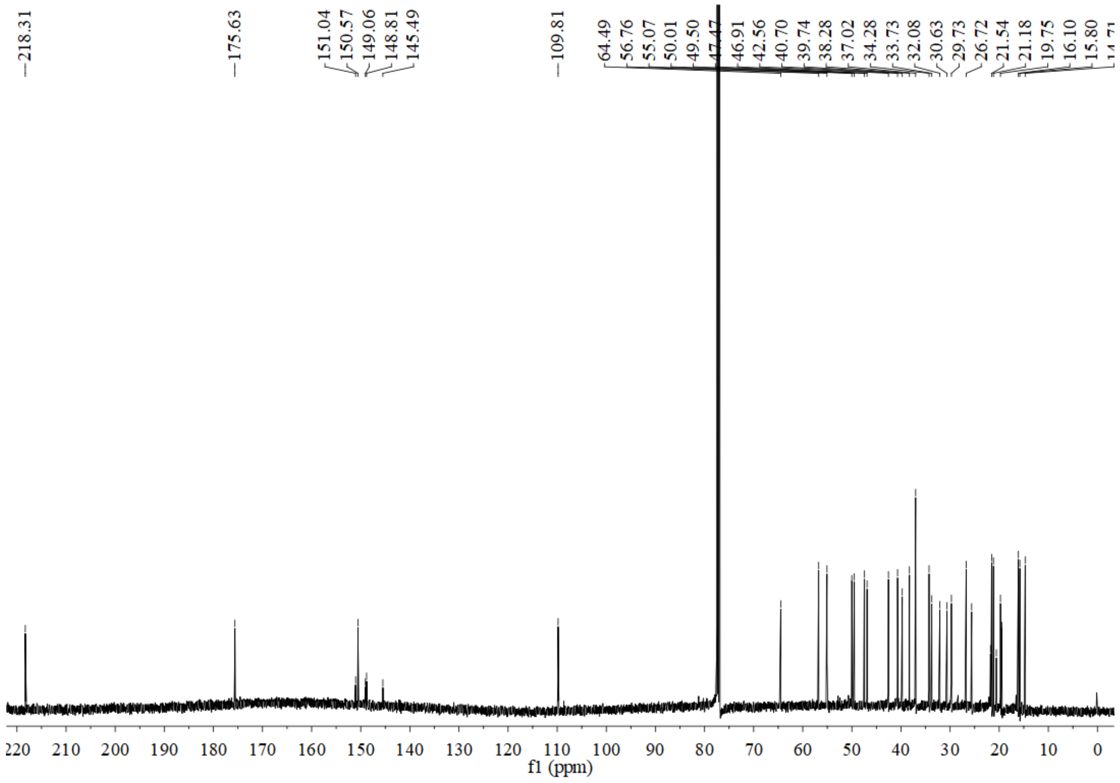

BoAE ^{13}C-NMR ($CDCl_3$)

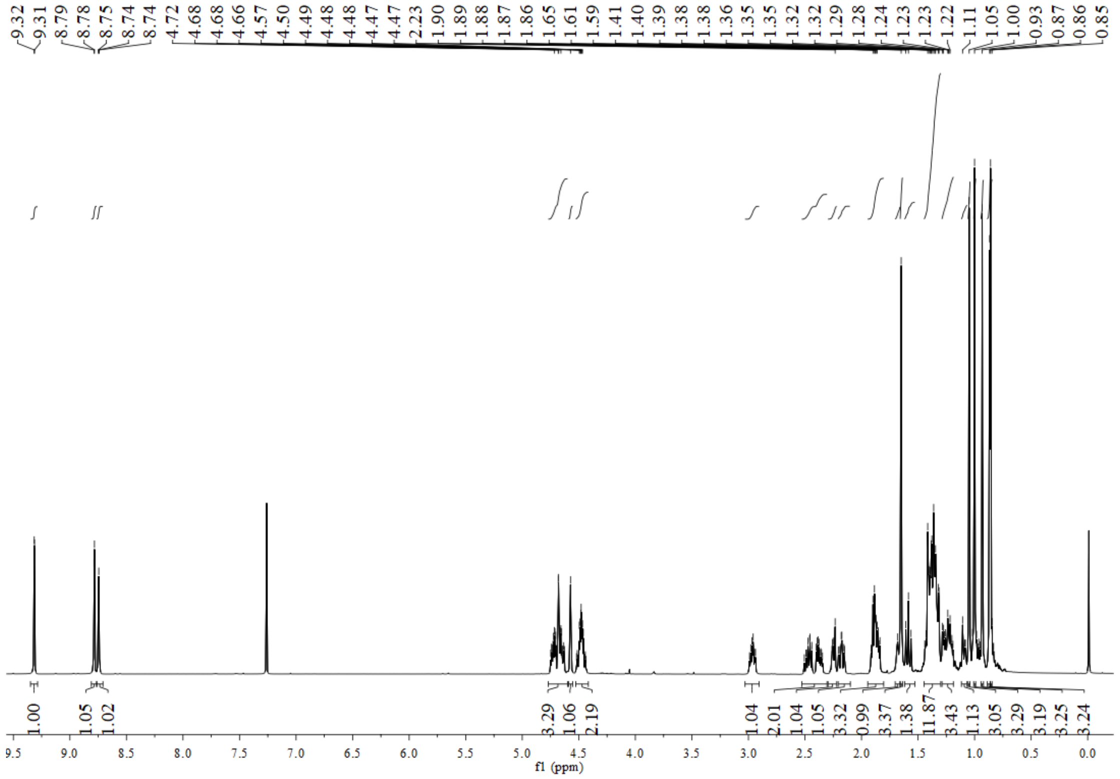

BoA2A ^{1}H-NMR ($CDCl_3$)

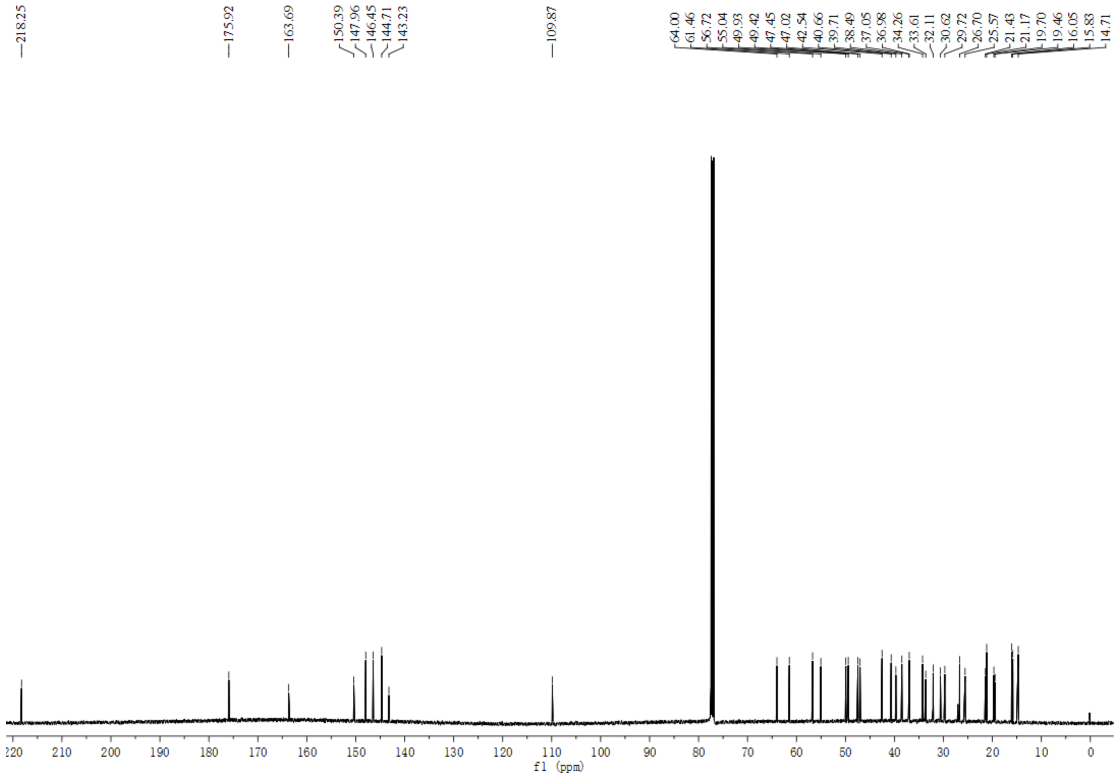

BoA2A ^{13}C-NMR ($CDCl_3$)

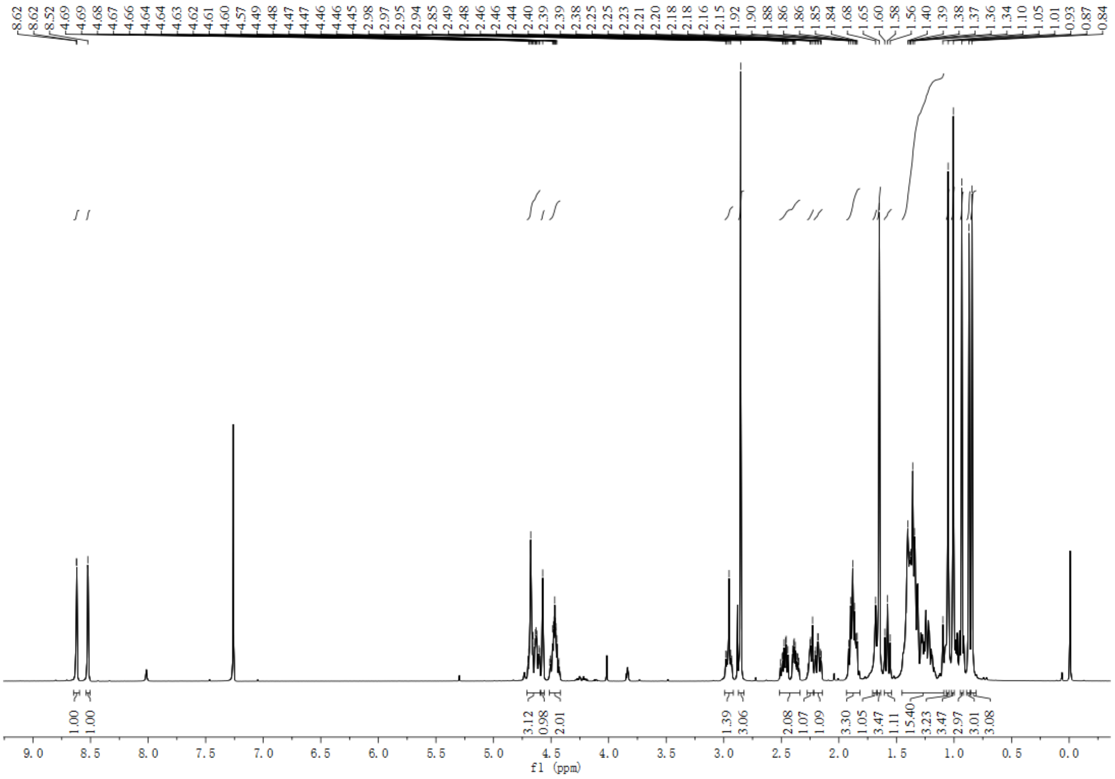

BoA2B ^{1}H-NMR ($CDCl_3$)

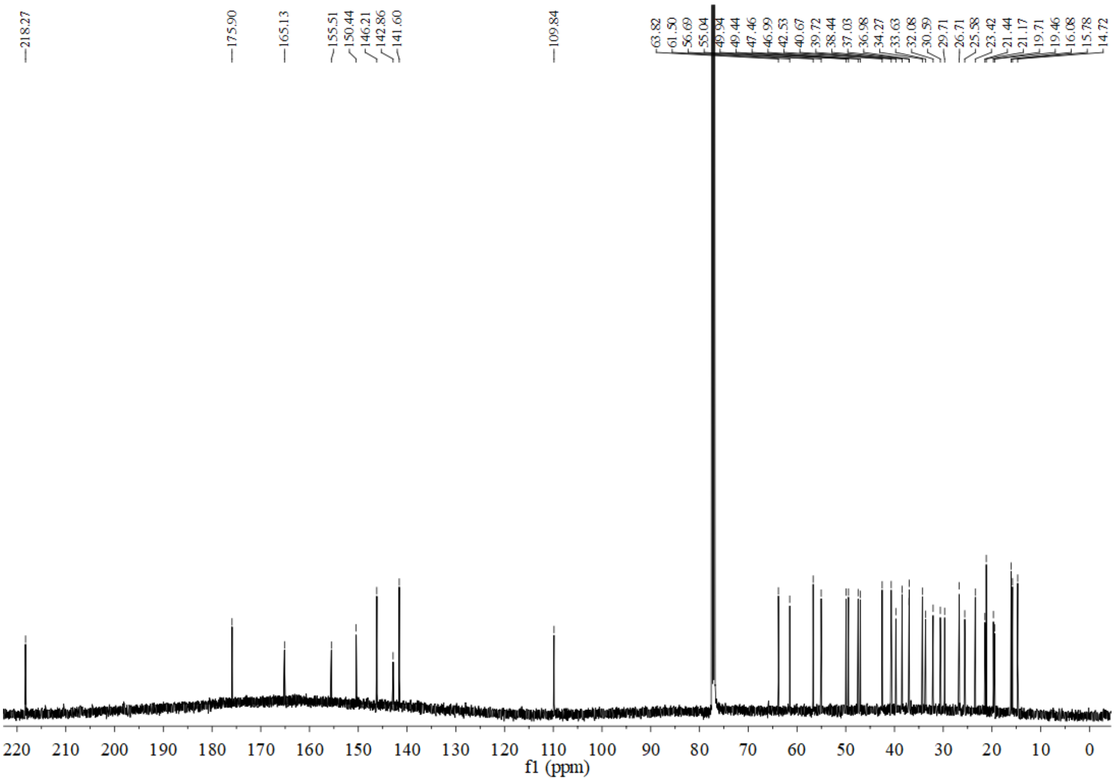

BoA2B ^{13}C-NMR ($CDCl_3$)

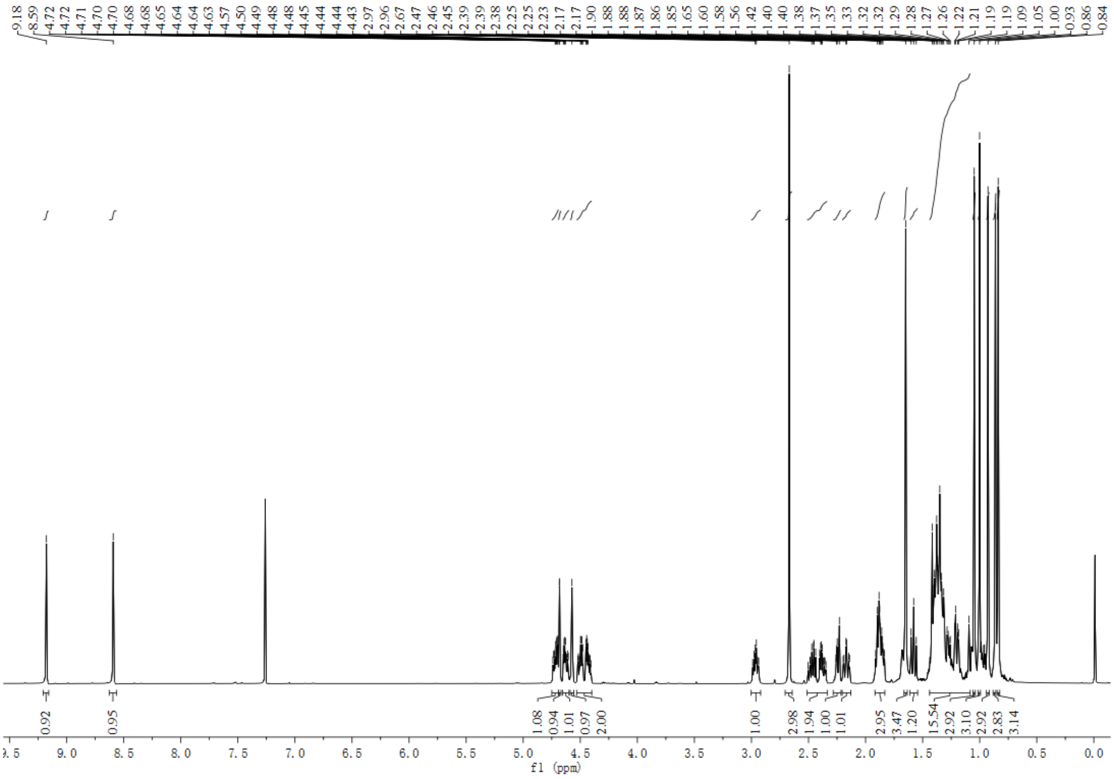

BoA2C ^{1}H-NMR ($CDCl_3$)

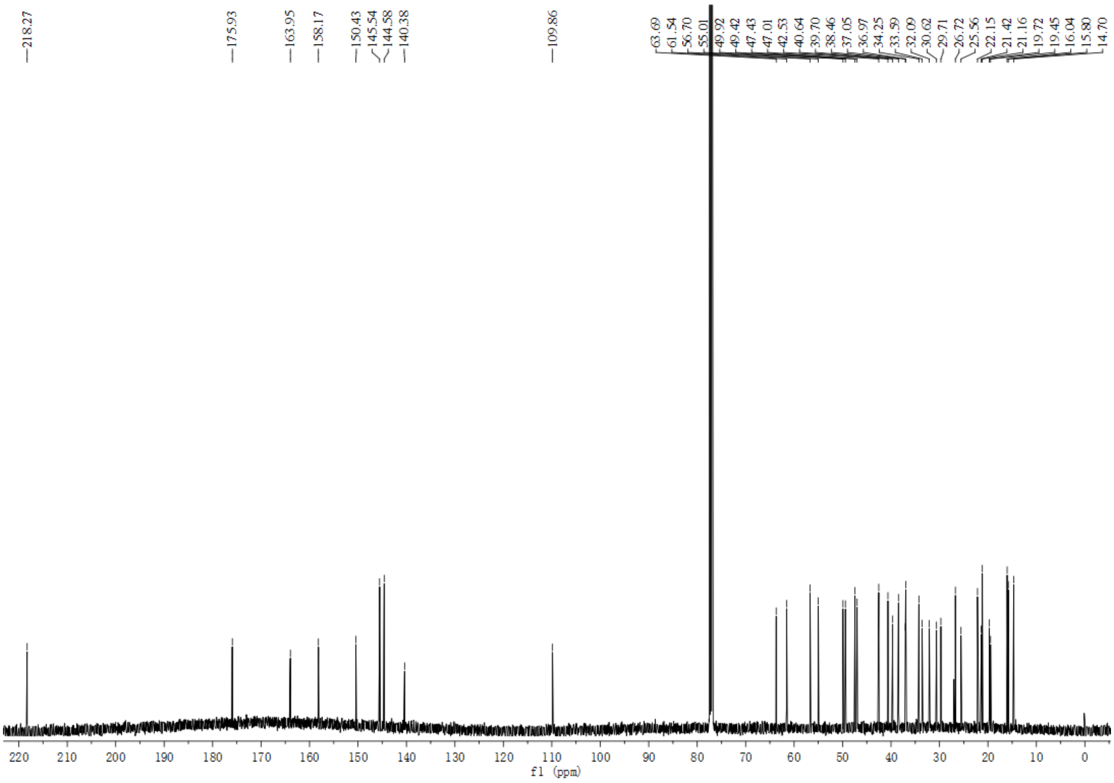

BoA2C ^{13}C-NMR ($CDCl_3$)

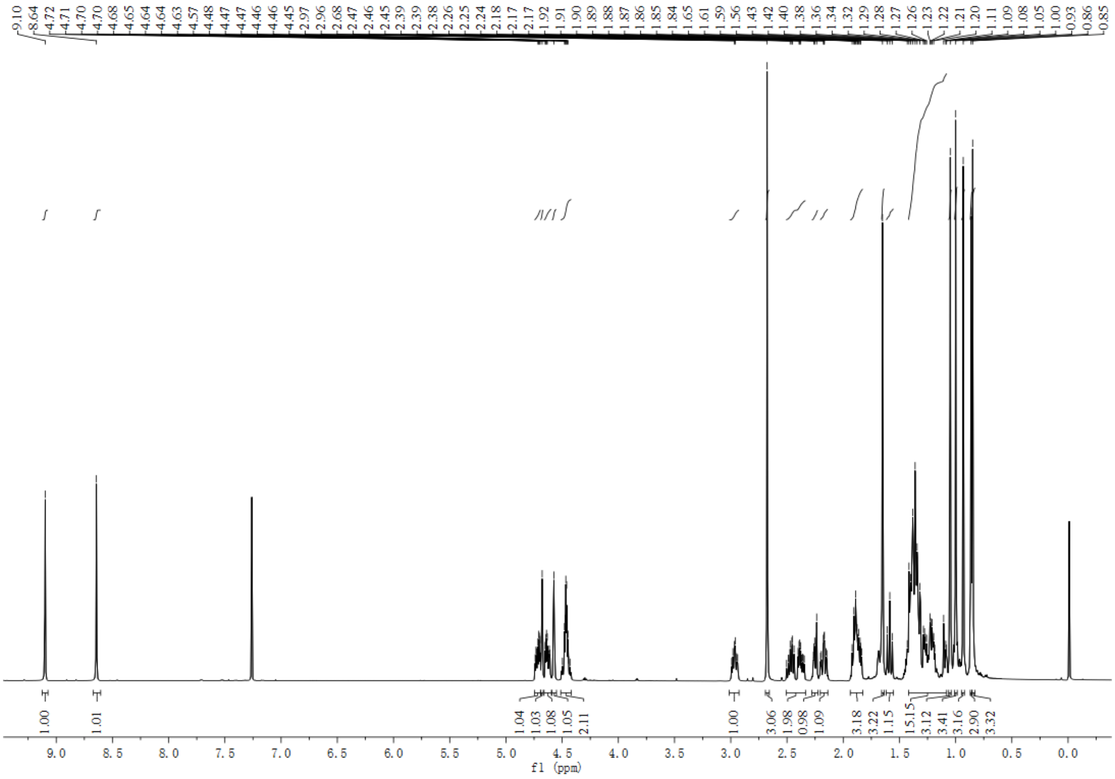

BoA2D ^{1}H-NMR ($CDCl_3$)

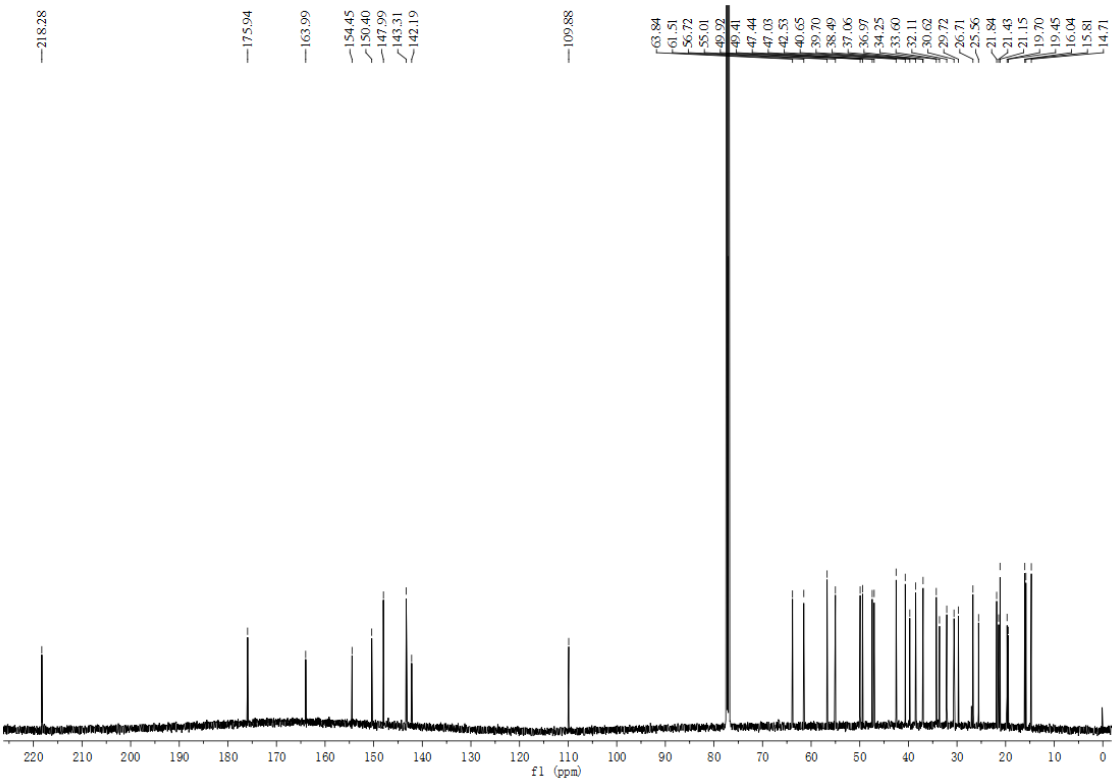

BoA2D ^{13}C-NMR ($CDCl_3$)

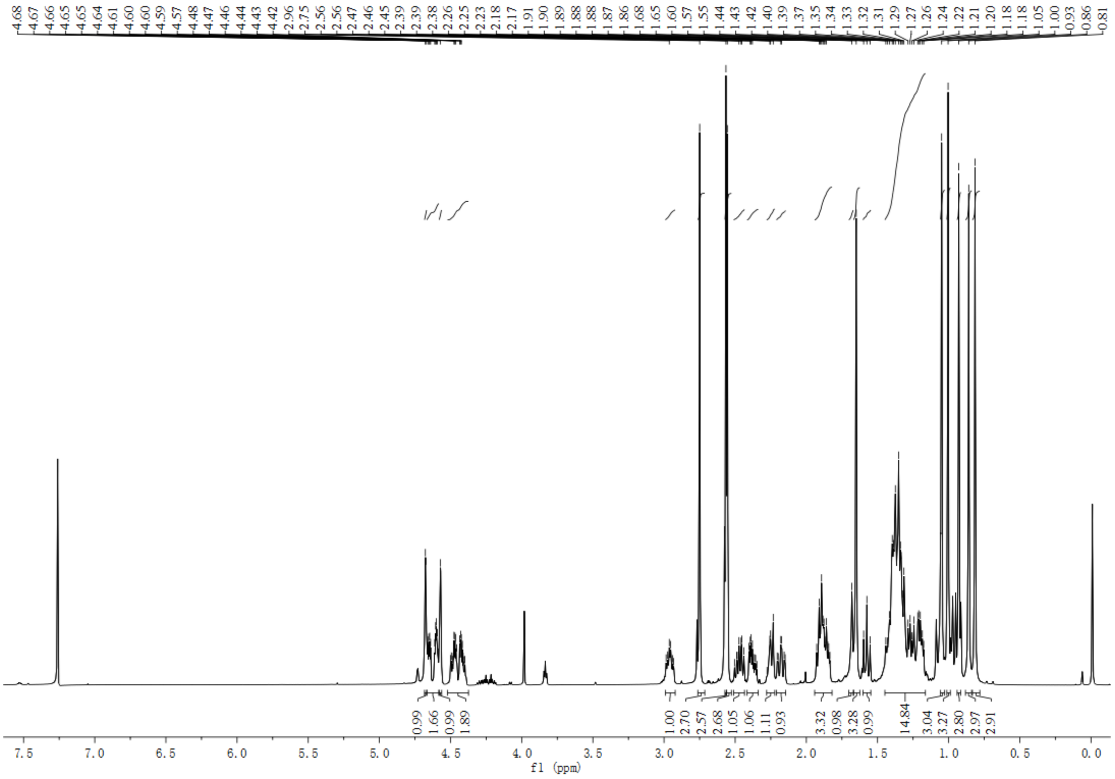

BoA2E ^{1}H-NMR ($CDCl_3$)

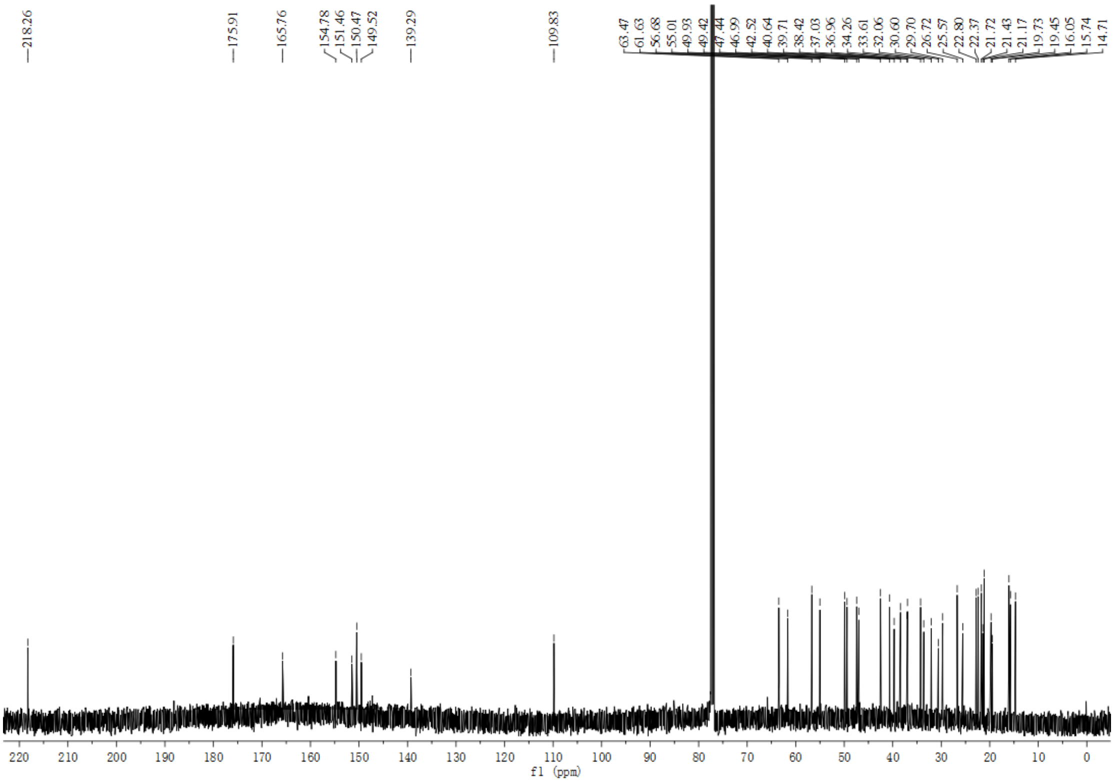

BoA2E ^{13}C-NMR ($CDCl_3$)

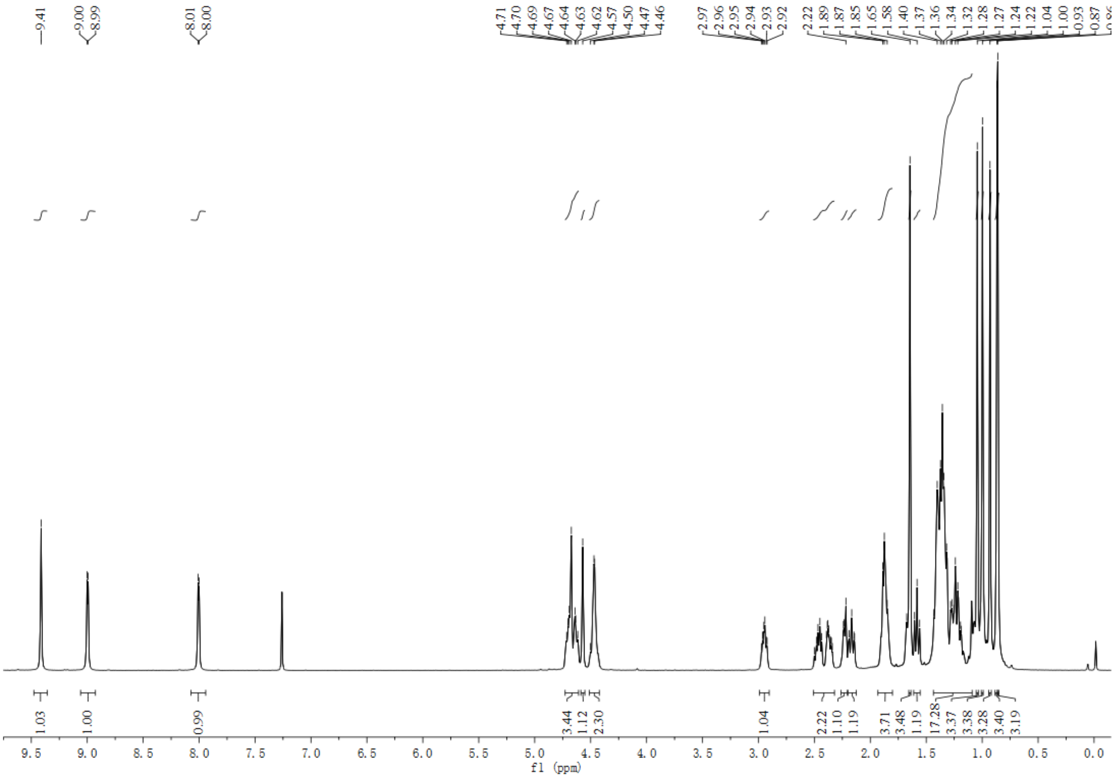

BoA2F ^{1}H-NMR ($CDCl_3$)

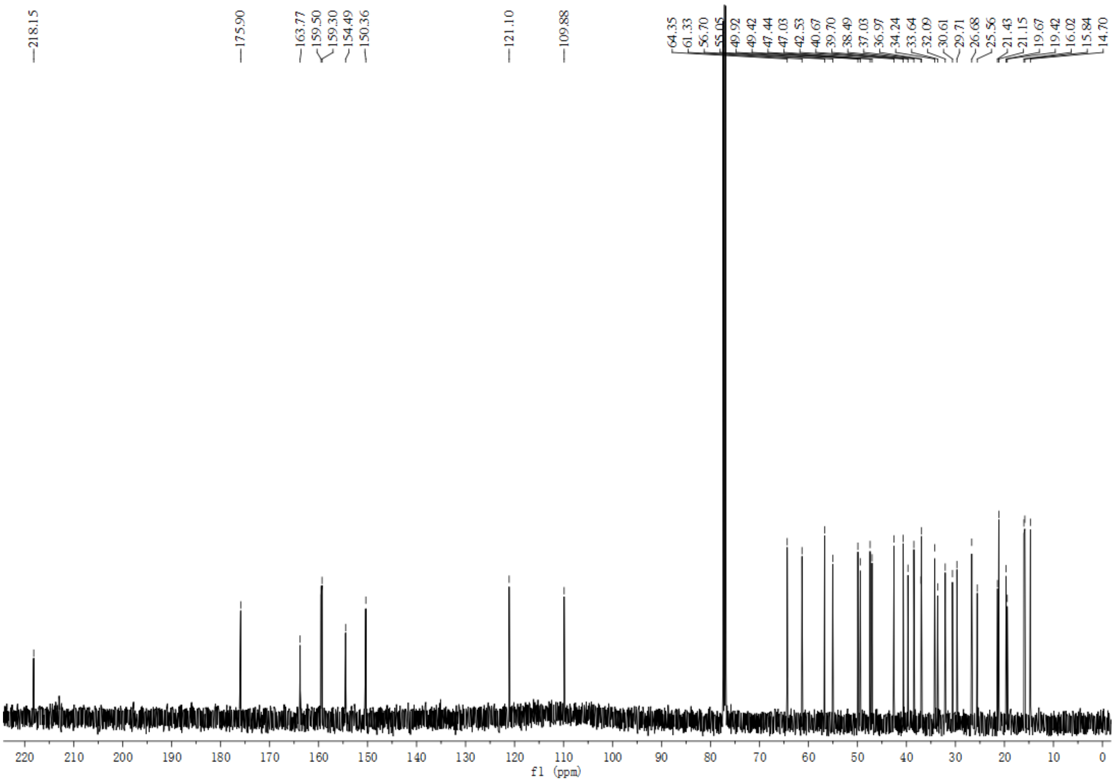

BoA2F ^{13}C-NMR ($CDCl_3$)

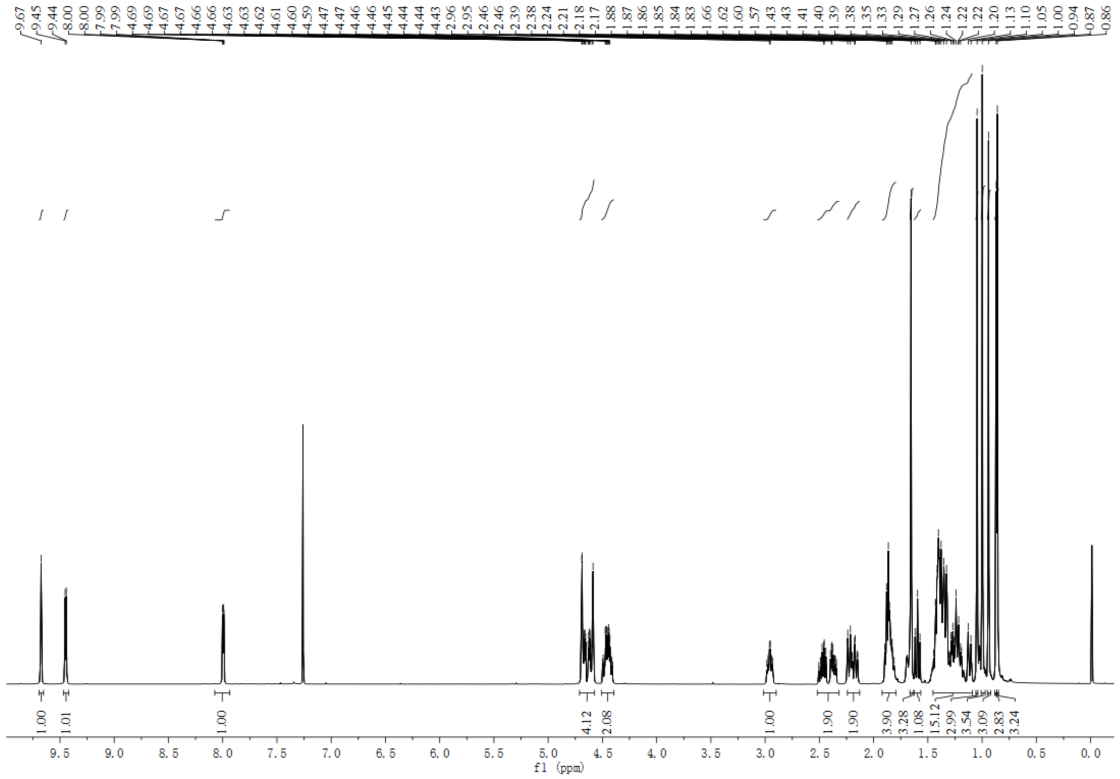

BoA2G ^{1}H-NMR ($CDCl_3$)

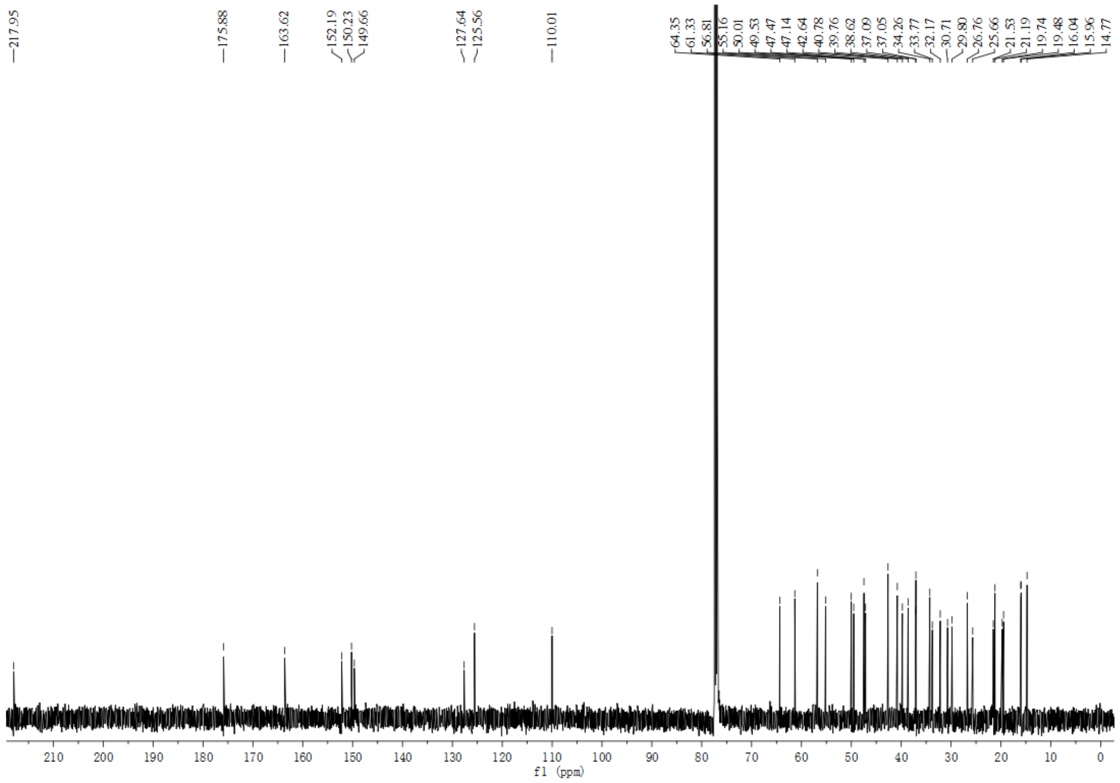

BoA2G ^{13}C-NMR ($CDCl_3$)

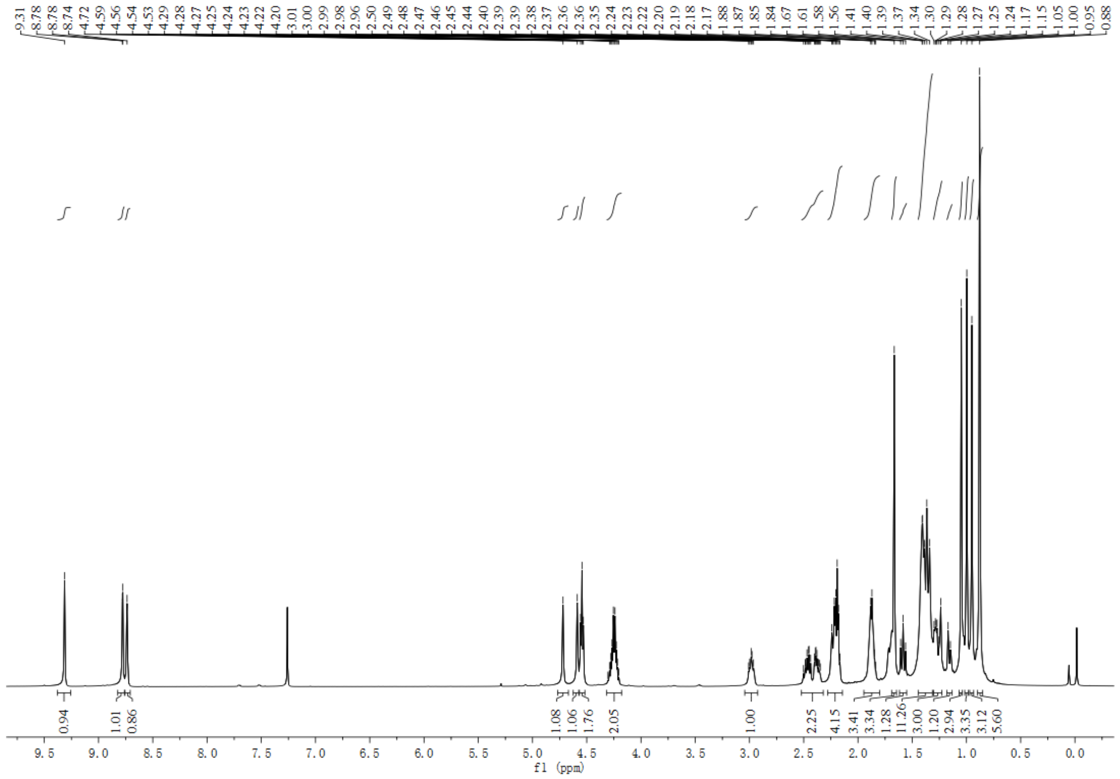

BoA3A ^{1}H-NMR ($CDCl_3$)

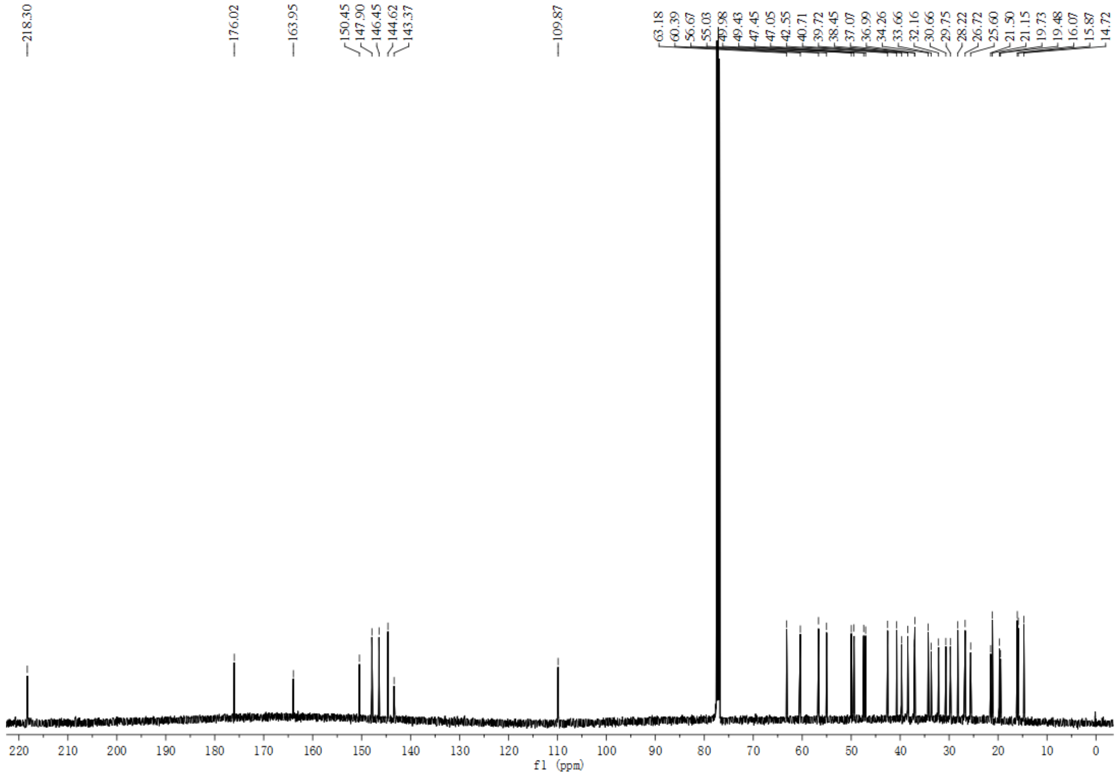

BoA3A ^{13}C-NMR ($CDCl_3$)

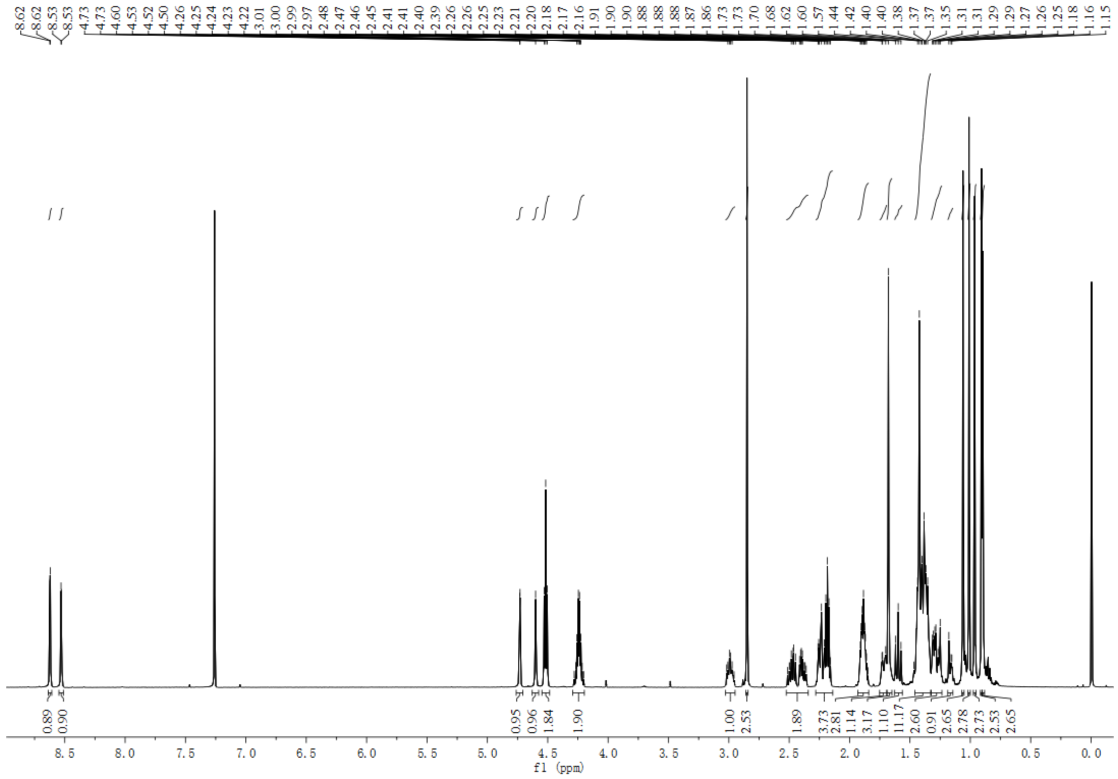

BoA3B ^{1}H-NMR ($CDCl_3$)

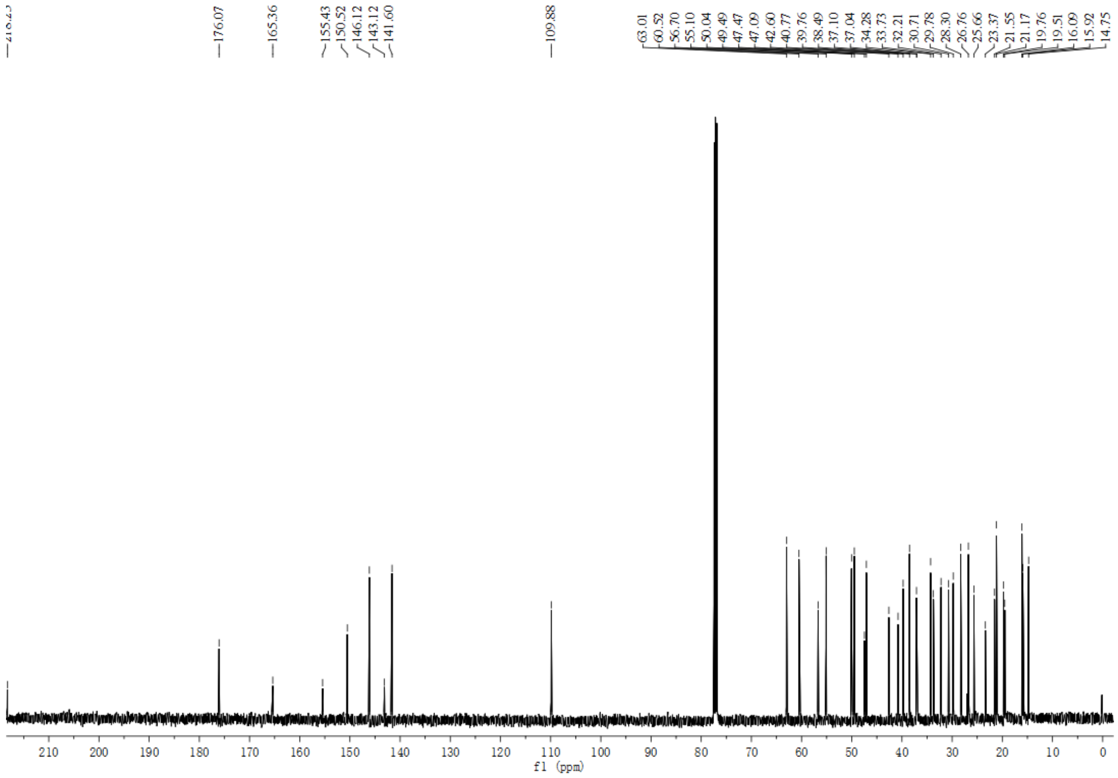

BoA3B ^{13}C-NMR ($CDCl_3$)

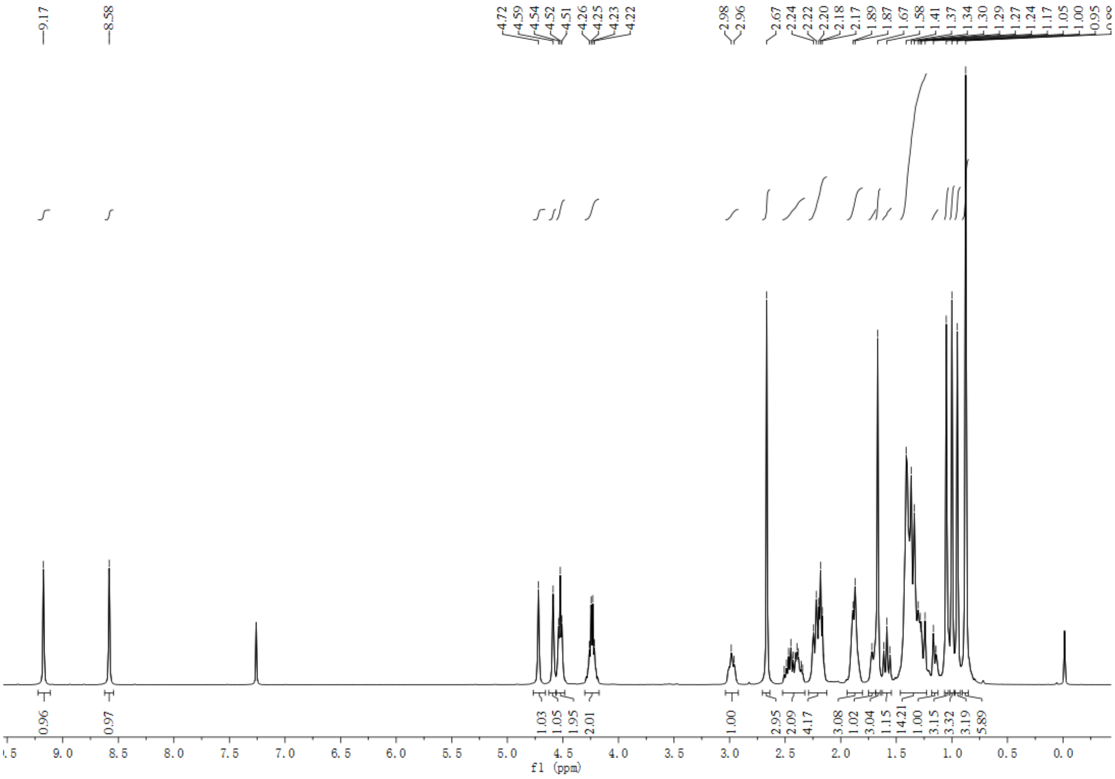

BoA3C ^{1}H-NMR ($CDCl_3$)

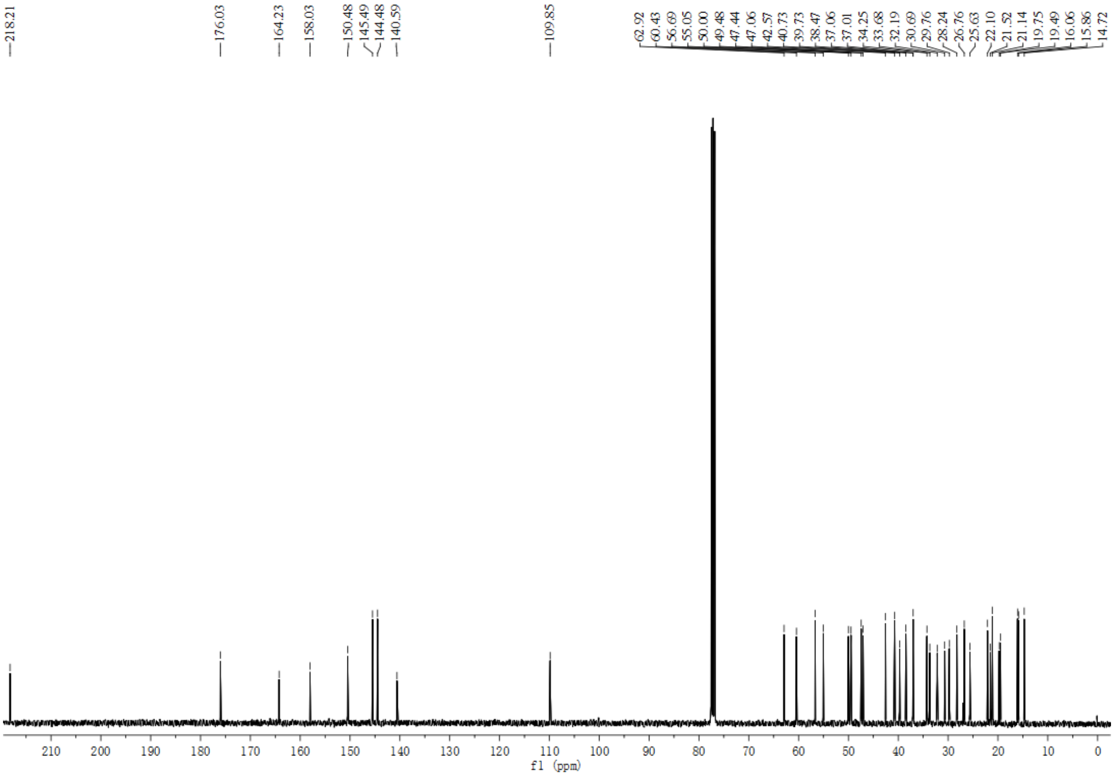

BoA3C ^{13}C-NMR ($CDCl_3$)

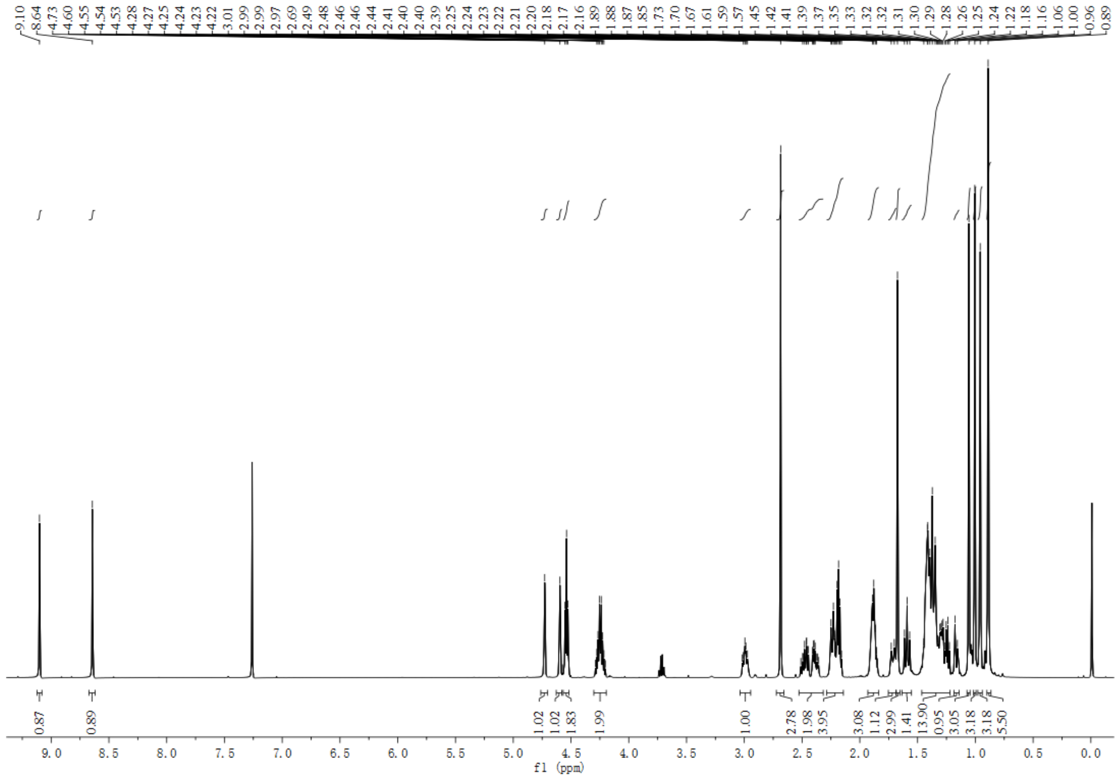

BoA3D ^{1}H-NMR ($CDCl_3$)

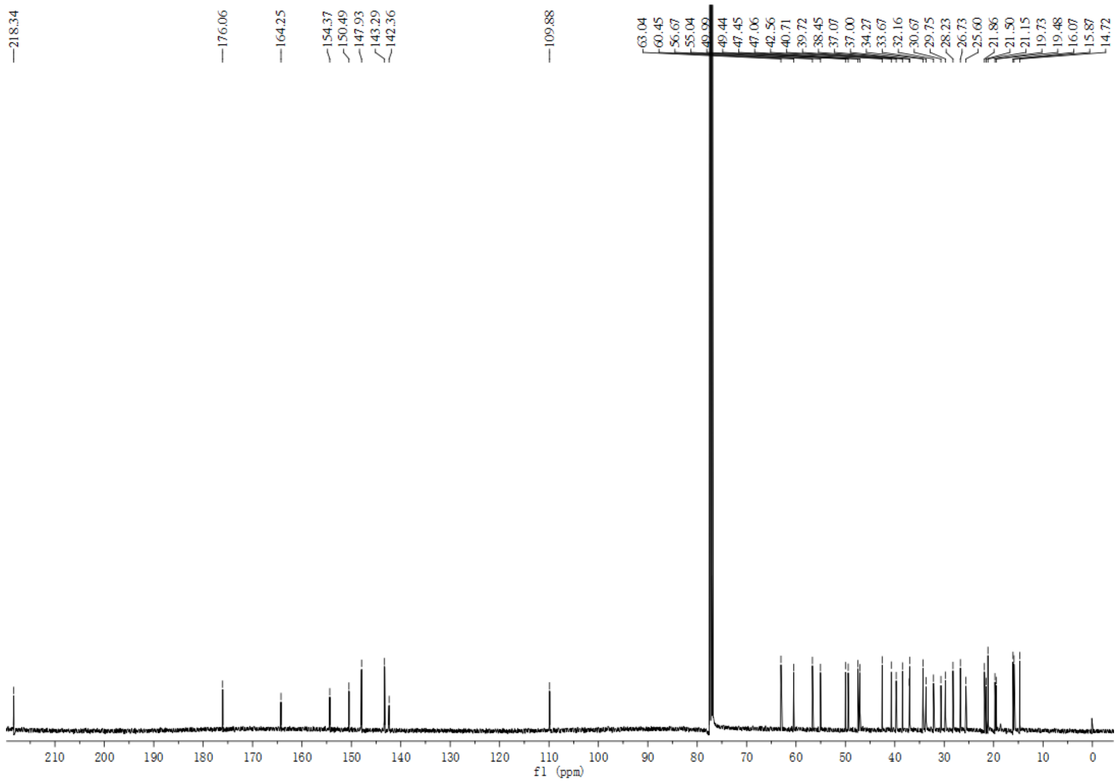

BoA3D ^{13}C-NMR ($CDCl_3$)

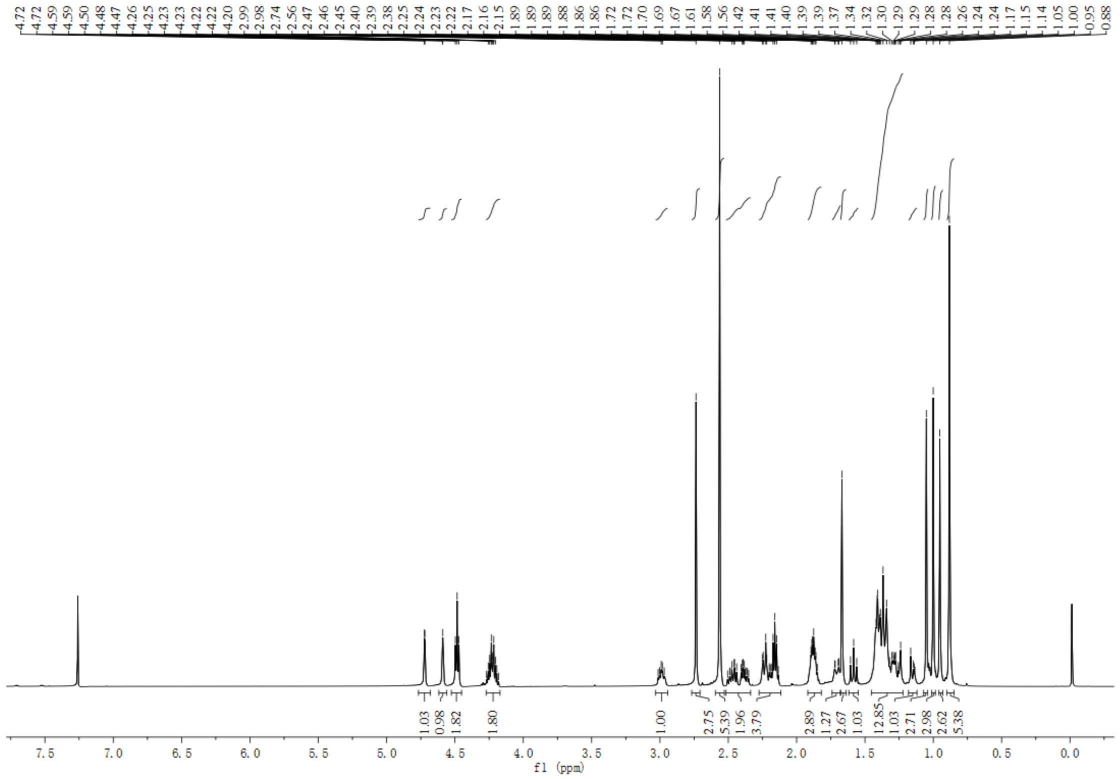

BoA3E ^{1}H-NMR ($CDCl_3$)

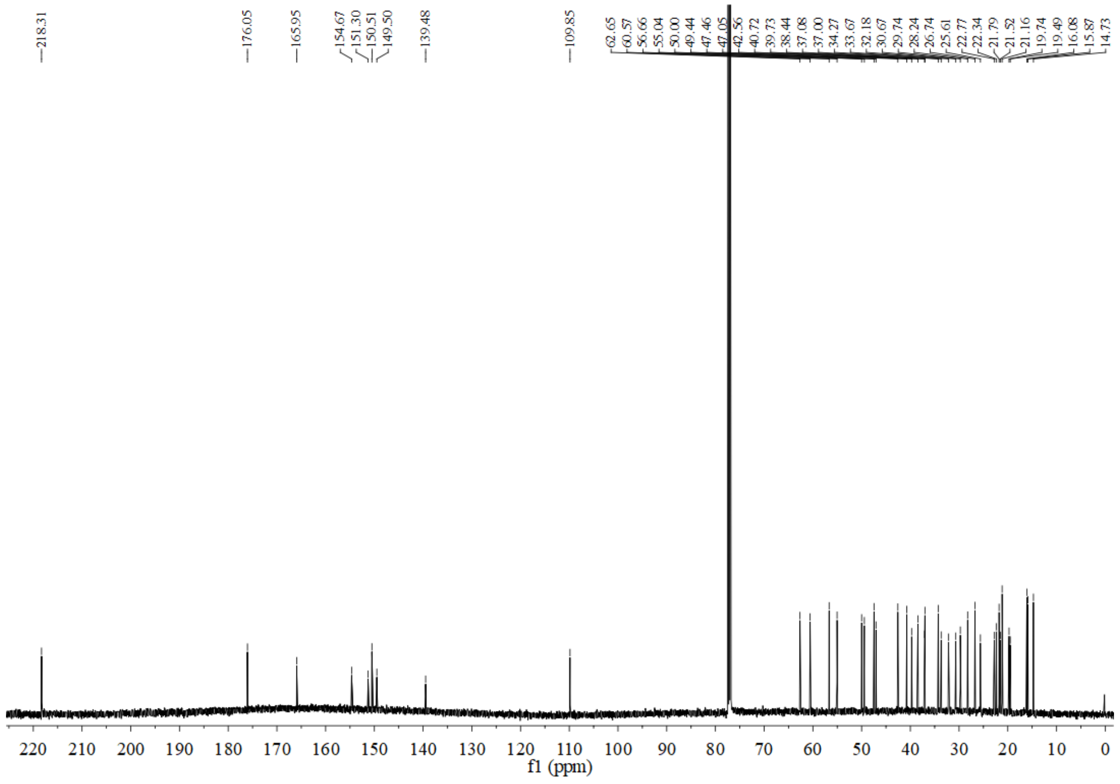

BoA3E ^{13}C-NMR ($CDCl_3$)

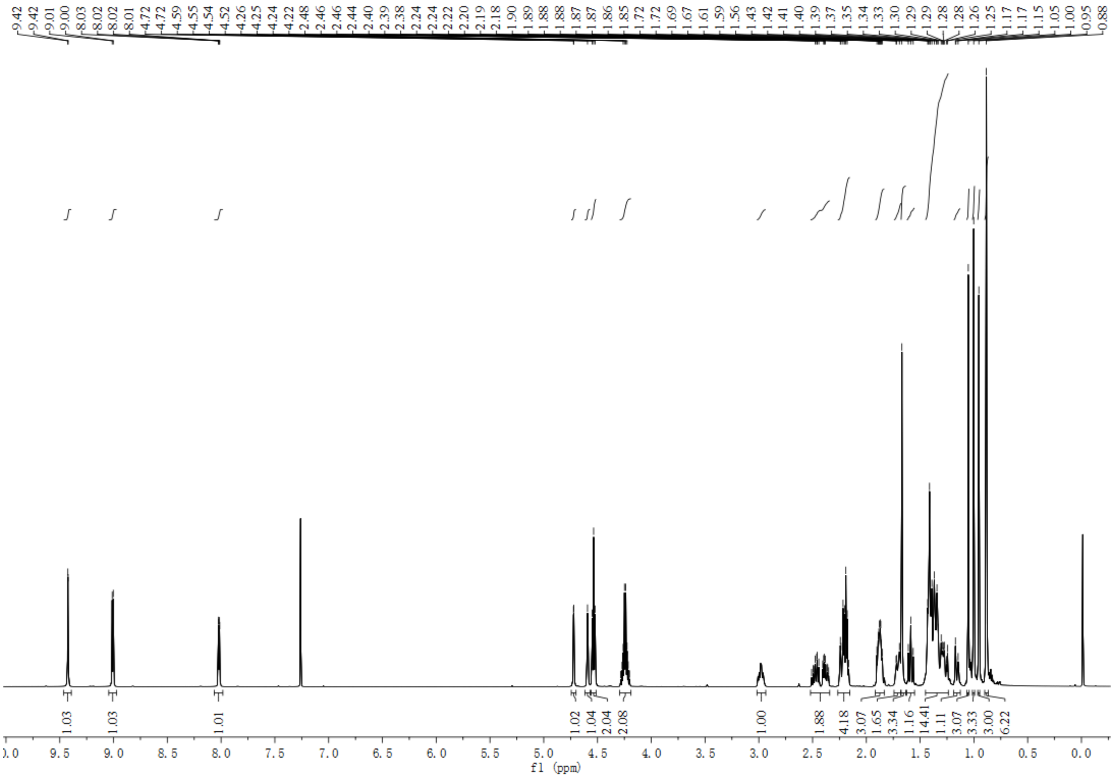

BoA3F ^{1}H-NMR ($CDCl_3$)

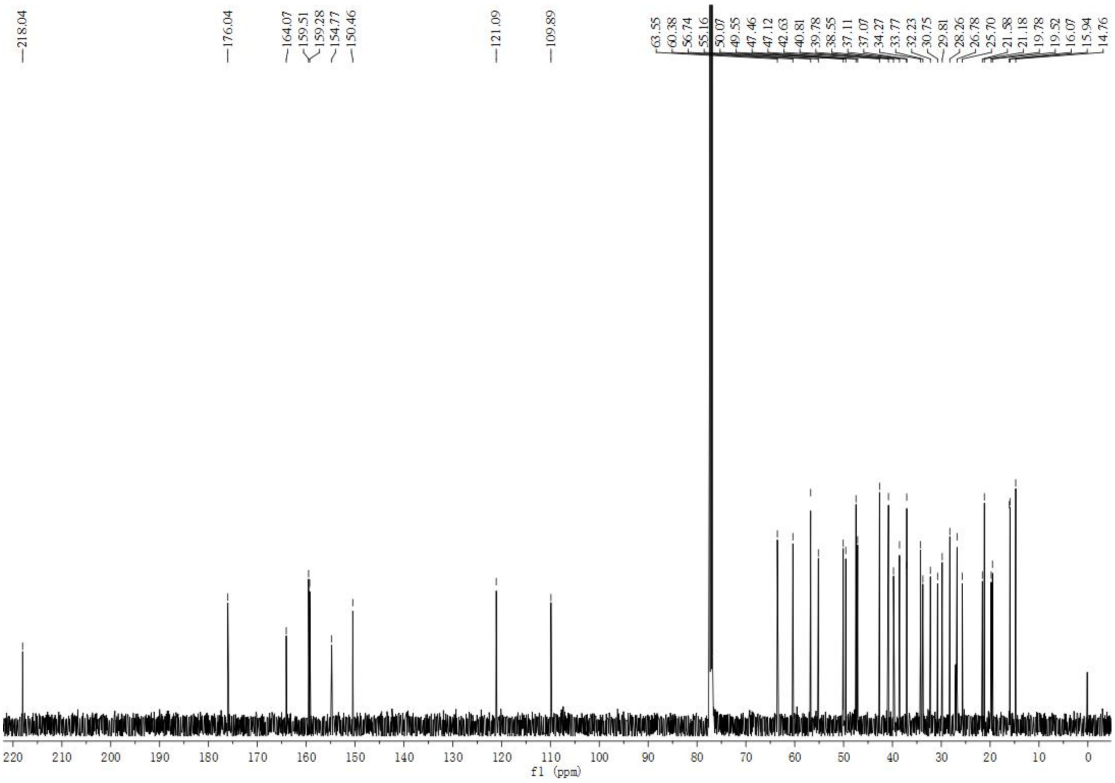

BoA3F ^{13}C-NMR ($CDCl_3$)

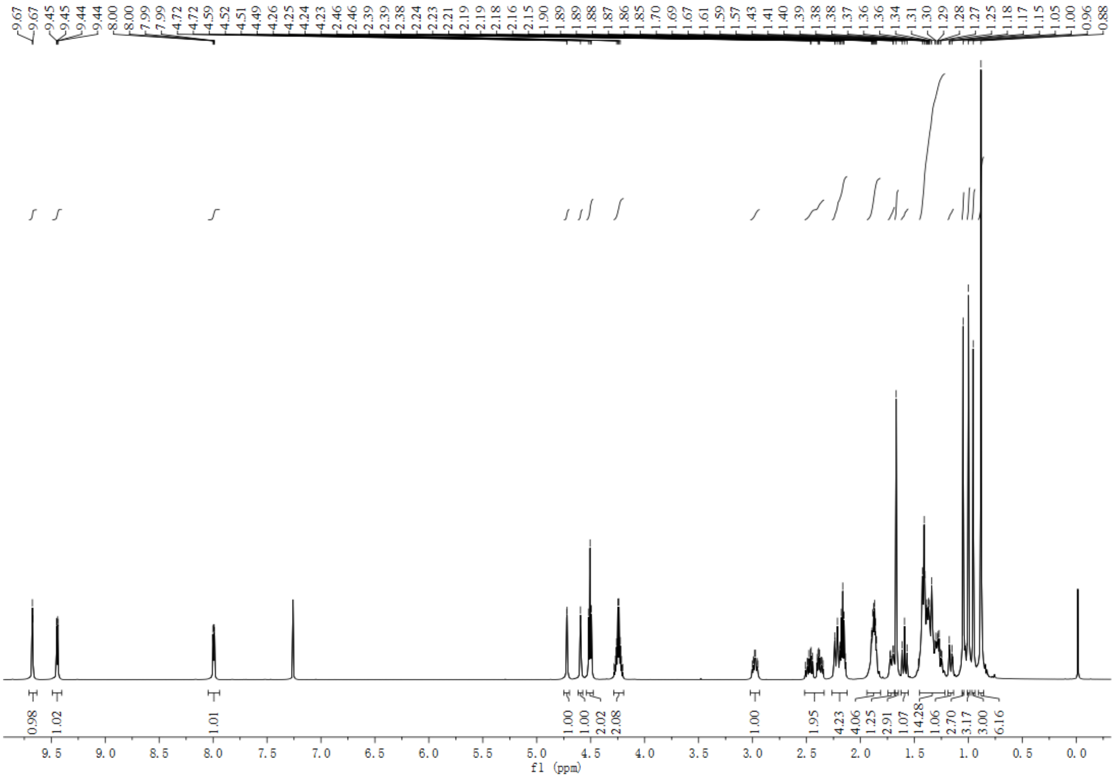

BoA3G ^{1}H-NMR ($CDCl_3$)

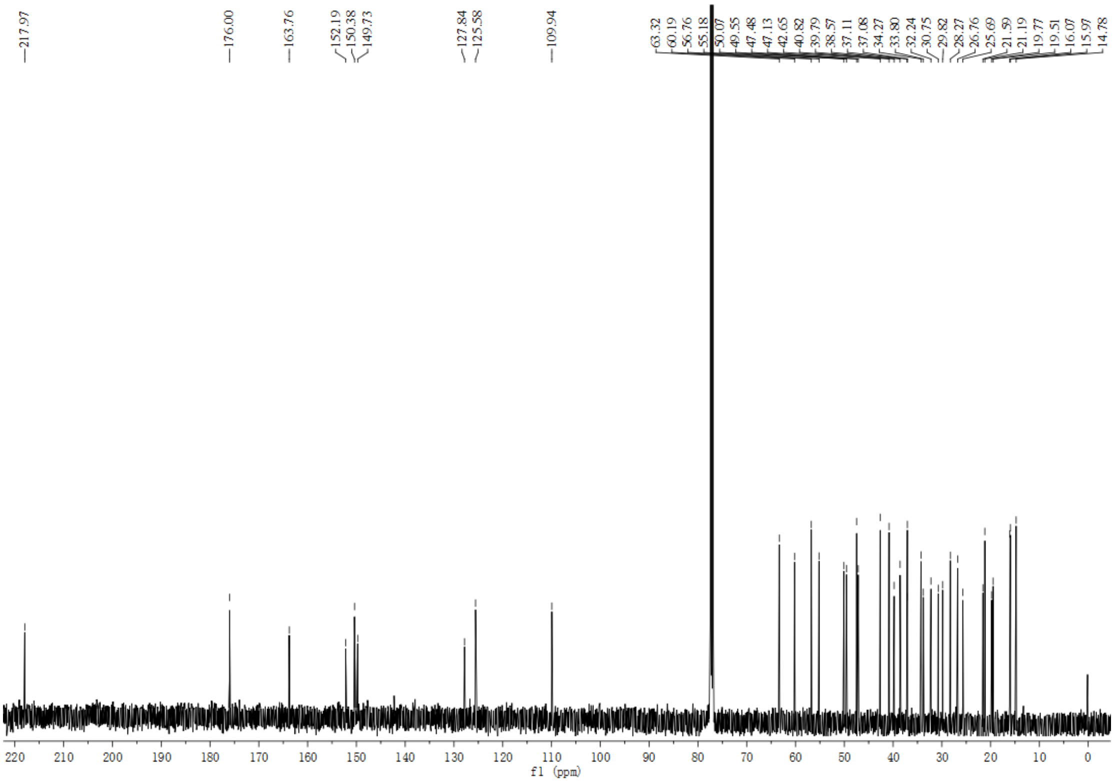

BoA3G ^{13}C-NMR ($CDCl_3$)

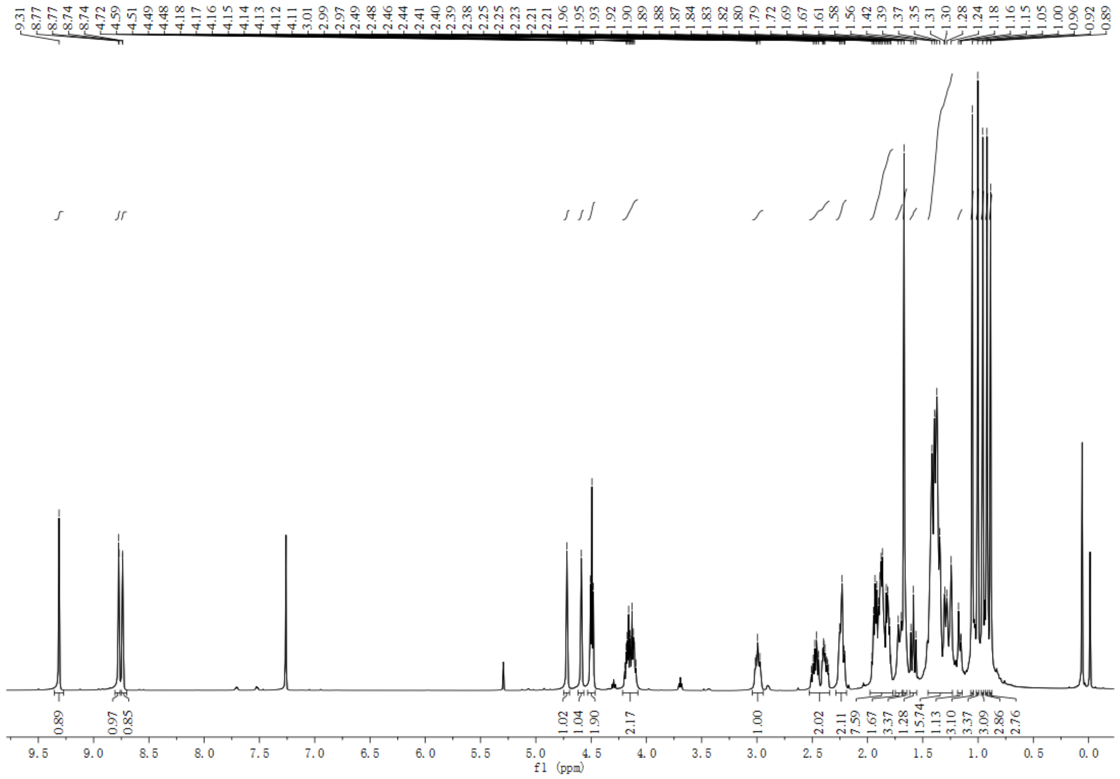

BoA4A ^{1}H-NMR ($CDCl_3$)

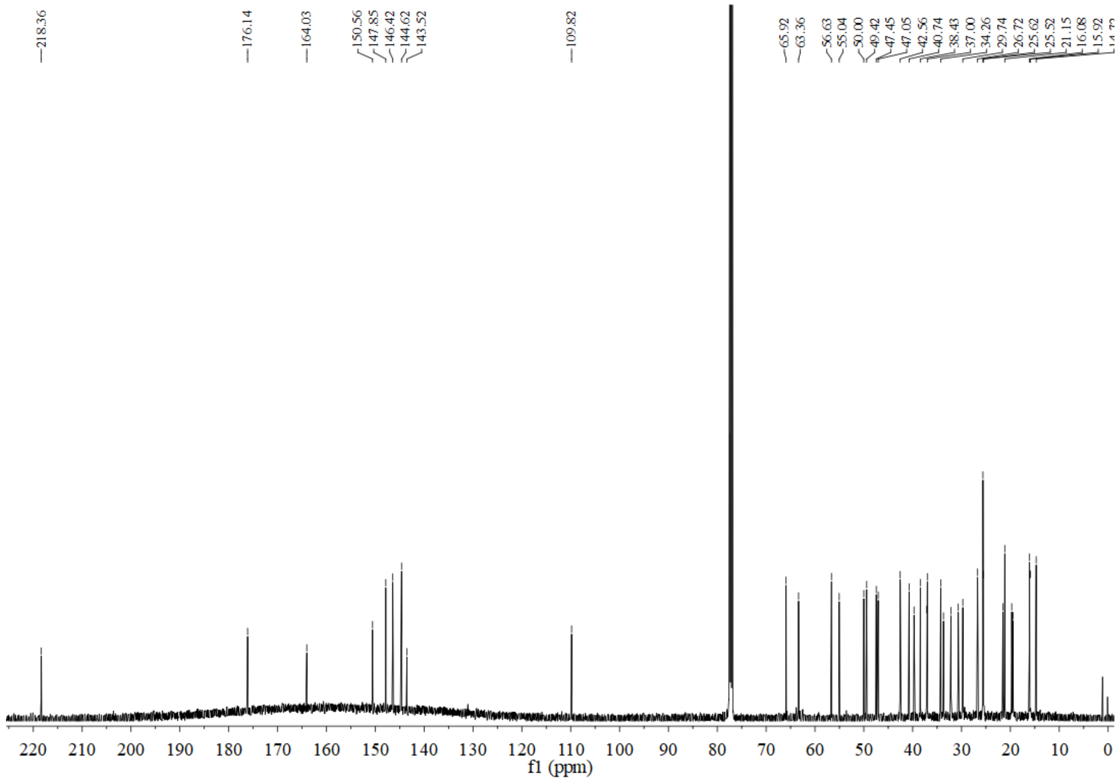

BoA4A ^{13}C-NMR ($CDCl_3$)

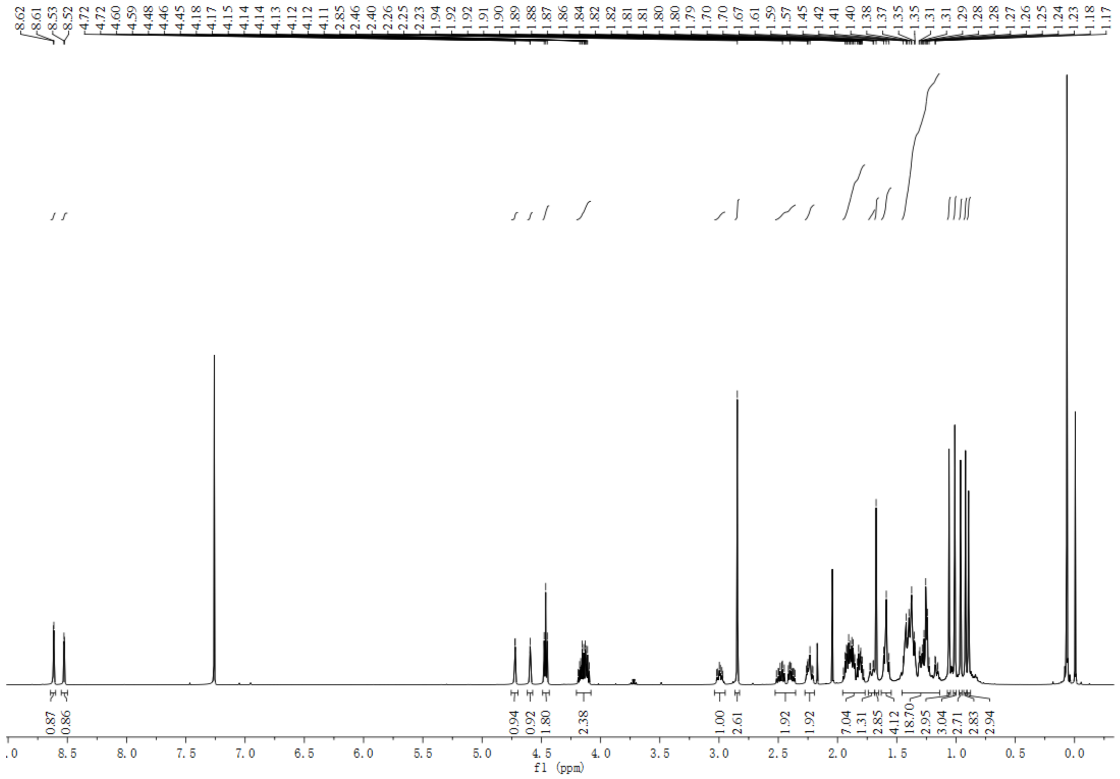

BoA4B ^{1}H-NMR ($CDCl_3$)

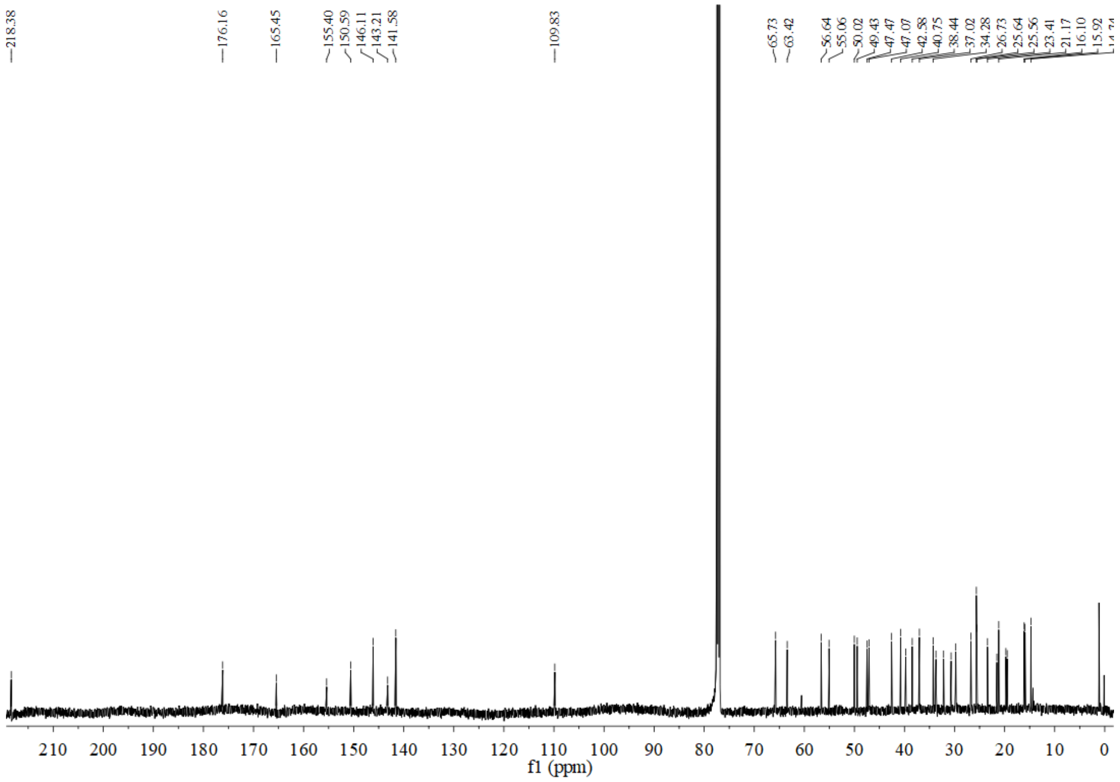

BoA4B ^{13}C-NMR ($CDCl_3$)

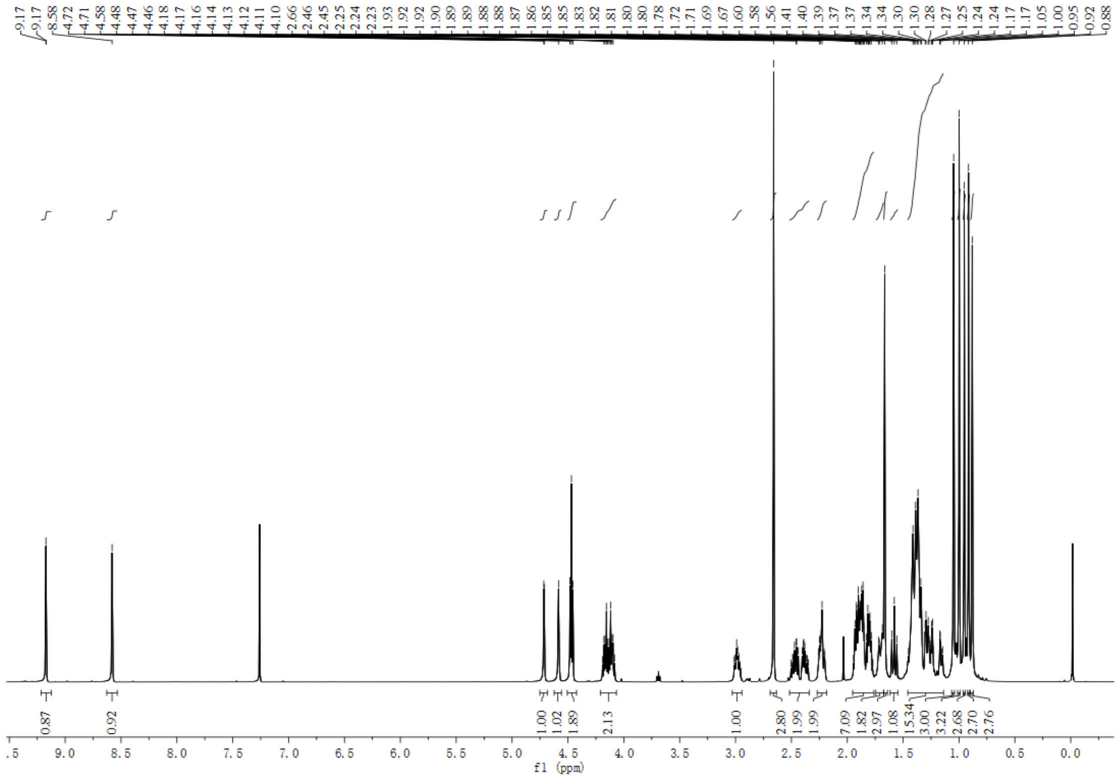

BoA4C ^{1}H-NMR ($CDCl_3$)

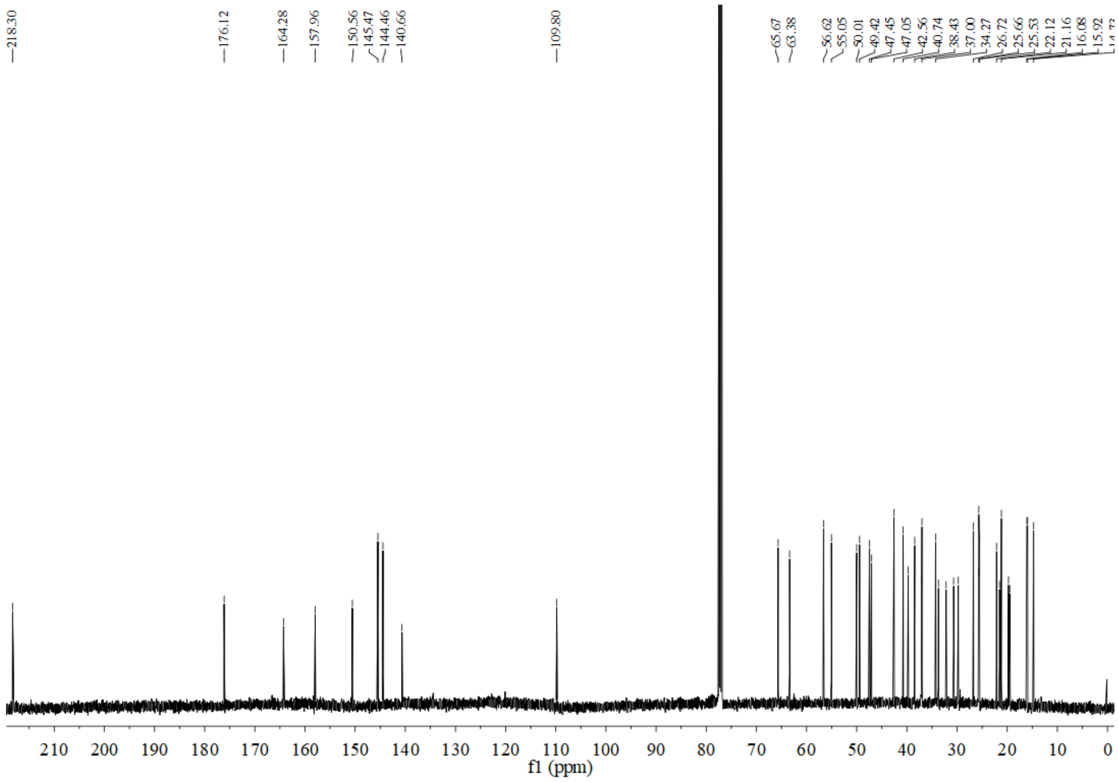

BoA4C ^{13}C-NMR ($CDCl_3$)

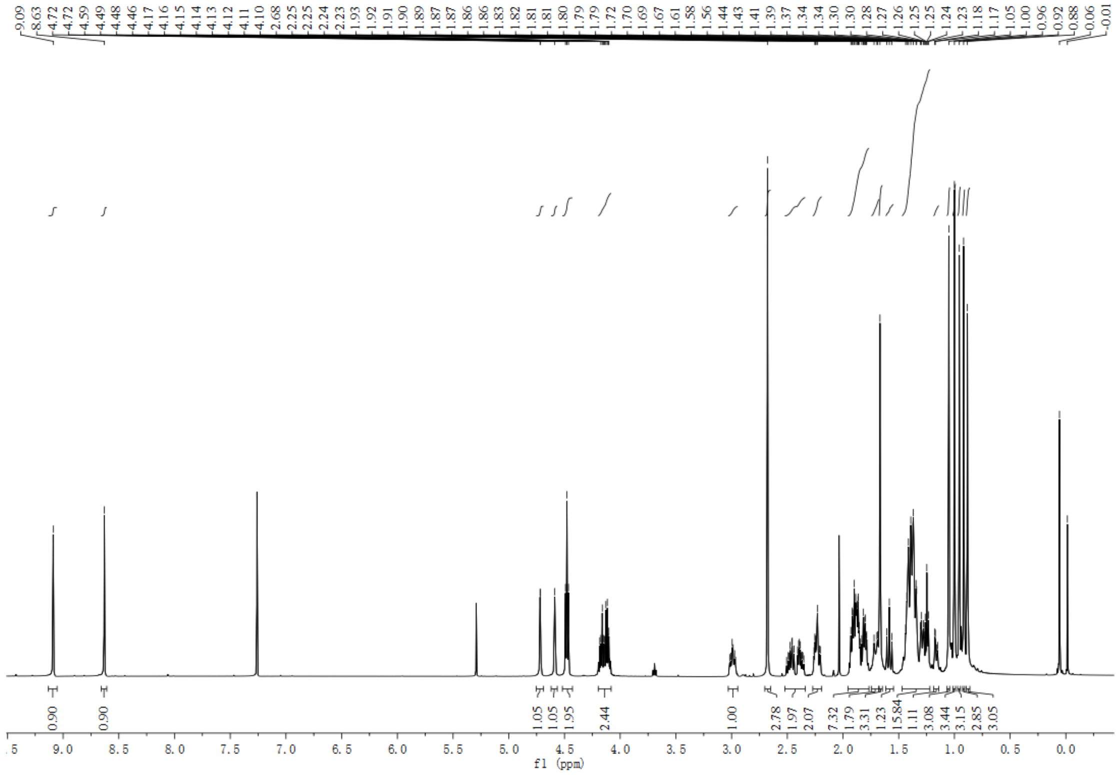

BoA4D ^{1}H-NMR ($CDCl_3$)

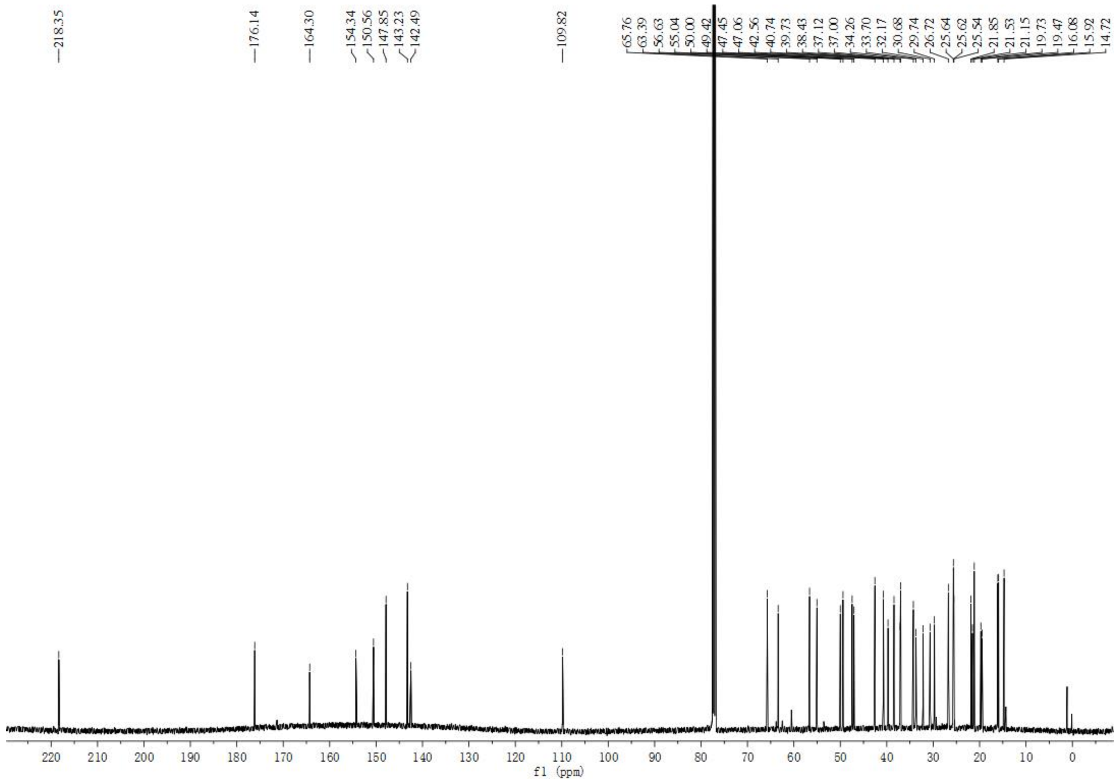

BoA4D ^{13}C-NMR ($CDCl_3$)

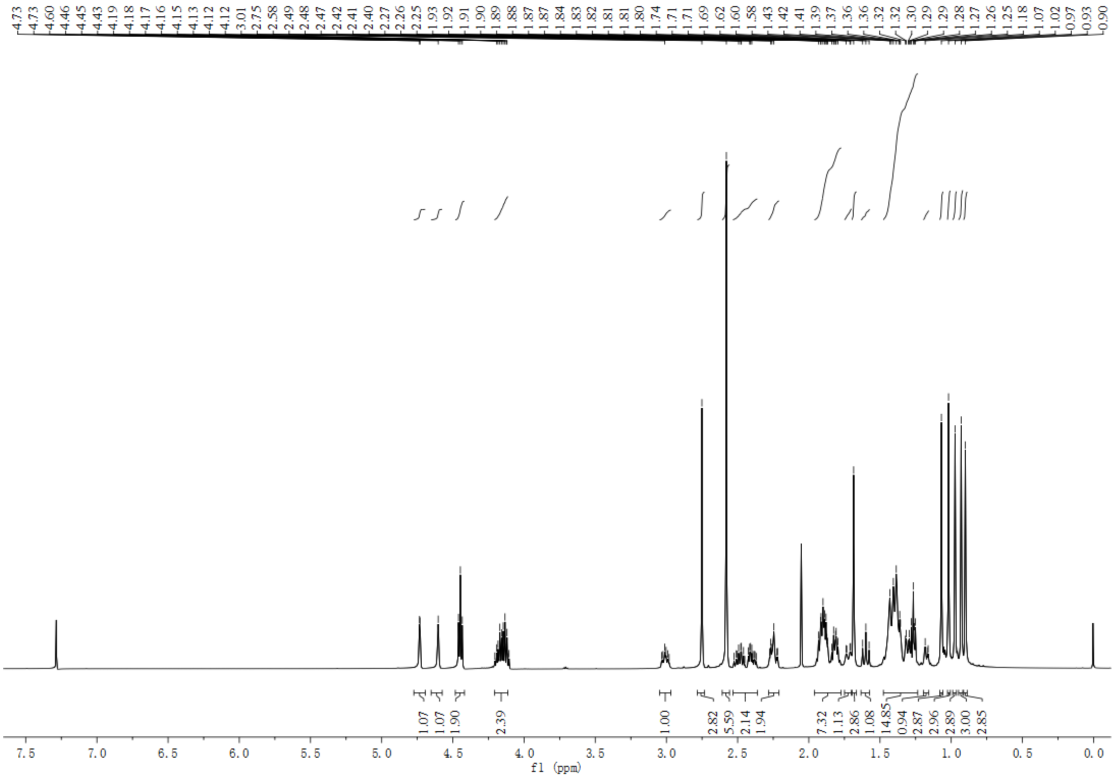

BoA4E ^{1}H-NMR ($CDCl_3$)

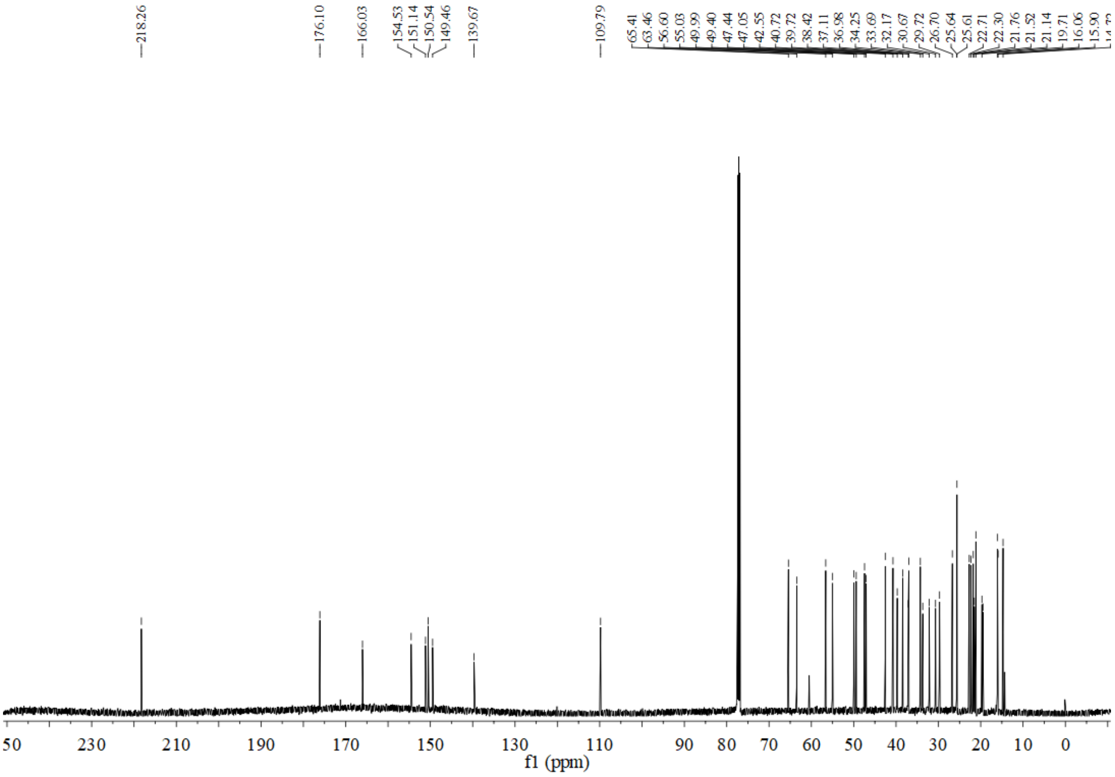

BoA4E ^{13}C-NMR ($CDCl_3$)

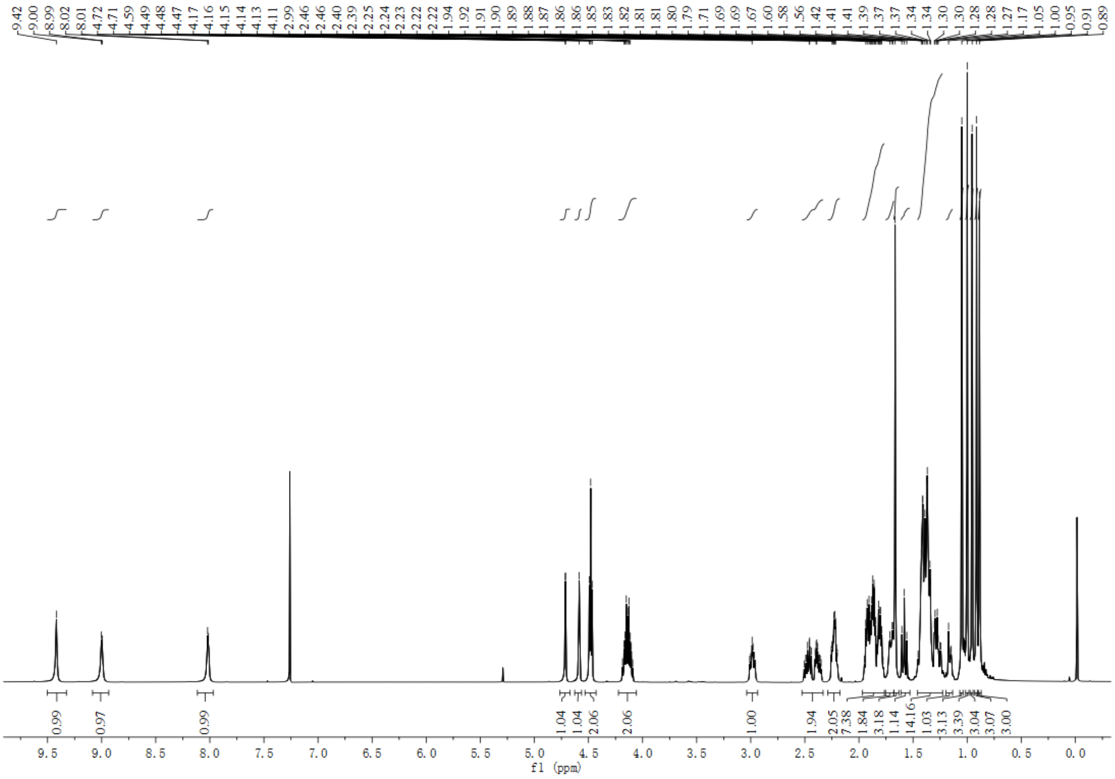

BoA4F ^{1}H-NMR ($CDCl_3$)

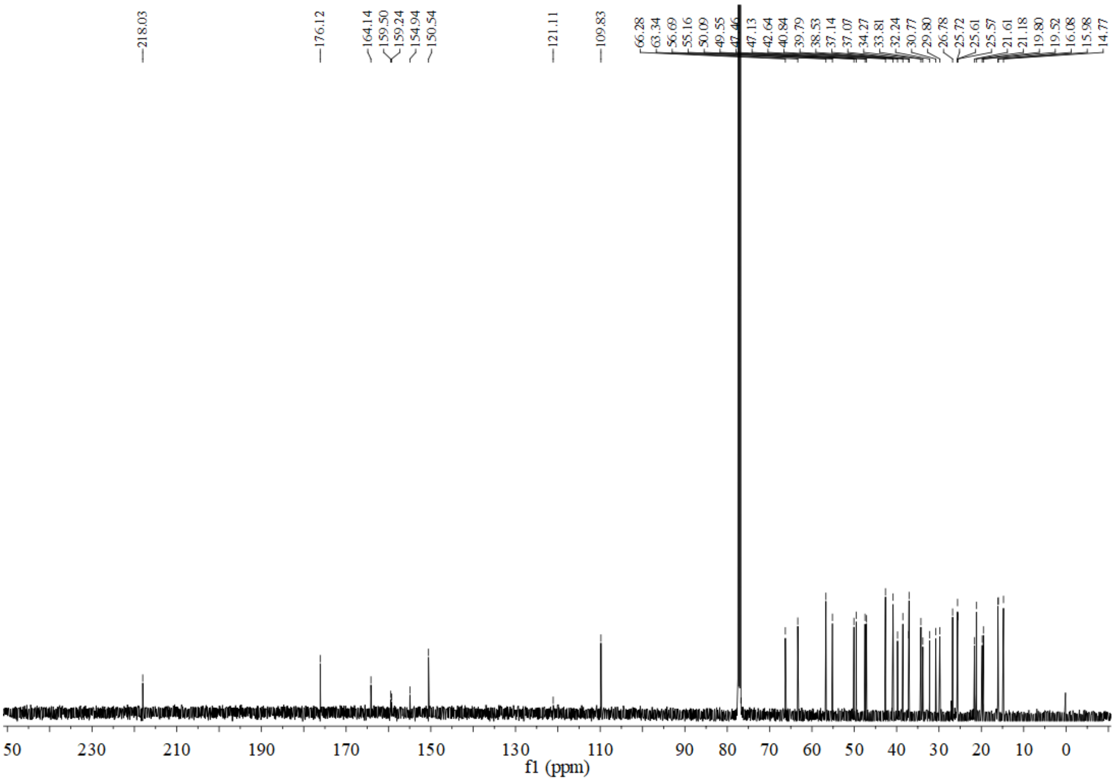

BoA4F ^{13}C-NMR ($CDCl_3$)

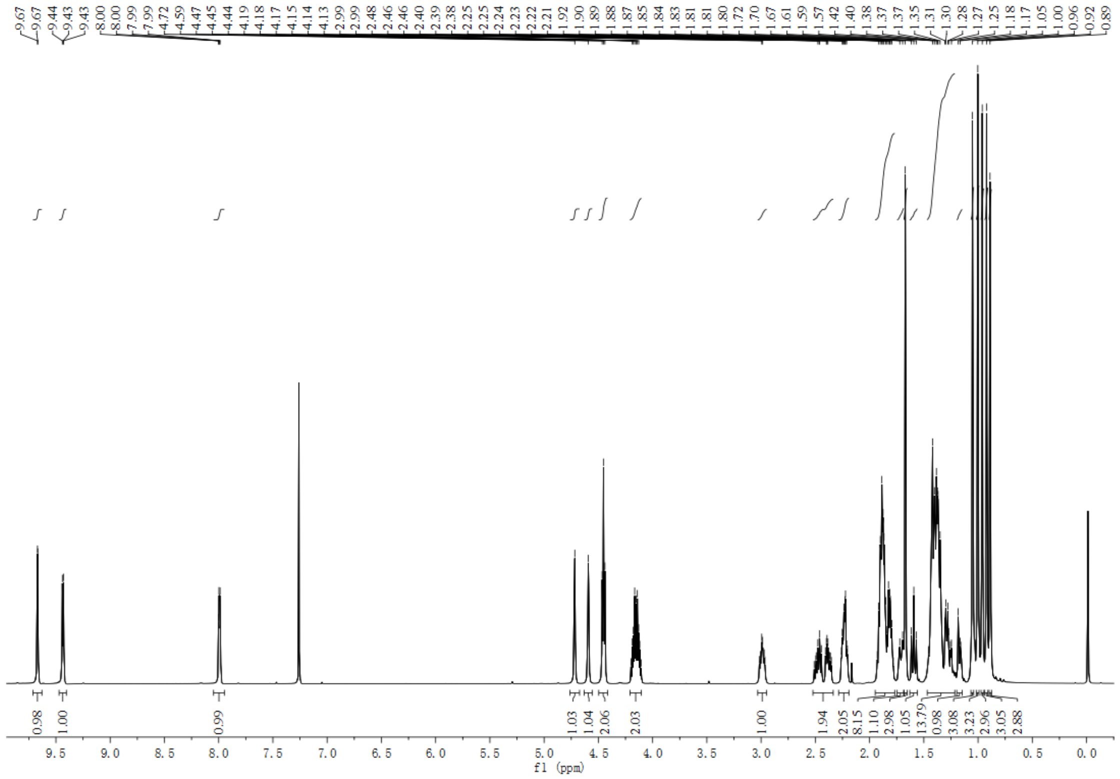

BoA4G ^{1}H-NMR ($CDCl_3$)

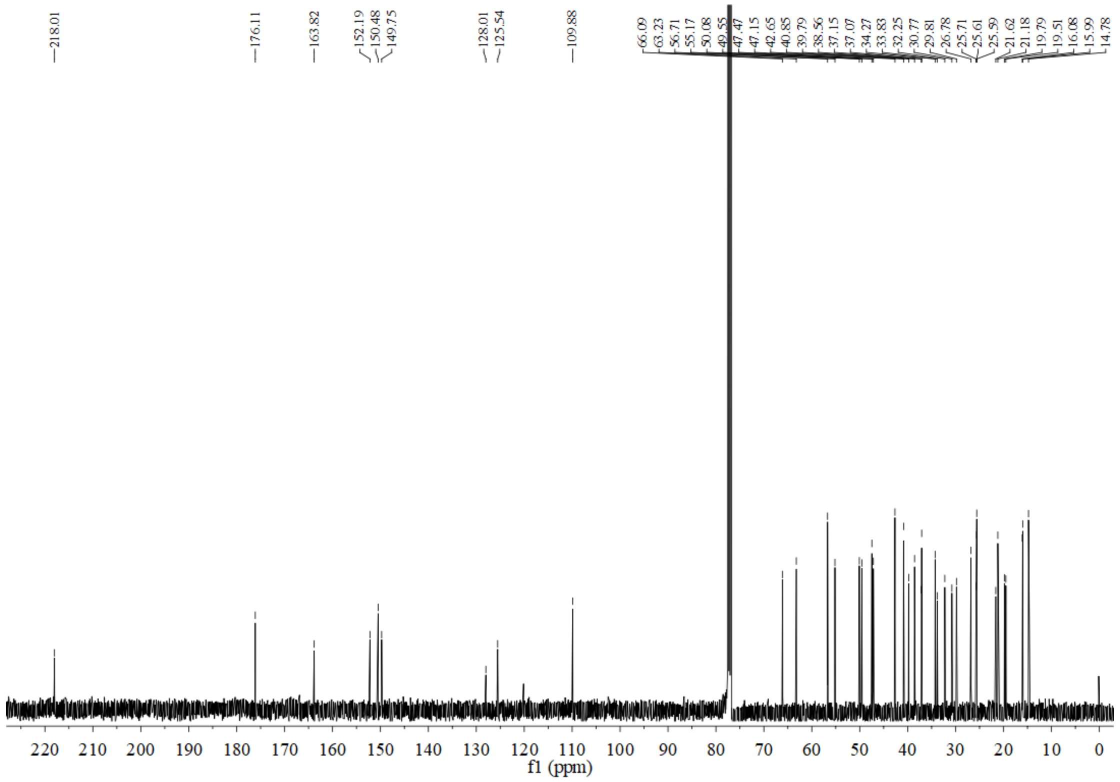

BoA4G ^{13}C-NMR ($CDCl_3$)
